# Euler characteristic curves and profiles: a stable shape invariant for big data problems

**DOI:** 10.1093/gigascience/giad094

**Published:** 2023-11-15

**Authors:** Paweł Dłotko, Davide Gurnari

**Affiliations:** Dioscuri Centre in Topological Data Analysis, Mathematical Institute, Polish Academy of Sciences, Warsaw, 00-656, Poland; Dioscuri Centre in Topological Data Analysis, Mathematical Institute, Polish Academy of Sciences, Warsaw, 00-656, Poland

**Keywords:** topological data analysis, persistent homology, Euler characteristic, distributed computations

## Abstract

Tools of topological data analysis provide stable summaries encapsulating the shape of the considered data. Persistent homology, the most standard and well-studied data summary, suffers a number of limitations; its computations are hard to distribute, and it is hard to generalize to multifiltrations and is computationally prohibitive for big datasets. In this article, we study the concept of Euler characteristics curves for 1-parameter filtrations and Euler characteristic profiles for multiparameter filtrations. While being a weaker invariant in one dimension, we show that Euler characteristic–based approaches do not possess some handicaps of persistent homology; we show efficient algorithms to compute them in a distributed way, their generalization to multifiltrations, and practical applicability for big data problems. In addition, we show that the Euler curves and profiles enjoy a certain type of stability, which makes them robust tools for data analysis. Lastly, to show their practical applicability, multiple use cases are considered.

## Introduction

Topological data analysis since its beginning [1, 2] has brought attention in the data science community. Topological tools, like persistent homology [3] and mapper [2], were used in multiple tasks in material science [4–6], medicine [7], and many more. In time, persistent homology has been successfully integrated with machine learning pipelines, and mapper became an exploratory data analysis tool. In this work, we will extend on the path of persistent homology. With its successes, attempts were made to apply it in task of big data analysis. However, the progress is minimal. While there exists a single distributed implementation [8], it does not scale up and was not extensively used in big data analysis. In practice, mostly various sequential implementations are used [9]. To bypass the problem of too large input, a number of sparsification techniques [10, 11] as well as bootstrap [12] and zigzag [13] approaches were proposed. While they scale up to problems of a certain size, they tend to bypass the big data challenge rather than proposing a solution for it.

In this article, we extend the tool of classical Euler characteristic and Euler characteristic curves. The new contributions include the following:

- A proof of stability of the Euler characteristic curve (ECC) with respect to the 1-Wasserstein distance between persistence diagrams
- A generalization of the ECC to the multiparamenter filtration case, with an arbitrary number of parameters, that we denote as the Euler characteristic profile (ECP)
- An analysis of the stability of such ECPs
- Distributed algorithms to compute the exact ECC for Vietoris–Rips and cubical complexes that can be naturally extended to the multiparameter case. An Python implementation of such algorithms is provided as the scikit-learn [14] compatible package.
- Discussion of methods to compare and vectorize ECCs and ECPs
- Examples of applications of the ECC/ECP to real-world data

Our proposed algorithms are perfectly parallelizable and our software implementation can take advantage of multicore CPUs that are nowadays the standard even in consumer-grade hardware. Moreover, they can also be easily executed on a computer cluster to tackle scenarios when the size of the input data is too large to fit into the system’s memory. While we are not aware of any distributed algorithm to compute ECCs of a Vietoris–Rips complex, Heiss and Wagner [15] describe a streaming algorithm to compute the ECC from cubical complexes, which has also been adapted for GPU computations [16]. While their implementation is very fast, we see no straightforward way to generalize it to the multiparameter filtration case. To the best of our knowledge, the concept of ECPs of arbitrary dimension is novel in the literature. There are, however, some works that focus on the bifiltration case, known as Euler characteristic surfaces. It was used in an applied setting by Roy et al. [17] to analyze drying droplets, but no topological background is provided. Beltramo et al. [18] gave a description of Euler characteristic surfaces in the persistence homology framework and applied it to obtain a descriptor of both pointcloud and image-based data. Moreover, they provided a Python implementation of their algorithms, which, however, requires the input bifiltration to be binned. Chen et al. [19] introduced a time-aware multipersistence Euler–Poincaré surface to describe dynamical networks and proved its weak *L*_1_ stability. A recent preprint by Perez [20] analyzes the stability of Euler and Betti curves of stochastic processes on compact Riemannian manifolds.

## Euler Characteristic Curves (and Profiles)

In this section, we introduce the essential mathematical concepts needed to define Euler characteristic curves and profiles. For an exhaustive presentation, we refer to classic textbooks like [21] and [3].

Definition 1. A *CW* or *cell complex X* is a topological space that can be built up starting from a discrete set *X*^0^ of 0-dimensional cells and then inductively creating the *n*-skeleton *X*^*n*^ by attaching *n*-cells to *X*^*n* − 1^ along their boundary. The process can be stopped at some finite dimension or can continue indefinitely. A subset *A*⊆*X* is a *subcomplex* of *X* if, with each cell of *A*, all its lower-dimensional cells enter *A*.

Remark 1. Since we are interested in applying this machinery to analyze real-world data, we will always assume that our complexes are finite.

While the theory can be built in the general CW complex setting, the algorithms we present in the Algorithms section are specific to 2 different specializations that are used to represent different types of data: simplicial and cubical complexes.

Definition 2. An *abstract simplicial complex* is a finite collection of sets *K* such that σ ∈ *K* and τ⊆σ implies τ ∈ *K*. The sets in *K* are called *simplicies*, and the *dimension* of a simplex is *dim*(σ) = *card*(σ) − 1. We will often refer to 0-simplices as *vertices* and to 1-simplices as *edges*. Given a simplex *s* = {*v*_0_, …, *v*_*k*_}, its *boundary* is $\partial s = \sum _{i=0}^{k} (-1)^i \lbrace v_0,\ldots ,\hat{v_i},\ldots ,v_k\rbrace$, where $\hat{v_i}$ denotes that the vertex *v*_*i*_ is removed from the simplex. Simplices $\lbrace v_0,\ldots ,\hat{v_i},\ldots ,v_k\rbrace _{i=0}^k$ are in the boundary of *s*.

There are different ways of obtaining an abstract simplicial complex from pointcloud data such as the Čech, the Vietoris–Rips, and the Alpha constructions [3], and in the Vietoris–Rips complexes section, we describe the Vietoris–Rips construction.

Definition 3. An *elementary interval* is a subset of $\mathbb {R}$ of the type *I* = [*l, l* + 1] or *I* = [*l, l*], for some integer *l*. The first type is called a nondegenerate interval while the second is a degenerate interval. An *elementary cube C* is a product of elementary intervals *C* = *I*_1_ × ⋅⋅⋅ × *I*_*n*_, and its dimension is the number of nondegenerate intervals in the product. The *boundary* of an elementary interval is ∂[*l, l* + 1] = [*l* + 1, *l* + 1] + [*l, l*] and ∂[*l, l*] = 0. The boundary of an elementary cube is then defined as $\partial C = \partial (I_1 \times \cdots \times I_n) = \sum _{i=1}^n I_1 \times \cdots \times \partial I_i \times \cdots \times I_n$. Similarly to the simplicial complex case, a *cubical complex K* is a collection of elementary cubes closed under operation of taking boundary.

One of most common use cases of cubical complexes involves image data. In section 16 "Cubical complexes"  we describe how to build a filtered cubical complex from an *n*-dimensional image by identifying the image’s pixels with top dimensional cells.

In what follows, we will refer to simplices and cubes as elements of a simplicial or a cubical complex jointly as *cells* in a *cell complex*. A cell τ is said to be a *face* of σ if τ is in the boundary of σ.

Definition 4. Let *K* be a cell complex and *d* a dimension. A *d-chain* is a formal sum of *d*-cells in *K*, namely, *c* = ∑*a*_*i*_σ_*i*_, where the σ_*i*_ are the *d*-cells and the *a*_*i*_ are the coefficients.

There are many possible choices for the group of coefficients. A standard approach in computational topology is to use *modulo 2 coefficients*, that is, the *a*_*i*_ can be either 0 or 1 and satisfy 1 + 1 = 0. (Using modulo 2 coefficients allows us to get rid of the ( − 1)^*i*^ in the definition of the boundary in 2.) Other options include integer, rational, or real coefficients.

Two *d*-chains can be added component-wise. Namely, given *c* = ∑*a*_*i*_σ_*i*_ and *c*′ = ∑*b*_*i*_σ_*i*_, *c* + *c*′ = ∑(*a*_*i*_ + *b*_*i*_)σ_*i*_. Therefore, we can define the *group of d-chains*  $\boldsymbol {C}_d = \boldsymbol {C}_d(K)$. The boundary of a *d*-chain is the sum of the boundaries of its cells ∂*c* = ∑*a*_*i*_∂σ_*i*_, which is a (*d* − 1)–chain. Since the boundary commutes with the addition operation, we can define, for each dimension *d*, the *boundary homomorphism*  $\partial _d : \boldsymbol {C}_d \rightarrow \boldsymbol {C}_{d-1}$.

A *d-cycle* is a *d*-chain with empty boundary ∂*c* = 0. A *d-boundary* is a *d*-chain that is the boundary of a (*d* + 1)–chain. Since ∂ commutes with addition, we have the *group of d-cycles*  $\boldsymbol {Z_d} = \boldsymbol {Z}_d(K)$ and the *group of d-boundaries*  $\boldsymbol {B_d} = \boldsymbol {B}_d(K)$. It is a fundamental result that ∂_*d*_∂_*d* + 1_*c* = 0 for every dimension *d* and every (*d* + 1)–chain *c*. This means that the boundary of a boundary is always zero; in other words, $\boldsymbol {B}_d$ is a subgroup of $\boldsymbol {Z}_d$. This leads to the following definition.

Definition 5. The *d*th *homology group* is the *d*th cycle group modulo the *d*th boundary group, $\boldsymbol {H}_d = \boldsymbol {Z}_d / \boldsymbol {B}_d$. The *d*th *Betti number* is the rank of this group, $\beta _d = \text{rank}(\boldsymbol {H}_d)$.

Definition 6. Let *K* be a cell complex. A *filtration* of *K* is a sequence of nested subcomplexes ∅ = *K*_0_⊆*K*_1_⊆⋅⋅⋅⊆*K*_*n*_ = *K*. Such a sequence is finite for finite complexes. It can be obtained by means of a *filtration function* over *K*, a monotonic nondecreasing function $f: K \rightarrow \mathbb {R}$ such that *f*(τ) ≤ *f*(σ) if τ is a face of σ. Note that every *sublevel set K*_*t*_ = *f*^−1^( − ∞, *t*] is a subcomplex of *K* for every $t \in \mathbb {R}$.

For each dimension *d*, such a filtration corresponds to a sequence of homology groups $0 = \boldsymbol {H}_d(K_0) \rightarrow \boldsymbol {H}_d(K_1) \rightarrow \cdots \rightarrow \boldsymbol {H}_d(K_n) = \boldsymbol {H}_d(K)$. For every *i* < *j*, the homomorphism $f_d^{i,j} : \boldsymbol {H}_d(K_i) \rightarrow \boldsymbol {H}_d(K_j)$ is induced from the inclusion map of *K*_*i*_ into *K*_*j*_.

Definition 7. The *d*th *persistent homology groups* are the images of the homomorphisms $\boldsymbol {H}_d^{i,j} = \text{im}f_d^{i,j}$. The ranks of these groups are the *d*th *persistent Betti numbers*  $\beta _d^{i,j} = \text{rank}(\boldsymbol {H}_d^{i,j})$.

Intuitively, the *d*th persistent Betti number $\beta _d^{i,j}$ counts how may homology classes of *K*_*i*_ are still present in *K*_*j*_. There are 2 scenarios in which a homology class from *K*_*i*_ may not be present in *K*_*j*_—it may become trivial or identical (homologous) to a class that was created earlier.

Definition 8. The *k*th-dimensional *persistence diagram* of a filtered complex *K, Dgm*_*k*_(*K*) is a multiset of points in the extended real plane $(\mathbb {R} \cup \lbrace \infty \rbrace ) \times (\mathbb {R} \cup \lbrace \infty \rbrace )$. The multiplicity of each point (*b, d*) indicates the number of independent *k*-dimensional classes that are born at filtration value *b* and die at filtration value *d*.

All the points on the diagonal are always included, with countable multiplicity, in a persistence diagram, in order to make sense of the following.

Definition 9. A *matching* of 2 persistence diagrams *C* and *D* is a bijection η: *C* → *D* possibly to or from points on the diagonal.

Definition 10. The 1-Wasserstein distance between two *k*-dimensional persistence diagrams *C, D* is

\begin{eqnarray*}
W_1(C, D) = \inf _{\eta :C \rightarrow D} \sum _{(b,d) \in C} \left \|(b,d) - \eta (b, d) \right \|_\infty ^1, \end{eqnarray*}

where η is a matching of *C* and *D*.

Definition 11. The *Euler characteristic* of a cell complex *K* is the alternating sum of the number of its cells in each dimension

\begin{eqnarray*}
\chi (K) = \sum _d (-1)^d |K^d| \quad . \end{eqnarray*}

where *K*^*d*^ denotes the *d-*dimensional cells in *K*. Thanks to the Euler–Poincaré formula, the Euler characteristic can also be expressed as the alternating sum of the Betti numbers, the ranks of the cell complex’s homology groups: χ(*K*) = ∑_*d*_( − 1)^*d*^β_*d*_(*K*) [3].

Definition 12. Let us consider a filtered complex *K* with filtration function $f: K \rightarrow \mathbb {R}$. We can define its *Euler characteristic curve* as a function that assign an Euler number χ for each filtration level $t \in \mathbb {R}$
 

\begin{eqnarray*}
ECC(K, t) = \chi (K_t) \quad . \end{eqnarray*}

Recall that *K*_*t*_ = *f*^−1^( − ∞, *t*] is a subcomplex of *K* for every $t \in \mathbb {R}$.

We are now interested in extending the concept of the Euler characteristic curve to the more general multidimensional persistence setting [22]. In order to do so, we need to generalize Definition 6 to families of nested complexes indexed by posets. While multidimensional persistence is a vibrant and active research topic, in this article, we will only make use of the basic concepts. We refer the interested reader to [23] for a modern introduction to the topic.

Definition 13. Let *K* be a cell complex and *P* a poset. A *P-indexed filtration* on *K* is a family of nested complexes such that *K*_*x*_ is a subcomplex of *K* for each *x* ∈ *P*, and *K*_*x*_⊆*K*_*y*_ whenever *x* ≤ *y*. If *P* = *T*_1_ × ⋅⋅⋅ × *T*_*n*_ where each *T*_*i*_ is a totally ordered set, we call a *multiparameter* or *n-parameter filtration*.

It is a natural question to ask whether the idea of sublevel sets of a filtration function could be extended too. In general, this is not the case. It can be achieved only when each cell of *K* first appears in the filtration at some unique minimal index in *P*.

Definition 14. Let *K* be a cell complex, *P* a poset, and *f* a function *f: K* → *P*. The *sublevel filtration* of *f* is a family of complexes of the type

\begin{eqnarray*}
K_p = \lbrace \sigma \in K \, | \, f(\sigma ) \le p \rbrace \, . \end{eqnarray*}

A filtration isomorphic to a sublevel filtration is said to be *1-critical*. A filtration that is not 1-critical is said to be *multicritical*.

Definition 15. The ECP of a *P*-filtered complex *K* is a function that is assigned to any value *p* ∈ *P* the Euler characteristic of the corresponding subcomplex *K*_*p*_.

\begin{eqnarray*}
ECP(K, p) = \chi (K_p) \quad . \end{eqnarray*}

For the rest of the article, we will focus on the case $P=\mathbb {R}^n$.

Remark 2. The 2-dimensional ECP already appeared in the literature, and it is known as Euler characteristic surface [17–19]. It was, however, defined only for the Cartesian product of two 1-parameter filtrations, and it is treated as a matrix in the following way. Given a bifiltering function $F: K \rightarrow \mathbb {R}^2$ over *K* and a set of threshold values $I = \lbrace (a_i, b_j) \, | \, 1 \le i \le m , 1 \le j \le n\rbrace$, the Euler characteristic surface is the *m* × *n* integer valued matrix *S* whose entries are *S*_*ij*_ = χ(*K*_*ij*_) = χ(*F*^−1^(( − ∞, *a*_*i*_] × ( − ∞, *b*_*j*_]). This matrix representation corresponds to sampling the 2-dimensional profile on the grid given by *I*. In general, the choice of such grid is not unique, and the spacing of such grid may not be constant. This makes it difficult to define a general notion of distance between Euler characteristic surface matrices. For this reason ,we think it is more natural to define the Euler characteristic profile as a function like in 15 and look for stability results in this setting.

## Stability of Euler Characteristic Curves and Profiles

The goal of this section is to find a bound for the distance between Euler characteristic curves by some known topological quantity of the pointcloud that is robust with respect to small perturbations of the pointcloud. This way, the stability of Euler characteristic curves is obtained.

### Euler characteristic curves

**Figure 1:**
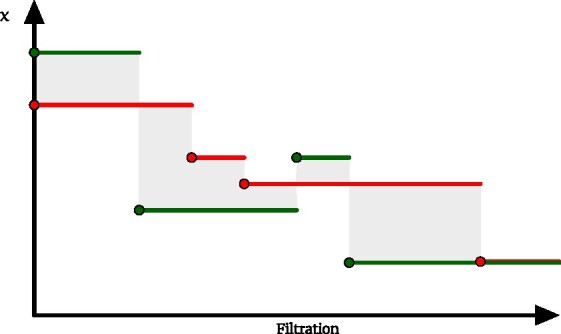
Two Euler characteristic curves in red and green. The absolute value of their difference is highlighted in shaded gray.

Since ECCs are are piecewise constant functions, we consider the *L*_1_ distances between them (Fig. 1).

Definition 16. Let *K*_1_ and *K*_2_ be 2 filtered cell complexes. The *L*_1_ distance between their Euler characteristic curves is

\begin{eqnarray*}
{\left \|ECC(K_1, t) - ECC(K_2, t) \right \|_1} = {\int_{\mathbb R} \left |ECC(K_1, t) - ECC(K_2, t) \right |} dt \quad . \end{eqnarray*}

The proof presented in this section is inspired by the stability result for persistence functions by Chung and Lawson [24], who analyzed the stability of a wide class of persistence curves and obtained a general bound (see Theorem 1 in [24]). However, trying to specialize this result to the simple Betti curve case leads to a term that depends on the number of points in the persistence diagram. Hence, the authors claim that Betti curves are unstable.

We will instead carry out the proof focusing exclusively on Betti curves, and by doing so, a stability result can be obtained.

Definition 17. Let *K* be a cell complex with filtration function *f*. Its *k*th Betti curve is a function that assigns to each filtration level the *k*th Betti number of the corresponding subcomplex.

\begin{eqnarray*}
\beta _k(K, t) = \beta _k(K_t) \quad . \end{eqnarray*}

Let now *D* be the *k*-dimensional persistence diagram obtained from a filtered complex *K*. The fundamental lemma of persistent homology [3] states that the *k*th Betti number of the subcomplex *K*_*t*_ can be obtained by counting the points in the diagram that lie in the box $(x,y) \, | \, x \le t < y$,


\begin{eqnarray*}
\beta _k(K_t) = \#[(b,d) \in D \, | \, b \le t < d] . \end{eqnarray*}

We can reformulate this statement by assigning to each point (*b, d*) in the diagram its indicator function in the interval [*b, d*), *I*_[*b, d*)_(*t*) = 1 if *t* ∈ [*b, d*) and 0 otherwise. These indicator functions are exactly the bars in the barcode representation. By doing so, we can define the *k*-dimensional Betti curve as the step function obtained by summing up all these indicator functions.

Definition 18. The *k*th Betti curve for a persistence diagram *D* with finitely many off-diagonal points is

\begin{eqnarray*}
\beta _k(D, t) = \sum _{(b,d) \in D} I_{[b,d)}(t) . \end{eqnarray*}

Proposition 1. Let *C* and *D* be 2 *k*-dimensional persistence diagrams. Their Betti curves are stable with respect to the 1-Wasserstein distance,

(1) \begin{eqnarray*}
\left \| \beta _k(C, t) - \beta _k(D, t)\right \| _1 \le 2W_1(C,D). \end{eqnarray*}

Proof. Let us consider 2 *k*-dimensional persistence diagrams *C, D* and assume the optimal matching under the 1-Wasserstein distance is known. Moreover, let us index the points in each diagram as $(b_i^C, d_i^C)$ and $(b_i^D, d_i^D)$ so that points with matching indices are paired under the optimal matching. The case when points from 1 diagram are matched to the diagonal is described in case 2. We can then write the difference between the 2 Betti curves as the following:

\begin{eqnarray*}
\left \| \beta _k(C, t) - \beta _k(D, t)\right \| _1 &= \left \| \sum _i I_{[b_i^C,d_i^C)}(t) - I_{[b_i^D,d_i^D)} (t)\right\| _1 \\ & \le \sum _i \left \| I_{[b_i^C,d_i^C)}(t) - I_{[b_i^D,d_i^D)} (t)\right \| _1 . \end{eqnarray*}

**Figure 2:**
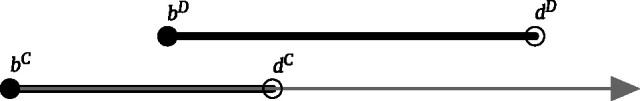
Case 1.

Let us focus on a single term of the sum, $\left \| h_i(t)\right \| = \left \| I_{[b_i^C,d_i^C)}(t) - I_{[b_i^D,d_i^D)} (t)\right \| _1$. Then, one of the following cases has to hold:

Case 1:  $b_i^C \le b_i^D \le d_i^C \le d_i^D$ (Fig. 2).

\begin{eqnarray*}
\left \| h_i(t)\right \| &= \int _{b_i^C}^{b_i^D}{\left | I_{[b_i^C,d_i^C)}(t)\right | dt} + \int _{b_i^D}^{d_i^C}{\left | I_{[b_i^C,d_i^C)}(t) - I_{[b_i^D,d_i^D)}(t)\right | dt } \\ & \qquad +\int _{d_i^C}^{d_i^D}{\left | I_{[b_i^D,d_i^D)}(t)\right | dt} \\ &= \int _{b_i^C}^{b_i^D}{\left | 1\right | dt} + \int _{b_i^D}^{d_i^C}{\left | 1-1\right | dt } + \int _{d_i^C}^{d_i^D}{\left | 1\right | dt} \\ &= \left | b_i^D - b_i^C\right | + \left | d_i^D - d_i^C\right | \\ &\le 2 \max (\left | b_i^D - b_i^C\right | , \left | d_i^D - d_i^C\right | ) . \end{eqnarray*}

**Figure 3:**
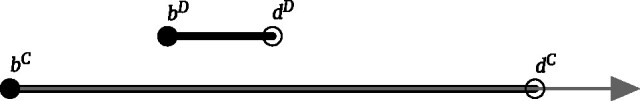
Case 2.

Case 2:  $b_i^C \le b_i^D \le d_i^D \le d_i^C$ (Fig. 3).

\begin{eqnarray*}
\left \| h_i(t)\right \| &= \int _{b_i^C}^{b_i^D}{\left | 1\right | dt} + \int _{b_i^D}^{d_i^D}{\left | 1-1\right | dt } + \int _{d_i^D}^{d_i^C}{\left | 1\right | dt} \\ &= \left | b_i^D - b_i^C\right | + \left | d_i^C - d_i^D\right | \\ &\le 2 \max (\left | b_i^D - b_i^C\right | , \left | d_i^D - d_i^C\right | ) . \end{eqnarray*}

The matching of one point $(b_i^C, d_i^C) \in C$ with a point in the diagonal of *D* is a degenerate case 2 with $b_i^C \le b_i^D = d_i^D \le d_i^C$. Note that, because of this, *C* and *D* are not required to have the same number of off-diagonal points.

**Figure 4:**
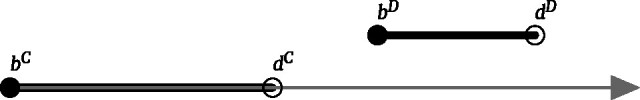
Case 3.

Case 3:  $b_i^C \le d_i^C \le b_i^D \le d_i^D$ (Fig. 4)

This case will never happen as a better matching can always be obtained by matching both points to the diagonal, which is a degenerate case 2.

We have that $\left \| h_i(t)\right \| \le 2\max (\left | b_i^D - b_i^C\right | , \left | d_i^D - d_i^C\right | )$ holds for every *i*. We can then write the difference between 2 Betti curves as

\begin{eqnarray*}
\left \| \beta _k(C, t) - \beta _k(D, t)\right \| _1 &\le \sum _i \left \| I_{[b_i^C,d_i^C)}(t) - I_{[b_i^D,d_i^D)} (t)\right \| _1 \\ &\le \sum _i{2 \max (\left | b_i^D - b_i^C\right | , \left | d_i^D - d_i^C\right | ) } \\ &= 2W_1(C, D) . \end{eqnarray*}

Thanks to the Euler–Poincaré formula, the Euler characteristic curve of a filtered complex *K* can be obtained as the alternating sum of its Betti curves.


\begin{eqnarray*}
ECC(K,t) = \sum _k (-1)^k \beta _k(K,t). \end{eqnarray*}

A stability result for the ECCs can be immediately derived from 1 assuming that the complex *K* has nonzero persistence diagrams in a finite number of dimensions, each of them containing a finite amount of off-diagonal points.

Proposition 2. Let *X* and *Y* be 2 filtered cell complexes. The *L*_1_ difference between the Euler characteristic curves of *X* and *Y* is bounded by the sum of the 1-Wasserstein distances between the corresponding *k*-dimensional persistence diagrams *Dgm*_*k*_(*X*), *Dgm*_*k*_(*Y*).

(2) \begin{eqnarray*}
\left \| ECC(X, t) - ECC(Y, t)\right \| _1 \le \sum _{k} 2 W_1(Dgm_k(X), Dgm_k(Y)) , \end{eqnarray*}

where the sum is over all dimensions in which the persistence diagrams are nonempty.

Proof. It is an immediate consequence of 1 and the triangular inequality.

\begin{eqnarray*}
\left \| ECC(X, t){-}ECC(Y, t)\right \| _1 &=& \left \| \sum _{k=0}^n (-1)^k ( \beta _k(Dgm_k(X), t){-}\beta _k(Dgm_k(Y), t))\right \| _1 \\ && \le \sum _{k=0}^n \left \| \beta _k(Dgm_k(X), t){-}\beta _k(Dgm_k(Y), t)\right \| _1\\ && \le \sum _{k=0}^n 2 W_1(Dgm_k(X), Dgm_k(Y)) . \end{eqnarray*}

The above Proposition 2 is in explicit contrast with the claim that the Euler characteristic curve is unstable. In addition to the already mentioned work by Chung and Lawson [24], a similar statement can be found in [18] and [25].

Remark 3. With reference to Fig. 1, the left-hand side in 2 is finite when the 2 ECCs agree from some filtration value onward. This is exactly what happens, for example, when considering curves obtained from full complexes (i.e., filtered complexes having a single simplex as a last element of a filtration): at some value, all possible faces will have entered the filtration, and so the Euler characteristic will stabilize at 1. If this does not happen, the difference between the 2 ECCs will be unbounded. At the same time, it is straightforward to show that if 2 filtered complexes have different Euler characteristics at +∞, their homologies will have a different number of essential classes. This translates to a different number of points at infinity in the persistence diagrams, whose Wasserstein distance would then be unbounded. In this case, the above result will trivially be +∞ ≤ +∞.

### Euler characteristic profiles

We can immediately extend the notion of *L*_1_ distances between ECCs to work in the general case of *n-*dimensional ECPs.

Definition 19. Let *K*_1_, *K*_2_ be 2 multifiltered cell complexes. The *L*_1_ distance between the corresponding *n-*dimensional Euler characteristic profiles is

\begin{eqnarray*}
||ECP(K_1, v) - ECP(K_2, v)||_1 = \int _{\mathbb {R}^n} |ECP(K_1,v) - ECP(K_2,v)| dv \quad . \end{eqnarray*}

It is natural to ask whether the stability result in 2 can be naturally extended to the multiparameter case. In the existing literature, Chen et al. [19] proposed the following weak *L*_1_-metric in the case of bifiltered complexes (see Definition 3.2 in [19]). Let us remind the proposed construction; consider 2 cell complexes *K*_1_ and *K*_2_ with a bifiltration function $F : K_{1,2} \rightarrow \mathbb {R}^2$. Let us denote with *f* and *g* the 2 real valued functions in the bifiltrations such that *F*(σ) = ((*f*(σ), *g*(σ))) for every cell σ. Moreover, let us index the threshold values of *F* as $I = \lbrace (a_i, b_j) \, | \, 1 \le i \le m , 1 \le j \le n\rbrace$. The idea behind the Chen et al. [19] construction is to fix 1 of the 2 filtrations at a specific value and consider the distances between the single-parameter persistence diagrams induced by the other filtration function. By considering the set of threshold values *I* as a matrix with *i* rows and *j* columns, they define the *i*th *column distance* for the *k-*dimensional PDs [Persistence Diagram (s)] as $D^{i*}_k(K_1, K_2) = W_1 (D^g_k(K_1^{i*}), D^g_k(K_2^{i*}))$. Similarly, the *j*^*th*^  *row distance* is $D^{*j}_k(K_1, K_2) = W_1 (D^f_k(K_1^{*j}), D^f_k(K_2^{*j}))$.

Definition 20 (Definition 3.2 in [19]). The weak *L*_1_ metric between *K*_1_ and *K*_2_ is

\begin{eqnarray*}
D(K_1, K_2) = \max \left \{\sum _{k=0}^M \sum _{i=1}^m D^{i*}_k (K_1, K_2), \: \sum _{k=0}^M \sum _{j=1}^n D^{*j}_k (K_1, K_2) \right \}. \end{eqnarray*}

Being able to recover the single-parameter case, they prove the following stability result.

Proposition 3 (Theorem 3.1 in [19]). Let *K*_1_, *K*_2_ be 2 bifiltered cell complexes. The distance between the corresponding Euler characteristic surfaces is bounded by the weak *L*_1_ metric metric between *K*_1_ and *K*_2_,

\begin{eqnarray*}
||ECP(K_1, v) - ECP(K_2, v)||_1 \le c \cdot D(K_1, K_2) \: , \end{eqnarray*}

for some *c* > 0.

This construction appears to be the natural generalization to multifiltration case of the stability result in 2. However, there are some fundamental problems that undermine the usefulness of such a weak *L*_1_ metric.

Remark 4. In our opinion, the sums over rows or columns in 20 should be replaced with integrals over the filtration ranges. As already discussed in Remark 2, this would allow for more flexibility when dealing with filtration thresholds whose spacing is not constant.

**Figure 5:**
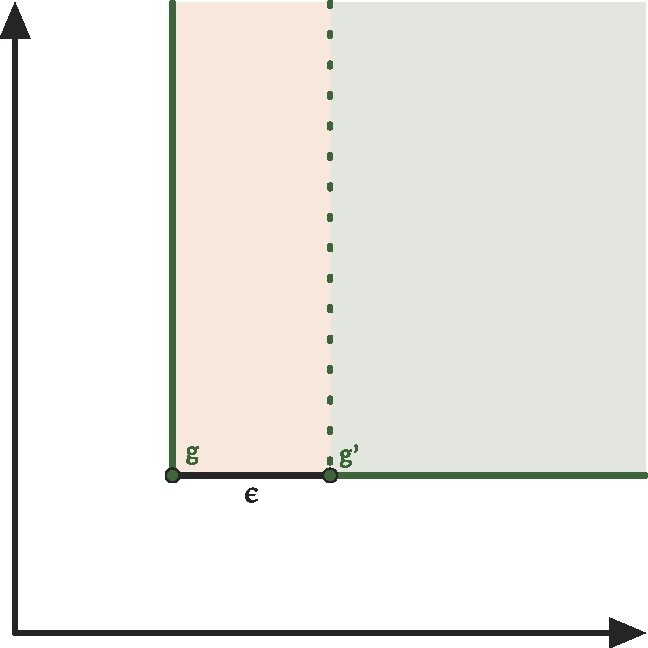
Minimal counterexample for the instability of ECP. Consider a cell complex made by only 1 vertex whose +1 contribution appears at some point $g=(g_1, g_2) \in \mathbb {R}^2$ and move it to *g*′ = (*g*_1_ + ϵ, *g*_2_). Their difference, the region shaded in red, is unbounded.

Remark 5. Proposition 3 will evaluate to a trivial ∞ ≤ ∞ in most cases, even the simplest one. Consider, for example, the situation depicted in Fig. 5 of the ECP of a bifiltered complex *K*_1_ made by just one 0-dimensional cell that appears at filtration value (*g*_1_, *g*_2_). The ECP will then be 1 in the cone $\lbrace (x,y) \in \mathbb {R}^2 : x \ge g_1 , \, y \ge g_2 \rbrace$ and 0 otherwise. We can obtain a different complex *K*_2_ by perturbing the first filtration value by an ϵ amount (*g*_1_ + ϵ, *g*_2_). The difference between the 2 ECPs will then be unbounded. At the same time, also the weak *L*_1_ distance between *K*_1_ and *K*_2_ will be unbounded because in the interval [*g*_1_, *g*_1_ + ϵ) × [*g*_2_, +∞], the 2 complexes have a different number of essential classes and so the *W*_1_ distance between the corresponding PDs will be infinite.

Because of the discussed issues, the stability result in [19], while being formally correct, does not cover a lot of practically relevant cases.

However, in most applications, we can *truncate* the ECP by limiting its filtration domain to the interval [0, *f*_∞_] in every filtration dimension, where *f*_∞_ is a finite value. Note that this value at infinity should not be the same as the maximum filtration value of the complex’s cells, but it should be strictly larger than the maximum filtration value. For example, in the case of images whose pixels have integer filtration values in the [0, 255] range (see section 14 "RGB images" ), we could choose *f*_∞_ = 256 as a truncation value. By doing so, the distance between every pair of ECP will be finite, but it will of course depend on the truncation value. Using truncation, we can state the following result.

Proposition 4. Let *K* be a finite cell complex with an *n*-dimensional multifiltration $F: K \rightarrow \mathbb {R}^n$. We define *K*^ϵ^ as the complex obtained by perturbing the filtration values of each cell in *K* by at most ϵ in *l*^∞^ norm. Let us assume, for simplicity, that we truncate the domain of every filtration function to the same interval [0, *f*_∞_]. We then have the following bound :

\begin{eqnarray*}
||ECP(K, v) - ECP(K^\epsilon , v)||_1 \le |K| \cdot d \cdot \epsilon ^{n-1} \cdot f_{\infty } \quad , \end{eqnarray*}

where |*K*| is the number of cells in the complex and *n* is the number of filtration parameters.

Proof. Let us consider a single-cell σ ∈ *K* with filtration value *g* = (*g*_1_, ⋅⋅⋅, *g*_*d*_). Its contribution to the ECP will be ( − 1)^*dim*(σ)^ in the cone above *g* (i.e., for all points $x \in \mathbb {R}^d$ such that *g* ≤ *x* coordinate-wise). Let σ′ be the corresponding cell in *K*^ϵ^ whose filtration values have been maximally perturbed to *g*′ = (*g*_1_ + ϵ, ⋅⋅⋅, *g*_*d*_ + ϵ). The volume of the region, which is in the cone of *g* but not on the cone of *g*′, can be bounded by a sum of *n n*-dimensional cuboids of base ϵ^*n* − 1^ and height *f*_∞_, each of them corresponding to a shift of ϵ in the direction of one axis, *V*_σ_ ≤ *d* · ϵ^*n* − 1^ · *f*_∞_, where the inequality is due to the fact that cuboids can have a nonempty intersection. One of such cuboids is shaded in red in Fig. 5. Multiplying by the total number of cells gives us the bound.

## Algorithms

Recall that the Euler characteristic of a cell complex is the alternating sum of the number of its cells in each dimension. The contribution of each cell will thus be plus or minus 1 depending on the dimension of the cell. Moreover, this contribution will appear at the cell’s filtration level. Therefore, if we are able to obtain a list of all cells with their filtration values, we can compute the Euler characteristic at each filtration level. This is the main idea behind the following algorithms, which will always return what we will denote as list_of_contributions, a list of pairs (*f*(σ), ( − 1)^*dim*(σ)^) that stores each cell’s contribution to the EC (Euler Characteristic) at the cell filtration level. Once these pairs have been sorted in ascending order with respect to the filtration, the Euler characteristic curve can be reconstructed by progressively summing up the contributions of following elements in the list.

Remark 6. Roune and de Cabezón [26] proved that computing the Euler characteristic of a simplicial complex given by its vertices and facets is #-P-complete. Even if their result does not mention filtered complexes, it follows from it that the problem of computing the ECC is at least P-complete. Otherwise, by contradiction, we could construct an arbitrary filtration of the considered complex and look at the end value of the curve to obtain the Euler characteristic of the complex in polynomial time.

### Vietoris–Rips complexes

In this section, we will present a distributed algorithm to compute the Euler characteristic curve of a Vietoris–Rips simplicial complex obtained from a collection of points in $\mathbb {R}^n$.

Definition 21. Let *X* be a finite collection of points in $\mathbb {R}^n$, also denoted as a pointcloud. Given a parameter ϵ ≤ 0, the *Vietoris–Rips complex* constructed from *X* is the collection of all subsets of the diameter at most 2ϵ, where the diameter is the greatest distance between any pair of vertices

\begin{eqnarray*}
\text{V-R}(X, \epsilon ) = \lbrace \sigma \subseteq X \mid diam(\sigma ) \le 2\epsilon \rbrace \quad . \end{eqnarray*}

The filtration of each simplex is given by its diameter.

The Vietoris–Rips complex is a *flag complex*; this means that a subset *S* of vertices is in the complex if every pair of vertices in *S* is in the complex. This is analogous to saying that the Vietoris–Rips complex is completely determined by its 1-skeleton graph as there is a 1-to-1 correspondence between simplices in the complex and cliques in its 1-skeleton graph.

Therefore, it is straightforward to see that listing all the simplices in a Vietoris–Rips complex is equivalent to performing a cliques count of its 1-skeleton graph [27]. In order to compute the contributions to the ECC, we need to find an efficient and distributed way to list all cells in the simplex (i.e., all cliques in the 1-skeleton graph) and their filtration values (i.e., the length of the longest edge in each clique); this can be achieved in the following way. Given an ordered list of points *X* = {*x*_*i*_: *i* ∈ [1, *n*]} (the points can be ordered in an arbitrary way) and a maximum distance ϵ, for each point *x*_*i*_, we build its local graph *G*_*i*_ of *subsequent neighbors*—namely, all points *x*_*j*_ ∈ *B*(*x*_*i*_, ϵ)∩*X* with *j* > *i*. For each *G*_*i*_, we list all of its cliques that contain *x*_*i*_. They will correspond to simplices with *x*_*i*_ being the smallest vertex in the chosen ordering of points. This way, each simplex σ in the V-R complex will be generated exactly once, when considering the local graph of its lowest vertex in the considered ordering.

Algorithm 1, which uses this idea, describes a way to list all the simplices of increasing dimension. At each iteration, we obtain a list of *d*-dimensional simplices (given as collections of vertices) and, for each of them, a list of common subsequent neighbors of its vertices. We can then extend each simplex to a (*d* + 1)–dimensional one by adding 1 common neighbor to the collections of vertices. When doing so, we need to update the simplex’s filtration value if one of the newly added edges is longer than the current filtration. Moreover, we need to update the list of common subsequent neighbors by intersecting it with the subsequent neighbors of the newly added vertex. Once we have obtained all possible (*d* + 1)–simplices, we carry out this extension procedure 1 dimension higher. All of these operations are performed at the local graph of each vertex. The procedure ends when no simplex can be extended (i.e., when all maximal simplices have been listed). This construction might be understood as a breadth-first traversal of the simplex tree [27].

**Algorithm 1:** COMPUTE LOCAL CONTRIBUTIONS V-R

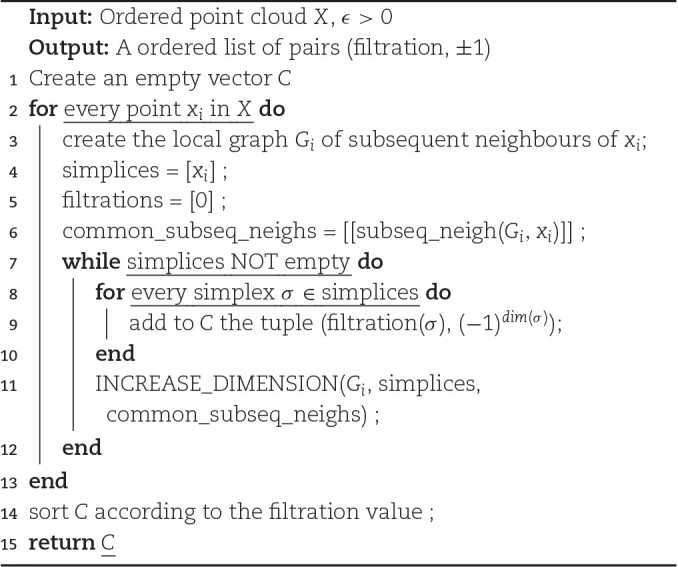

The main advantages of the proposed algorithm are 2: it does not require to construct the whole complex, leading to a significant decrease in memory utilization, and it considers each point separately, allowing the computations to be carried out independently.

The inputs of our algorithm are *X*, a ordered list of points in $\mathbb {R}^n$ and a maximum filtration value ϵ. The output is list_of_contributions, an ordered list of pairs. For each simplex σ, we store its contribution as a tuple (*f*(σ), ( − 1)^*dim*(σ)^). The output list will sorted according to the filtration values.

Note that the Algorithm 1 is correct. First, every simplex in the Vietoris–Rips complex will be generated. It will happen when its smallest vertex in the considered order will be considered in the *for* loop. Second, each simplex will be generated only once in the *INCREASE_DIMENSION* procedure (Algorithm 2). A simplex σ = [*v*_0_, …, *n*_*n* − 1_, *v*_*n*_], where *v*_0_ < … < *n*_*n* − 1_ < *v*_*n*_, will be generated from a simplex [*v*_0_, …, *n*_*n* − 1_] by adding *v*_*n*_ as a common neighbor of its vertices.

**Algorithm 2:** INCREASE_DIMENSION

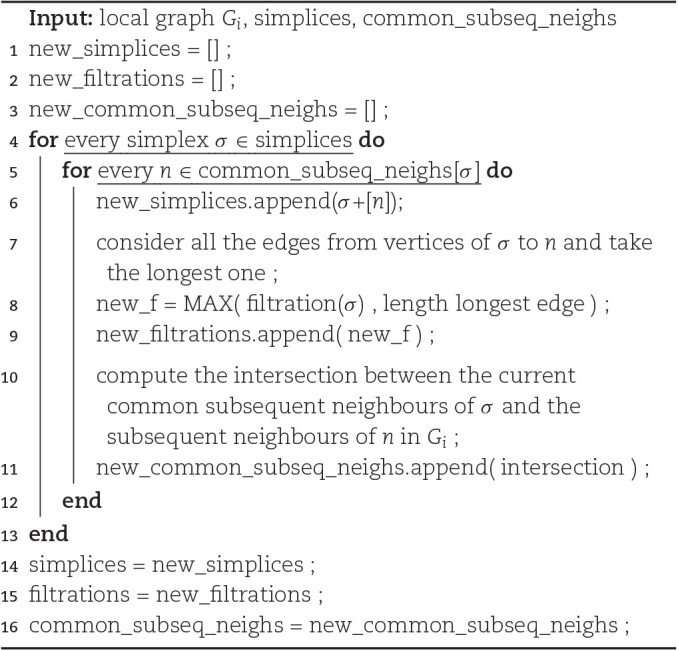

### Time performance

The worst-case scenario occurs when the the 1-skeleton graph is fully connected. Assuming the pointcloud consists of *n* points, the resulting V-R complex will contain 2^*n*^ − 1 simplices. In this case, the time complexity of Algorithm 1 is $\mathcal {O}(2^{n - 1} n)$. More details are provided in Appendix A.

### Memory performance

Assuming the worst-case scenario, the size of the output list of contributions is *O*(*n*^2^) while the maximal memory required at 1 intermediate step is $O(2^n / \sqrt{n})$. More details are provided in Appendix A.

### Choice of the vertex ordering

**Figure 6:**
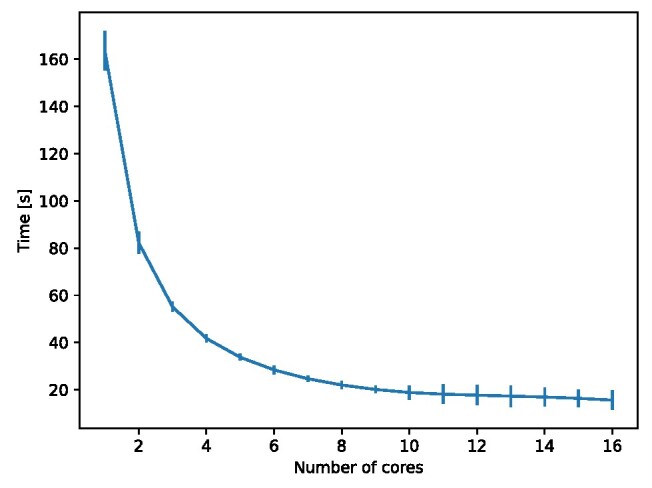
Average runtime over 10 runs of Algorithm 1 as a function of the number of cores used. Contributions computed for the V-R complex obtained from 10,000 points sampled from the unit 4-sphere up to a maximum radius of 0.4. Experiment run on a AMD Ryzen Threadripper PRO 5955WX CPUu. Error bars are scaled up by a factor of 20 for visibility.

**Figure 7:**
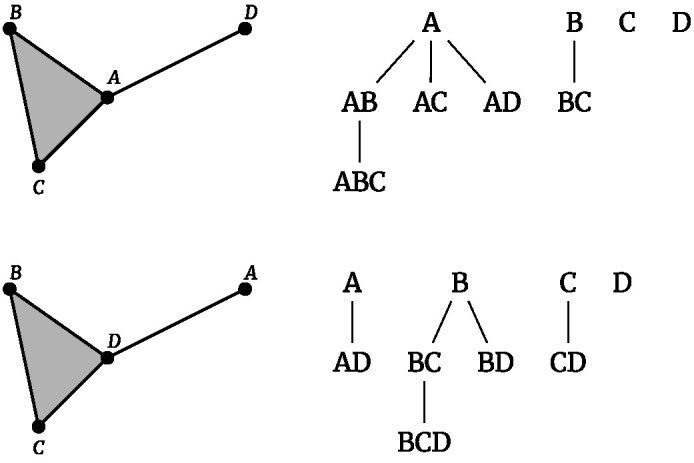
Different ordering of the vertices can produce different simplex trees. In the first row, vertices are ordered by decreasing number of neighbors and in the second row by increasing number. The second choice produces more evenly sized trees.

**Figure 8:**
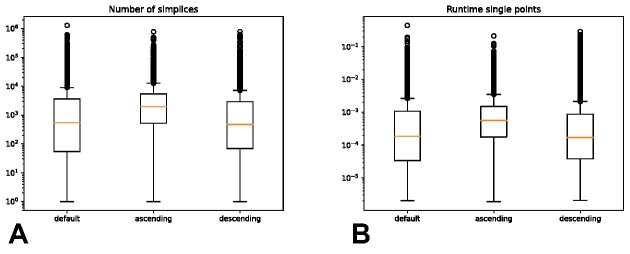
Effect of different orderings of vertices for the example in Fig. 6. Selecting the ascending order allows achieving a more even distribution in the number of simplices in each tree (A), thus reducing the number of very large trees that dominate the running time (B).

Note that the total running time of the fully parallelized Algorithm 1 can be dominated by few vertices whose simplex tree is considerably larger than the others. This explains the plateau in Fig. 6. This effect can be mitigated by choosing a different ordering of the vertices. One efficient choice is to order the vertices by increasing number of ϵ-neighbors. Since the local graph for each vertex is constructed by considering only its subsequent neighbors, this ordering will produce more evenly sized simplex trees. A simple example is shown in Fig. 7, while the effect of this reshuffling on a larger dataset is shown in Fig. 8.

### Cubical complexes

Cubical complexes are the most used combinatorial structure to represent digital grayscale images and extract topological information from them. There are 2 ways to construct a cubical complex from an image, the V-construction and the T-construction. The former identifies pixels—also know as voxels, in case of images of arbitrary dimension—with the vertices (the 0-dimensional cells) of the cubical complex. Voxels’ values are used to define the filtration on the vertices and the filtration of each other elementary cube is the maximal value of its vertices. The T-construction can be seen as the dual procedure, voxels’ values are assigned to the top-dimensional cubes, and the filtration values are propagated to lower-dimensional cells by taking the minimum over the cofaces. The relation between these 2 constructions is explored in a recent work by Bleile et al. [28]. In this article, we chose the T-construction, although the presented techniques translate easily to the V-construction.

**Figure 9:**
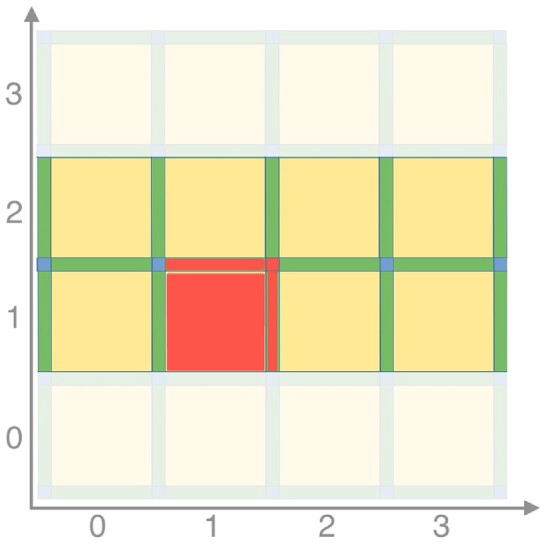
A slice of a cubical complex obtained from a 2-dimensional image. The image’s pixels are associated to the top-dimensional cells, depicted in yellow. Algorithm 3 takes as input a 2-voxel tick slice of the image and iterates through the voxels in the bottom row. At each iteration, a voxel is selected and the contributions of the cells in its upper closure are computed. In this example, the voxel at coordinates (1, 1) is selected, and the considered contributions are depicted in red: the one coming from the corresponding 2-cell, the two from the 1-cell shared with (2,1) and (1,2), and the contribution from the 0-cell shared with (2,1), (1,2), and (2,2).

Similar to the V-R case, we are interested, given a grayscale *n*-dimensional image, in obtaining a list of contributions to the Euler characteristic of its corresponding cubical complex. As before, we then need to iterate over all cells σ in the complex and store each contribution as a tuple (*f*(σ), ( − 1)^*dim*(σ)^). This can be achieved in a streaming fashion by loading into memory a 2-voxel-high slice of the image, iterating through the cells in the bottom row computing their contributions, and then moving the sliding window up by 1 voxel. To make sure we consider each cell’s contribution exactly once, at each iteration, we consider 1 voxel and compute the contributions to the Euler characteristic of the cells in its *upper closure*. Assuming that we can identify each top-dimensional cell *c*_*i*_ with the indices (*x*_1_, ⋅⋅⋅, *x*_*n*_) of the corresponding voxel in the input *n*-dimensional image, we define the upper closure of *c*_*i*_ as the set containing *c*_*i*_ and all its faces that are shared with other top-dimensional cells *c*_*j*_ whose indices are *y*_*i*_ = *x*_*i*_ or *y*_*i*_ = *x*_*i*_ + 1 for all *i*. An example of this procedure can be found in Fig. 9.

As already mentioned, a similar streaming algorithm to compute the ECC of grayscale images has been presented by Heiss and Wagner [15]. They also provide a fast open-source C++ implementation. Recently, Wang et al. [16] provided a GPU implementation of the same algorithm. However, there is a significant difference between their approach and the one we describe in Algorithm 3: they keep track of the faces introduced by each voxel by looking at the gray values of the voxel’s 3^*d*^ − 1 neighbor and store the *cumulative* change in the EC at the voxel’s filtration value. This requires sorting the voxel’s value from the lowest to highest. While such a sorting can be done for 1-value filtrations, it cannot be performed for multifiltrations, as no good ordering exists in the general case. There are some small differences in the implementation too: CHUNKYEuler only works with integer filtration values and only accepts “raw” binary files as input. Our implementation, while being not as fast as CHUNKYEuler, offers the user more flexibility in the input and choice of filtration (or multifiltration) values.

**Algorithm 3:** COMPUTE LOCAL CONTRIBUTIONS CUBICAL

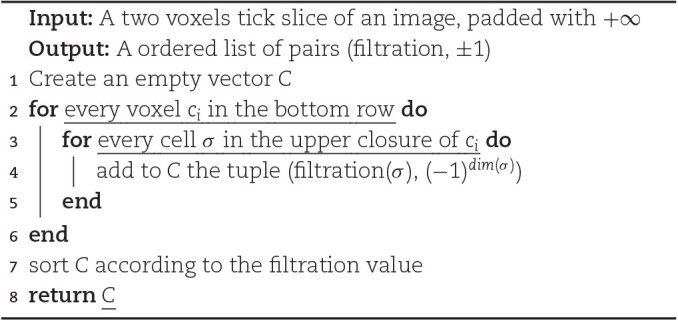

### Time and memory complexity

Considering a *d*-dimensional image with *n* voxels as input, the resulting cubical complex will have 3^*d*^*n* cells. The running time of Algorithm 3 is then linear in the number of cells in the complex with a multiplicative constant, which is exponential in the dimension. This is not a problem in practice as images with dimension larger than 3 are not common in applications. The memory requirement is just the space needed to store a 2-row slice of the input image, and the memory overhead for computing the local contributions for each voxel is negligible.

### From Euler characteristic curves to profiles

Both Algorithm 1 and Algorithm 3 can be immediately extended to compute the Euler characteristic profile of multifiltered Vietoris–Rips or cubical complexes. In the Vietoris–Rips case, we require that all filtration functions should be defined on the vertices or the edges and then be extended to higher-dimensional simplices by some user-defined rule. This is to enssure that the resulting multifiltered V-R complex is still a flag complex. In the case of cubical complexes, we assume that the input images contain a *n*-tuple of numbers in each voxel—RGB images are a typical *n* = 3 example—and values are propagated to lower-dimensional cells by some user-defined rules. In both cases, the output of both algorithms will be a list of (*n* + 1)−tuples (*f*_1_(σ), ⋅⋅⋅, *f*_*n*_(σ), ( − 1)^*dim*(σ)^) that stores the list of contributions to the ECP at different points $f(\sigma ) \in \mathbb {R}^n$.

Remark 7. In above, the simplest case of so-called 1-critical multifiltration is discussed. In this case, each cell σ appears in a unique value of the multifiltration. In a general case, a cell σ may appear in multiple noncomparable values *p*_1_, …, *p*_*k*_ of multifiltration. A simple generalization described below allows to adopt this presented algorithm to the general case; let us assume that each *p*_*i*_ is *n*-dimensional tuple, $p_i = (p_i^0,p_i^1,\ldots ,p_i^n)$. We assume that *p*_*i*_ and *p*_*j*_ are not comparable provided *i* ≠ *j*. It means that there exist a pair of coordinates *l* ≠ *m* so that $p_i^l < p_j^l$ and $p_i^m > p_j^m$. Then, the cell σ contributes the value $(-1)^{\dim (\sigma )}$ for all the points $x \in \mathbb {R}^n$ for which there exist *i* such that *x* > *p*_*i*_. Note that the regions consisting of points greater that *p*_*i*_ overlap for different *i* ∈ {1, …, *k*}; hence, we need to avoid double and multiple counting of the contributions. Below we describe a procedure to achieve it and enforce the contribution of exactly $(-1)^{\dim (\sigma )}$ for all *x* > *p*_*i*_ for arbitrary *i* ∈ {1, …, *k*}. For that purpose, given *i* ≠ *j*, we define $p_i \vee p_j = ( \max (p_i^1,p_j^1),\max (p_i^2,p_j^2),\ldots ,\max (p_i^n,p_j^n) )$. Algorithm 4 defines a set of points with appropriate contributions to enforce the required condition for all *x* ≥ *p*_*i*_ for all *i* ∈ {1, …, *k*}.

It is straightforward to see that for any given cell σ, its contributions to the ECP will change at most in *p*_*i*_∨*p*_*j*_ for *i, j* ∈ {1, …, *k*}, where {*p*_1_, …, *p*_*k*_} are incompatible points in which σ appears in the multifiltration. Algorithm 4 scans all those points and assigns the appropriate value (see lines 1 and 9) to contributions to the ECP. Note that all points *p*_1_, …, *p*_*k*_ have their contributions initially set in line 1. Consequently, the presented algorithm will terminate, as in each iteration, at least 1 *p* will be added to the *Contribution* list. In addition, it explicitly enforces the correct contribution of the cell σ to all points *x* ≥ *p*_*i*_ for any *i* ∈ {1, …, *k*}.

**Algorithm 4:** CONTIBUTION OF σ TO ECP

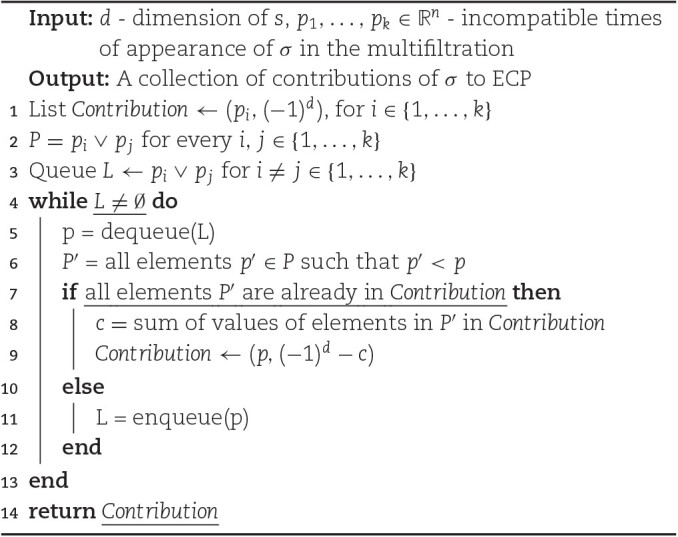

## Data Structures for ECPs

All the algorithms we described in the previous section output a list of contributions to the Euler characteristic profile. For an *n*-dimensional profile, each contribution in the list is a pair where the first entry is an *n*-tuple storing the coordinates in $\mathbb {R}^n$ at which the Euler characteristic varies by the integer values stored in the second item. When dealing with 1-dimensional ECCs, it makes sense to sort the contributions according to their filtration value, in order to perform faster operations on them.

### Retrieving the EC at some filtration values

Given an ECP as a list of contributions, the first basic operation is to retrieve the value of the Euler characteristic at an arbitrary filtration value f_*_. It can be obtained by summing up all the contributions in the ECP that appear at filtration values less than or equal to f_*_. For a *d*-dimensional ECP, this can be achieved in linear time with respect to the size of the contribution list. In the 1-dimensional case, we can take advantage of the total ordering on the list of contributions, since the filtration values $f_i \in \mathbb {R}$. By doing so we can build an auxiliary data structure storing the value of the Euler characteristic at each *f*_*i*_, the points in which the ECC is changing value. This can be done in *O*(*n*) time and space, where *n* is the length of the list of contributions. Given such a structure, computing the value of the ECC at a given filtration f_*_ boils down to the the search for the largest jump point *f*_*i*_ < f_*_ and retrieving the value of the ECC therein. This can be achieved by interpolation search in *O*(*log*(*log*(*n*))) time.

### Computing distances

#### Distances between Euler characteristic curves

In the Euler characteristic curves section, we introduced the notion of difference between 2 ECCs, expressed in terms of the *L*_1_ norm of the difference between the 2 curves. One should note that, in the case of finite Vietoris–Rips or cubical complexes, such a difference is always finite (but not bounded) as all ECCs will eventually stabilize to 1 for a sufficiently large filtration value. When the construction of a Vietoris–Rips complex is stopped at a certain diameter 2ϵ, and the final complexes have more than 1 infinite homology, it make sense to restrict the integral used in distance computations to an interval [0, 2ϵ] in order to make the distances between the ECCs finite.

Both Algorithms 1 and 3 return the computed ECC as a list of pairs (*f*_*i*_, *c*_*i*_) where *c*_*i*_ is an integer representing the change in the Euler characteristic at filtration *f*_*i*_. Such a list is sorted in increasing order with respect to the filtration values. Using such a data structure, the difference between 2 ECCs can be computed in linear time with the size of the lists. Given 2 list of contributions *ECC*_1_ and *ECC*_2_, we can merge them in linear time, preserving the order. While merging, we flip the sign of all the contributions coming from *ECC*_2_. Let us denote the obtained list with *ECC*_1−2_. Now the difference can be computed by iterating over the full list


\begin{eqnarray*}
||ECC_1 - ECC_2||_1 = \sum _i (f_{i+1} - f_i) EC(f_i) \quad , \end{eqnarray*}

where $EC(f_i) = \sum _{j=0}^i c_j$ with respect to the ordering of *ECC*_1−2_.

#### Distances between Euler characteristic profiles

Unfortunately, the strategy proposed in the previous section is difficult to generalize in the multifiltration setting as there is no natural way to sort the list of contributions. We present here a basic algorithm to compute the distances between 2 ECPs and leave the search for a potentially faster algorithm to future work.

**Figure 10:**
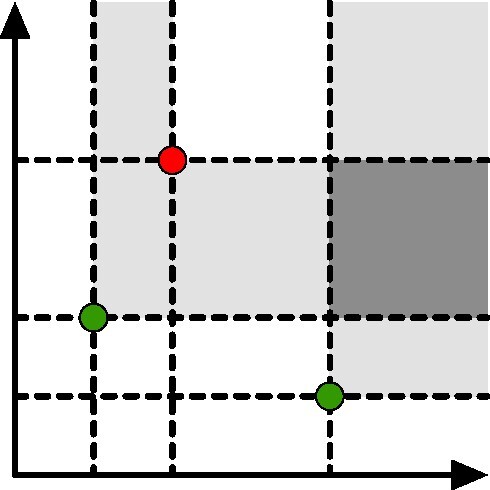
Example of a 2-dimensional ECP with 3 contributions. The green points indicate a +1 while the red point is a −1. The plane can then be subdivided in a 4 × 4 irregular grid. The coloring of each block indicates the value of the EC in that region; white is 0, light gray is 1, and dark gray is 2.

Let *ECP*_1_ and *ECP*_2_ be 2 lists of contributions representing 2 *n*-dimensional profiles. We can merge them in linear time, as in the 1-dimensional case, flipping the sign of the contributions in the second list. Let *N* be the total number of contributions. With reference to Fig. 10, the coordinates of such contributions will create an *n*-dimensional irregular grid of size (*N* + 1). The value of the EC inside each cuboid will be equal to the EC at the cuboid’s bottom left corner and can be computed in *O*(*N*). The *L*_1_ distance between the 2 ECPs can then be obtained by summing up the values of the EC in each cuboid weighted by the cuboid’s volume. Given that the number of cuboids is (*N* + 1)^*d*^, this operation can be computed in *O*(*N*^*d* + 1^). Note that the ECPs need to be truncated in order to avoid cuboids with infinite volume.

## Vectorization

Vectorizing the ECC/ECP is a critical step if we are interested in using these invariants in a machine learning framework.

### Curves

Assume we are given an ECC whose filtration values ranges from 0 to *f*_*max*_. We can convert it to a vector by evenly sampling it *N* times between 0 and *f*_*max*_. If we choose to include the endpoints, the resulting vector will be *vec*(*ECC, N*) = [*EC*(0), *EC*(Δ), *EC*(2Δ), ⋅⋅⋅, *EC*((*N* − 2)Δ), *EC*(*f*_*max*_)], where Δ is the vectorization’s resolution, which is defined as Δ = *f*_*max*_/(*N* − 1).

**Figure 11:**
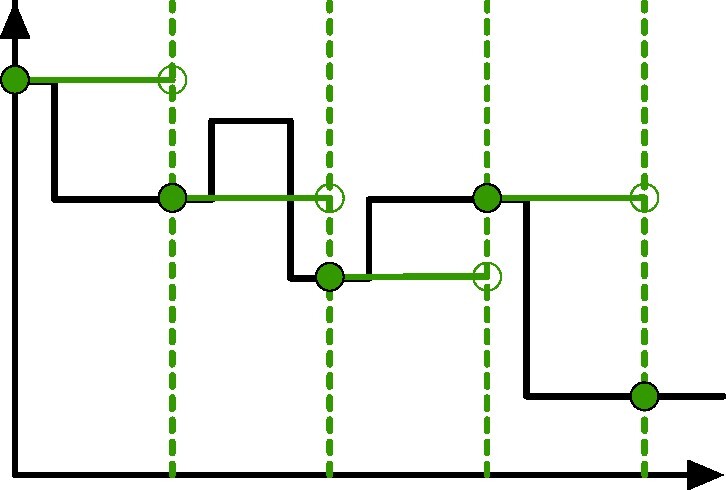
An Euler characteristic curve (black) and its vectorized version (green) with resolution Δ. In this case, the vectorized version is stored as a vector of length 5 (the green filled-in points) but can be reconverted to a step-size function.

The vectorized ECC can be obtained by such vector as the union of *N* − 1 left-closed, right-open intervals of length Δ that correspond to sampling the value of the EC at filtration value *f*_*i*_ and extending it until *f*_*i* + 1_. It makes sense then to ask whether it is possible to bound the difference between an ECC and its vectorized representation. Fig. 11 is an example of such difference when a curve is sampled in 5 points.

Proposition 5. Let *K* be a filtered cell complex whose filtration values ranges from 0 to *f*_*max*_. The *L*_1_ norm between the Euler characteristic curve of *K* and its vectorized version at resolution Δ is bounded by

(3) \begin{eqnarray*}
|| ECC(K) - vec(ECC(K), N)||_1 \le \Delta ( |K|/2 + F ) \end{eqnarray*}

where |*K*| is the number of simplices in the complex and $F = \sum _{i=0}^{n-2}|EC(i \Delta ) - EC((i+1)\Delta )|$ is the sum of the absolute value of the differences between consecutive values in the vectorized Euler characteristic *vec*(*ECC*(*K*), *N*).

Proof. We will prove the 2 terms in the bound separately as they come from 2 different types of errors.

**Figure 12:**
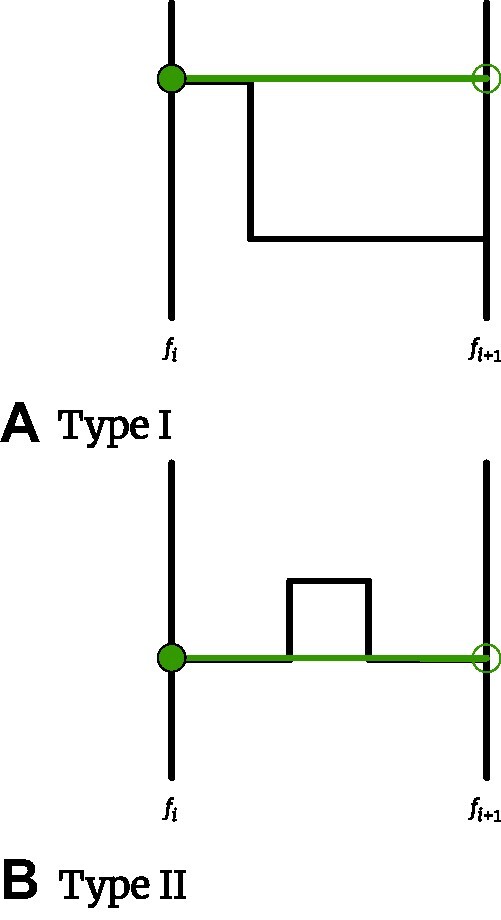
The 2 possible source of errors during vectorization of an ECC.

Type I errors occur when the EC at 2 consecutive sampling points *f*_*i*_ and *f*_*i* + 1_ is different. The simplest case is depicted in Fig. 12 A; the EC changes values in between the sampling interval. We can upper bound this error with the area of the rectangle having as base the vectorization’s resolution Δ = *f*_*i* + 1_ − *f*_*i*_ and as height the difference between the EC at the 2 sampling points |*EC*(*f*_*i*_) − *EC*(*f*_*i* + 1_). Note that this bound also holds in the more general case where the EC varies monotonically at multiple values inside the sampling interval. By summing up all the contributions, we obtain the value $\Delta \cdot F = \Delta \cdot \sum _{i=0}^{n-2}|EC(i \Delta ) - EC((i+1)\Delta )|$.

Type II errors (see Fig. 12B) occur when the EC has the same value at consecutive filtration steps but varies in between. The maximum possible variation can be upper bounded by the area of the rectangle with Δ as base and half the number of cells in the complex as height. Each cell contributes to the EC by ±1, and the factor one-half is due to the constraint that the EC has the same value in *f*_*i*_ and *f*_*i* + 1_. This amounts to the values Δ · |*K*|/2.

By summing up the 2 contributions, we obtain the bound in 3. Note that a generic situation can always be described as a combination of type I and type II errors.

**Figure 13:**
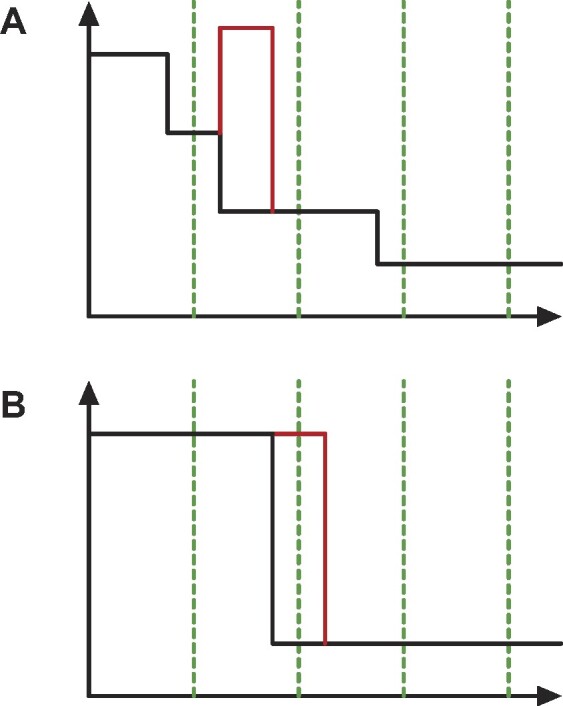
Two ECCs superimposed in the same plot. (A) The 2 curves can be made arbitrarily far apart in *L*_1_ but have the same vectorization. (B) The 2 curves can be made arbitrarily close but have drastically different vectorizations.

We have shown a way to bound the distance between an ECC and its vectorized version. Another possible stability question is whether this vectorization preserves distances between ECCs. In other words, we are interested in knowing whether something can be said for ||*vec*(*ECC*_1_, *N*) − *vec*(*ECC*_2_, *N*)|| given ||*ECC*_1_ − *ECC*_2_||. Unfortunately, it is possible to construct examples in which 2 curves can be made arbitrarily far apart, but they have the same vectorization or 2 curves can be made arbitrarily close but have drastically different vectorizations. Fig. 13 shows 2 such examples. Moreover, in the existing literature, Johnson and Jung [29] prove that the distance between 2 vectorized Betti curves cannot be bounded by the Wasserstein distance between the respective persistence diagrams. They propose a stable vectorization inspired by Gaussian smoothing techniques.

### Profiles

**Figure 14:**
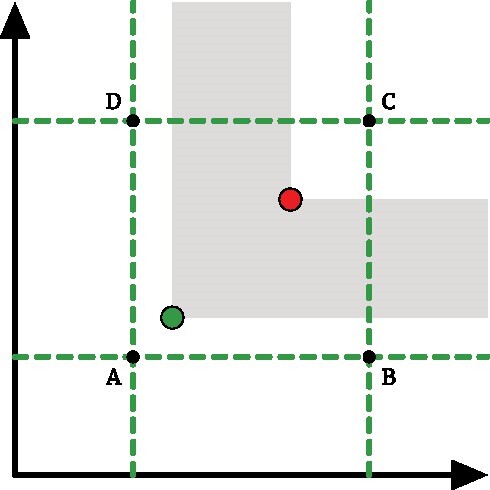
A 2-dimensional analogue of a type II error of Fig. 12. The ECP is vectorized by sampling the EC values on the green grid. We can add pair of cells with contributions ±1 inside the rectangle ABCD in such a way that the value of the EC on the vertices does not change. However, such contributions have a nonzero sum on an area that can be made arbitrarily large.

An *n*-dimensional Euler characteristic profile whose filtration values ranges from 0 to $f^i_{max}$ for *i* ∈ 1⋅⋅⋅*n* can be vectorized in a similar fashion by sampling it on a grid of size *N*_1_ × *N*_2_ × ⋅⋅⋅ × *N*_*n*_. In general, the *N*_*i*_ can be different and thus lead to different resolutions Δ_*i*_ on the various filtration parameters. The output of this sampling procedure is an *n*-dimensional tensor *vec*(*ECP, N*_*i*_) that can be eventually flattened to a 1-dimensional vector. Although this is an intuitive generalization of the 1-dimensional ECC case, the procedure has an increased computational cost due to the difficulties in sampling EC values from a profile, as already discussed in section 14 "Retrieving the EC at some filtration values" . Moreover, the stability result in 5 cannot be generalized to the multiparameter setting. As depicted in Fig. 14, the grid vectorization could not detect the contributions coming from pairs of cells. In the multiparameter case, however, it is not possible to bound this contribution using only the vectorization resolutions Δ as such a contribution can persist on subsequent grid elements up to infinity.

## Examples and Experiments

### RGB images

A toy experiment using 3-dimensional Euler characteristic profiles can be constructed using RGB images. In a RGB image, each pixel contains a tuple of 3 integers, each ranging from 0 to 255. They stand for the red, green, and blue color channel, and all colors in the visible spectrum can be represented by a 3-tuple. In particular, black is coded by (0,0,0) and white is (255, 255, 255).

**Figure 15:**
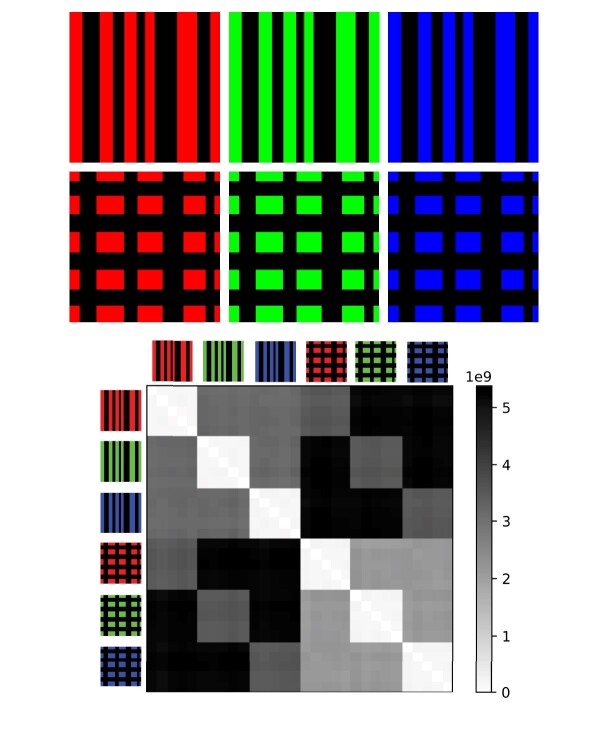
A 60 × 60 distance matrix between Euler characteristic profiles of different RGB images.

In this example, we consider 2 different textures, stripes and checks; each of them can be red, green, or blue. We generate 10 samples of each combination of style and color by adding random Gaussian noise to each pixel. We then compute the 3-dimensional Euler characteristic profile of the cubical complex obtained from each image and compute the matrix of pairwise *L*_1_ distances between them. Such a matrix is show in Fig. 15. It confirms that distance between Euler characteristic profiles of different images increase following the intuitive sequence same style, same color < same style, different color < different style, same color < different style, different color.

**Figure 16:**
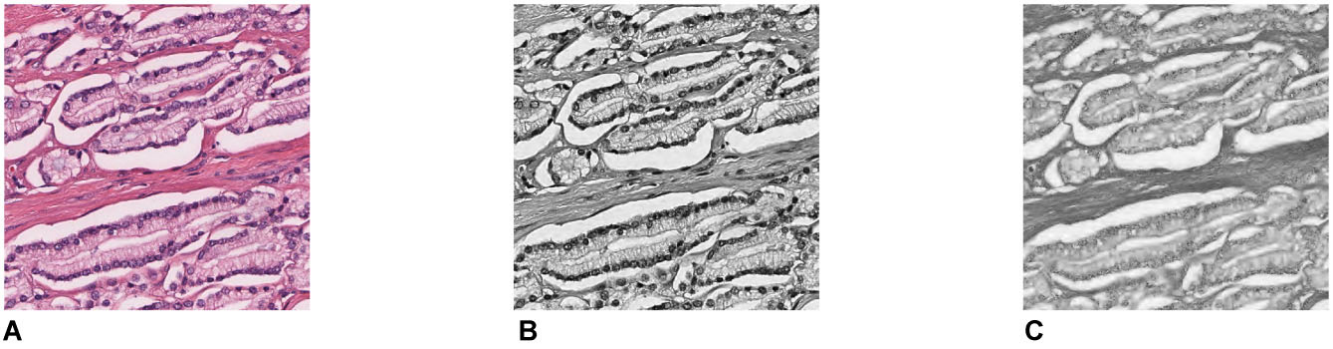
(A) A raw RGB ROI. (B) Hematoxylin channel. (C) Eosin channel.

**Figure 17:**
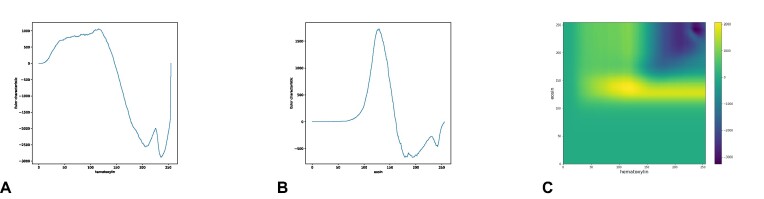
(A) Hematoxylin ECC for the ROI in Fig. 16. (B) The eosin ECC. (C) The combined ECP.

### Immune cell spatial patterns in tumors

Vipond et al. [30] applied multiparameter persistent homology (MPH) landscapes to study immune cell location in digital histology images from head and neck cancer. They extracted the locations of 3 immune cell types from histology slides, thus obtaining a list of pointclouds labeled CD8^+^, FoxP3^+^, or CD68. The goal is to correctly classify a pointcloud. All pointcloud data are available at [31]. The authors created a bifiltered Vietoris–Rips complex from each pointcloud, using radius and a codensity function defined over each vertex *p* as $\rho _{10}(p) = \frac{1}{10}\sum _{i=1}^{10} ||p - p_i||_2$, where *p*_*i*_ is the *i*th nearest neighbor of *p*. They then computed MPH landscapes and used them as input for 1 of 3 classifiers: linear discriminant analysis (LDA), regularized linear discriminant analysis (rLDA), and regularized quadratic discriminant analysis (rQDA) [32]. They made a randomized 80/20 training/test split and evaluated the classification accuracy of 3 classifiers on the test data for each pair of cell types and for the 3-class problem. The classification results are reported in the supplementary material of [30].

We used the authors’ code to regenerate the same standard Vietoris–Rips and bifiltered Vietoris–Rips complexes from the provided pointclouds. We then computed ECC (radius only) and ECP (radius and codensity) for each complex and used them as input for the same LDA, rLDA, and rQDA classifiers using the same train–test split procedure. The average accuracy for the various classification tasks is reported in Tables 1, 2, and 3. Both ECC and ECP significantly outperform MPH landscapes while there is apparently no gain in moving from ECC to ECP. This can be an indication that the second dimension in the filtration (the codensity parameter) does not contain significant information.

**Table 1:** Average classification accuracy for the LDA classifier using as input MLP (Multiparameter Persistence Landscape), ECC, or ECP. Data for each tumor are split into 80/20 train–test splits and classification accuracy is reported as the mean over 100 repetitions of splitting, training, and testing.

	CD68^+^ vs. FoxP3^+^	CD8^+^ vs. FoxP3^+^	CD8^+^ vs. CD68^+^	CD8^+^ vs. CD68^+^ vs. FoxP3^+^
	MPL - ECC - ECP	MPL - ECC - ECP	MPL - ECC - ECP	MPL - ECC - ECP
T_A	0.584 - 0.938 - **0.941**	0.672 - **0.994** - 0.988	0.669 - **0.894** - 0.856	0.486 - **0.896** - 0.886
T_B	0.794 - 0.917 - **0.922**	0.88 - **0.992** - **0.992**	0.54 - 0.943 - **0.962**	0.568 - 0.921 - **0.940**
T_C	0.723 - **0.947** - 0.904	0.7 - **0.884** - 0.859	0.605 - **0.811** - 0.699	0.505 - **0.842** - 0.755
T_D	0.811 - **0.960** - 0.933	0.899 - **0.986** - 0.985	0.644 - 0.802 - **0.807**	0.613 - 0.862 - **0.874**
T_E	0.732 - **0.941** - 0.940	0.644 - 0.867 - **0.869**	0.593 - **0.806** - 0.688	0.511 - **0.842** - 0.719
T_F	0.738 - 0.655 - **0.933**	0.644 - 0.619 - **0.830**	0.73 - 0.709 - **0.850**	0.511 - 0.578 - **0.824**
T_G	0.771 - 0.788 - **0.858**	0.782 - 0.791 - **0.904**	**0.675** - 0.614 - 0.609	0.599 - **0.673** - 0.659
T_H	0.710 - 0.651 - **0.885**	0.682 - 0.747 - **0.955**	0.628 - 0.695 - **0.891**	0.555 - 0.659 - **0.845**
T_I	0.733 - **0.788** - 0.737	**0.758** - 0.716 - 0.679	0.540 - 0.693 - **0.713**	0.548 - **0.716** - 0.493
T_J	0.727 - 0.642 - **0.767**	0.535 - 0.678 - **0.857**	0.602 - 0.808 - **0.868**	0.449 - 0.507 - **0.699**
T_K	0.510 - **0.872** - 0.770	0.570 - 0.784 - **0.816**	0.502 - 0.823 - **0.877**	0.404 - 0.594 - **0.635**
T_N	0.493 - 0.457 - **0.570**	0.512 - **0.658** - 0.632	0.577 - 0.507 - **0.760**	0.342 - **0.462** - 0.370
T_O	**0.948** - 0.830 - 0.840	**0.788** - 0.602 - 0.754	0.532 - 0.484 - **0.598**	0.550 - 0.431 - **0.615**

**Table 2:** Average classification accuracy for the rLDA classifier using as input MLP, ECC, or ECP. Data for each tumor are split into 80/20 train–test splits and classification accuracy is reported as the mean over 100 repetitions of splitting, training, and testing.

	CD68^+^ vs. FoxP3^+^	CD8^+^ vs. FoxP3^+^	CD8^+^ vs. CD68^+^	CD8^+^ vs. CD68^+^ vs. FoxP3^+^
	MPL - ECC - ECP	MPL - ECC - ECP	MPL - ECC - ECP	MPL - ECC - ECP
T_A	0.491 - **0.967** - 0.964	0.642 - **0.973** - 0.967	0.630 - **0.840** - 0.830	0.427 - 0.858 - **0.859**
T_B	0.760 - **0.892** - 0.869	0.787 - **0.986** - 0.985	0.671 - 0.942 - **0.945**	0.604 - **0.868** - 0.865
T_C	0.863 - **0.906** - 0.896	0.747 - **0.847** - 0.842	**0.653** - 0.584 - 0.614	**0.640** - 0.628 - 0.627
T_D	0.683 - **0.926** - 0.918	0.829 - **0.990** - 0.988	0.476 - **0.779** - **0.779**	0.492 - **0.779** - 0.775
T_E	0.820 - **0.886** - 0.883	0.736 - **0.929** - 0.920	0.534 - 0.735 - **0.743**	0.502 - **0.702** - 0.683
T_F	0.623 - 0.899 - **0.925**	0.476 - 0.842 - **0.847**	0.765 - 0.909 - **0.921**	0.408 - 0.845 - **0.847**
T_G	0.886 - **0.932** - 0.927	0.897 - 0.970 - **0.975**	0.446 - **0.696** - 0.692	0.581 - 0.738 - **0.746**
T_H	0.524 - 0.890 - **0.898**	0.735 - **0.930** - 0.929	0.714 - **0.882** - 0.877	0.502 - 0.844 - **0.859**
T_I	0.859 - 0.912 - **0.931**	0.883 - 0.908 - **0.909**	0.484 - 0.470 - **0.474**	0.597 - **0.619** - 0.614
T_J	0.608 - **0.763** - 0.750	0.750 - 0.835 - **0.872**	0.850 - 0.882 - **0.892**	0.536 - 0.653 - **0.670**
T_K	0.376 - **0.868** - 0.804	0.523 - **0.918** - 0.914	0.455 - **0.857** - 0.845	0.261 - **0.718** - 0.679
T_N	0.410 - 0.527 - **0.563**	0.432 - 0.662 - **0.745**	0.643 - 0.690 - **0.713**	0.294 - 0.388 - **0.460**
T_O	0.702 - **0.954** - 0.952	0.644 - **0.806** - 0.772	0.546 - 0.672 - **0.684**	0.429 - **0.639** - 0.632

**Table 3:** Average classification accuracy for the rQDA classifier using as input MLP, ECC, or ECP. Data for each tumor are split into 80/20 train–test splits and classification accuracy is reported as the mean over 100 repetitions of splitting, training, and testing.

	CD68^+^ vs. FoxP3^+^	CD8^+^ vs. FoxP3^+^	CD8^+^ vs. CD68^+^	CD8^+^ vs. CD68^+^ vs. FoxP3^+^
	MPL - ECC - ECP	MPL - ECC - ECP	MPL - ECC - ECP	MPL - ECC - ECP
T_A	0.503 - **0.945** - 0.931	0.598 - **0.840** - 0.838	0.598 - **0.840** - 0.838	0.380 - **0.865** - 0.861
T_B	0.738 - **0.896** - 0.867	0.588 - **0.913** - 0.911	0.588 - **0.913** - 0.911	0.531 - **0.871** - 0.869
T_C	0.855 - **0.915** - 0.906	0.673 - 0.552 - **0.568**	0.673 - 0.552 - **0.568**	0.614 - 0.627 - **0.640**
T_D	0.554 - **0.934** - 0.929	0.494 - **0.767** - 0.755	0.494 - **0.767** - 0.755	0.482 - 0.786 - **0.787**
T_E	0.826 - **0.876** - 0.871	0.548 - 0.751 - **0.754**	0.548 - 0.751 - **0.754**	0.499 - **0.754** - 0.724
T_F	0.646 - **0.964** - 0.963	0.666 - 0.853 - **0.855**	0.666 - 0.853 - **0.855**	0.412 - **0.881** - 0.878
T_G	0.882 - **0.937** - 0.928	0.485 - **0.723** - 0.699	0.485 - **0.723** - 0.699	0.583 - **0.771** - 0.767
T_H	0.621 - 0.968 - **0.967**	0.699 - 0.886 - **0.898**	0.699 - 0.886 - **0.898**	0.550 - 0.889 - **0.901**
T_I	0.919 - 0.928 - **0.940**	0.493 - **0.531** - 0.527	0.493 - **0.531** - 0.527	**0.626** - 0.621 - 0.624
T_J	0.588 - **0.908** - 0.903	0.860 - 0.898 - **0.902**	0.860 - 0.898 - **0.902**	0.541 - **0.720** - 0.719
T_K	0.468 - **0.874** - 0.838	0.567 - **0.923** - 0.903	0.567 - **0.923** - 0.903	0.352 - **0.751** - 0.736
T_N	0.353 - 0.453 - **0.477**	0.510 - **0.617** - 0.600	0.510 - **0.617** - 0.600	0.334 - **0.392** - 0.384
T_O	0.724 - 0.972 - **0.984**	0.524 - **0.668** - 0.662	0.524 - **0.668** - 0.662	0.440 - 0.730 - **0.738**

### Prostate cancer histology slides

Lawson et al. [33] demonstrated that persistent homology can successfully be used to evaluate features in prostate cancer hmatoxylin and eosin (H&E)–stained slides. Their dataset, available in the Open Science Framework [34], contains 5,182 RGB images of a resolution 512 × 512 corresponding to different regions of interest (ROIs) in prostate cancer H&E slices obtained from 39 patients. Each image is labeled with a Gleason score of 3, 4, or 5 indicating the architectural patterns of the cancer. A higher Gleason score indicates an increasing level of cancer aggressiveness. The datasets contains 2,567 grade 3 ROIs, 2,351 grade 4 ROIs, but only 264 grade 5 ROIs. Given the imbalance in the data, we decided to consider a classification problem between grades 3 and 4.

Following the procedure described by the authors, we normalized and extracted the H&E color channel from each ROI. By doing so, we converted each RGB image into a bidimensional (H&E) one (Fig. 16). We first computed the ECC for each of the grayscale images corresponding to the hematoxylin channel as it is the color that highlights cell nuclei. We then also used the eosin color channel to obtain a 2-dimensional ECP (Fig. 17). We input either the ECCs or the ECPs into an support vector machine [35] classifier and computed the mean test accuracy over 100 rounds with a 80/20 training split. The results are displayed in Table 4. The classifier using as input the 2-dimensional ECPs is consistently performing better than the one using the 1-dimensional ECCs.

**Table 4:** Mean test accuracy for the Gleason 3 vs. Gleason 4 classification using ECCs or ECPs as input to a support vector machine classifier

Hematoxylin ECC	H&E ECP
0.765 ± 0.001	0.826 ± 0.001

## Conclusions

Euler characteristic curves and profiles provide a stable summary of the shape of data. Unlike other summaries used in topological data analysis, this one can be computed in a distributed fashion and hence is applicable to deal with big data problems. In addition, we show, contrary to a common misconception, that the Euler characteristic curves and profiles enjoy certain type of stability. We confirm it when using them to discriminate various toy datasets with varying levels of noise. We also show how to compare and vectorize the Euler characteristic curves and profiles and apply them to a number of real data analysis problems. The presented results are accompanied with efficient Python implementation. For example, on modern commodity hardware, our implementation for V-R complexes can handle a number of simplices on the order of 10^10^. This is a 2-order magnitude more than what can be achieved using available software like GUDHI [9]. With this work, we hope that the machinery of Euler characteristic curves and profiles will be useful for practitioners in topological data analysis.

## Supplementary Material

giad094_GIGA-D-23-00240_Original_Submission

giad094_GIGA-D-23-00240_Revision_1

giad094_GIGA-D-23-00240_Revision_2

giad094_Response_to_Reviewer_Comments_Original_Submission

giad094_Response_to_Reviewer_Comments_Revision_1

giad094_Reviewer_1_Report_Original_Submission Fan Wang, Ph.D -- 9/9/2023

giad094_Reviewer_2_Report_Original_Submission Liam Naughton -- 9/12/2023

## Figures and Tables

**Figure A1:**
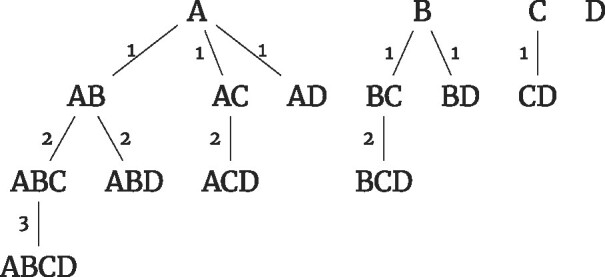
Simplex tree for a 4-clique with the update filtration cost.

**Figure A2:**
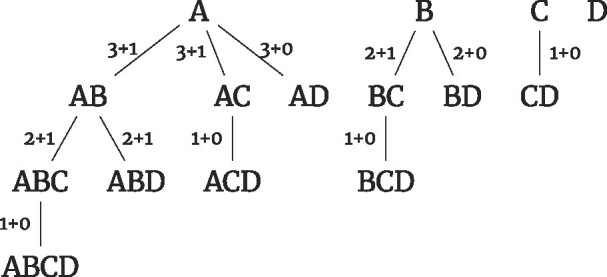
Simplex tree for a 4-clique with the update common neighbors cost.

## Data Availability

Snapshots of our code and other data further supporting this work are openly available in the *GigaScience* repository, GigaDB [36].

## Availability of Source Code and Requirements

- Project name: pyEulerCurves
- Project homepage: https://github.com/dioscuri<plxhyp>PLXHYP</plxhyp>tda/pyEulerCurves
- Operating system: platform independent
- Programming language: Python, C++
- License: MIT
- Any restrictions to use by nonacademics: none
- RRID: SCR_024472

Jupyter notebooks to reproduce all the experiments described in this article are available in the GigaDB repository [36], and the workflows are described in [37].

## Abbreviations

ECC: Euler characteristic curve; ECP: Euler characteristic profile; H&E: hematoxylin and eosin; LDA: linear discriminant analysis; MPH: multiparameter persistent homology; rLDA: regularized linear discriminant analysis; rQDA: regularized quadratic discriminant analysis; TDA: topological data analysis.

## Appendix A.

### Time performance analysis

We asses the time performance of Algorithm 1 by analyzing the worst-case scenario, a complete graph built from a pointcloud {*x*_*i*_} *i* ∈ [1, *n*]. This is the worst-case scenario as it contains the maximal number of cliques (hence simplices) for a given number of vertices, namely, 2^*n*^ − 1. As discussed in the previous section, the running time will be dominated by the first vertex *x*_1_ as it has the highest number of successive neighbors. The most time-consuming operations are the ones that happen inside Algorithm 2, namely, the update filtration and update common neighbors subroutines.

#### Update filtration

The extension of a *d*-clique requires checking whether 1 or more of the new introduced edges have a filtration value higher than the current *d*-clique. Comparison between floats can be done in constant time and has to be repeated *d* times. With reference to Fig. A1, we can assign to each edge in the simplex tree a cost that depends only on the edge depth. The total sum of such cost is

\begin{eqnarray*}
\sum _{i=1}^n (i-1) \binom{n}{i} = 2^{n - 1} n - 2^n + 1 \quad . \end{eqnarray*}

In case of perfect parallelization, the cost for the first vertex only is

\begin{eqnarray*}
\sum _{i=1}^{n - 1} i \binom{n - 1}{i} = 2^{n - 2} (n - 1) \quad . \end{eqnarray*}

#### Update common neighbors

Updating the list of common neighbors after a clique extension requires computing the intersection between the current list of common neighbors (with length *m*_1_) and the list of neighbors of the newly added vertex (with length *m*_2_). Given that such lists are ordered, their intersection can be computed in $\mathcal {O}(m_1 + m_2)$. The total cost for this operation can be obtain recursively by observing that the number of neighbors in a clique is uniquely determined—in this particular case—only by the last element of the clique. For example, in Fig. A2, the subtree spanning from ab is the same as the one spanning from b, and the one from ac is equivalent to c. The total cost for a clique of size *n* can be then expressed as twice the cost for the (*n* − 1)—clique plus the cost of the depth 1 edges spanning from the first vertex:

\begin{eqnarray*}
c(n) &=& \frac{(n-1)(n-2)}{2} + 2c(n-1) + (n-1)^2 \quad ;\\ c(2) &=& 1 \quad . \end{eqnarray*}

Such recurrence has solution

\begin{eqnarray*}
c(n) = \frac{1}{2} (-8 + 2^{n+3} - 5 n - 3 n^2) \quad . \end{eqnarray*}

In case of perfect parallelization, the cost for the first vertex only is

\begin{eqnarray*}
c(n) - c(n-1) = 2^{n+1} - 3 n - 1 \quad . \end{eqnarray*}

### Memory performance analysis

At each step, the algorithm needs to store in memory the local graph *G* with each edge’s filtration value. The local graph can be stored as an adjacency matrix whose entries represent the filtration values. Moreover, we need to store the current list of simplices, the list of their filtration values, and the list of common neighbor for each simplex. Let us denote with *V* the bits needed to store an edge label (usually a uint) and with *F* the bits needed to store a filtration value (usually a float). Assuming the worst-case scenario of a fully connected graph with *n* nodes, the maximum number of simplices will be generated at dimension $\frac{n}{2}$ and will be $\binom{n}{n/2}$. The memory cost at that step will then be $\mathcal {O}(n(n-1)F + \binom{n}{n/2}nV + \binom{n}{n/2}F) = O \left( \frac{2^n}{\sqrt{n}} (nV + F) \right)$, where the first term is the graph cost, the second one is the cost of the list of simplices, and the list of common neighbors that we assume to have the same size due to the symmetry of the binomial coefficients, and the third one is the cost of the simplices filtration values.
