# Supplementary material for: Euler characteristic curves and profiles: a stable shape invariant for big data problems
Source: Gigascience. 2023 Nov 15;12:giad094. doi: 10.1093/gigascience/giad094 (PMC10646871; doi:10.1093/gigascience/giad094)
Supplement: giad094_GIGA-D-23-00240_Revision_1 [file giad094_giga-d-23-00240_revision_1.pdf]

GigaScience

Euler Characteristic Curves and Profiles: a stable shape invariant for big data problems

--Manuscript Draft--

**Manuscript Number:**	GIGA-D-23-00240R1
**Full Title:**	Euler Characteristic Curves and Profiles: a stable shape invariant for big data problems
**Article Type:**	Research
**Funding Information:**	Narodowe Centrum Nauki — Dr Paweł Dłotko<br>Max-Planck-Gesellschaft — Dr Paweł Dłotko
**Abstract:**	Tools of Topological Data Analysis provide stable summaries encapsulating the shape of the considered data. Persistent homology, the most standard and well studied data summary, suffers a number of limitations; its computations are hard to distribute, it is hard to generalize to multifiltrations and is computationally prohibitive for big datasets. In this paper we study the concept of Euler Characteristics Curves, for one parameter filtrations and Euler Characteristic Profiles, for multi-parameter filtrations. While being a weaker invariant in one dimension, we show that Euler Characteristic based approaches do not possess some handicaps of persistent homology; we show efficient algorithms to compute them in a distributed way, their generalization to multifiltrations and practical applicability for big data problems. In addition we show that the Euler Curves and Profiles enjoys certain type of stability which makes them robust tools for data analysis. Lastly, to show their practical applicability, multiple use-cases are considered.
**Corresponding Author:**	Davide Gurnari<br>IM PAN: Instytut Matematyczny Polskiej Akademii Nauk<br>Warsaw, POLAND
**Corresponding Author Secondary Information:**	
**Corresponding Author's Institution:**	IM PAN: Instytut Matematyczny Polskiej Akademii Nauk
**Corresponding Author's Secondary Institution:**	
**First Author:**	Paweł Dłotko
**First Author Secondary Information:**	
**Order of Authors:**	Paweł Dłotko<br>Davide Gurnari
**Order of Authors Secondary Information:**	
**Response to Reviewers:**	Dear Editor,<br><br>We thank you and the Reviewers for evaluating our manuscript and we are very pleased with the outcome. We took the Reviewers commitment into high consideration and we revised the manuscript accordingly. Please find the revised version attached, the additions in page 1 and 8 are identified by the teal colour.<br><br>A point-by-point response to the Reviewers' comments can be found below.<br><br>Lastly, we registered our software implementation in bio.tools and our experiments' workflow in workflowhub.eu and added the relevant identifiers to the Code availability section.<br><br>Thank you for considering our work, we will be very happy to answer any further questions.<br><br>Kind regards,

Powered by Editorial Manager® and ProduXion Manager® from Aries Systems Corporation

Davide Gurnari
Paweł Dłotko

============

Reviewer #1: The authors present an insightful analysis of the stability of Euler characteristic curves with respect to the 1-Wasserstein distance. They further introduce a novel extension to a multi-filtration setting termed as the Euler characteristic profile. The authors then offer two distributed algorithms tailored for computing ECC for Vietoris-Rips and cubical complexes. The culmination of their research effort is the vectorization of the Euler characteristic curves and profiles to be utilized as inputs for a classifier. The efficacy of this approach is corroborated using a classification task on a prostate cancer histology slides dataset.

While the paper is cogent and well-organized, there are minor grammatical errors that slightly mar its overall clarity. The introduction of a multi-filtration setting offers fresh insights and can be particularly advantageous for certain tasks. The paper's technical foundation is robust, and the expounded concepts are self-explanatory. The main objective is to compute Euler Characteristic-based features in a distributed manner to cater to big data challenges. Yet, a possible point of contention is the lack of a thorough justification for the need for distributed algorithms. Given that a distributed system setting isn't ubiquitously available in a standard computer science laboratory, the rationale behind this choice could be elaborated upon.

PD-DG: We agree with Reviewer #1 that a distributed system is not available to every practitioner. However, the algorithms we propose are also perfectly parallelizable and our software implementation can take advantage of multi-core CPU that are nowadays the standard even in consumer-grade hardware. For example, the experiment shown in Figure 6 was conducted on an AMD Ryzen Threadripper CPU which can be found on mid-tier desktop workstations. Moreover, time performance benefits can be observed even when running our software on laptop CPUs with “only” 4 or 8 cores.
The need for truly distributed implementations arises when the size of the input data is too large to fit into the system’s memory. Our algorithms can be easily executed on a computer cluster. We added a comment in the introduction (page 1) to better clarify this aspect.
To summarize, although we are not presenting examples where distributed implementation is required, we hope that the proposed mechanism of stable Euler Characteristics Curves and Profiles will be useful to solve multiple projects among which some large scale projects may arise.

Reviewer #1: Furthermore, the authors could benefit from explicitly elucidating why streaming algorithms, such as those mentioned in references [15] and [16], aren't directly adaptable to the multi-filtration context.

PD-DG: We thank Reviewer #1 for this comment. The algorithms in [15] and [16] store the cumulative change in the EC at the voxel’s (the top dimensional cells) filtration value. This requires sorting the voxel’s value from the lowest to highest. While such a sorting can be done for one value filtration, it cannot be performed for a multifiltration, as no good ordering exists in the general case. An appropriate comment has been added to the text in page 8.

Reviewer #1: While there are certain drawbacks to consider, the TDA community might find value in this research, especially with its exploration of the multi-filtration aspect.

============

Reviewer #2: This paper proposes an Euler characteristic curves based approach as an alternative to persistent homology as a tool in topological data analysis. The approach utilised provides a stable summary of the shape of data which can be computed in a distributed fashion - a key requirement for 'big data' type problems. The

Powered by Editorial Manager® and ProduXion Manager® from Aries Systems Corporation

	approach is novel and builds upon earlier work on Euler characteristic surfaces. The algebraic contribution includes proofs of a number of results which focus exclusively on Betti curves thereby avoiding a complication arising from any attempt to apply the methods used by Chung and Lawson in [24].<br><br>Algorithm 1 returns an ordered list of pairs capturing the contribution of each cell at the cell filtration level as well as the sign of the contribution to the Euler characteristic computed as the alternating sum of the number its cells in each dimension.<br><br>The authors include an application to cubical complexes utilising the T-construction outlined in Algorithm 3 which offers an alternative to existing approaches and can also be generalized to the multi-parameter filtration case. Algorithms 1 and 3 are easily extended to compute the Euler Characteristic Profile of multi-filtered V-R and cubical complexes. A difficulty is highlighted in the calculation of distances between Euler Characteristic Profiles with a nod towards potential future work searching for more efficient algorithms.<br><br>The paper includes a toy example using 3 dimensional ECPs applied to RGB images as well as applications to real world examples involving immune cell spatial patterns in tumours and analysis of prostate cancer slides. They use classification techniques including variations of discriminant analysis as well as support vector machines to compare their algorithms to those utilised in previous studies. Results suggest that the approaches described can offer superior accuracy compared to the literature and this is clear evidence that the work has significant merit and has the potential to be utilised by practitioners of topological data analysis.<br><br>The paper is well written and well structured and also includes a useful time & memory performance analysis as an appendix. I recommend that the paper be accepted and I compliment the authors on the novel application of concepts drawn from the heart of algebraic topology to problems in topological data analysis and image recognition.<br><br>In addition, Reviewer #2 highlighted the minor corrections as follows:<br><br>- Page 1 last bullet point 'real word data'<br>- Page 3 Section Stability of Euler Characteristic Curves and Pro-<br>files '....some know(n) topological quantity'<br>- Page 6 Section Algorithms '...depending (on) the dimension'<br>- Page 6 just above Algorithm 1 - I suspect there is a mathematical typo here (-1)^dim(\sigma), f(\sigma)) I think the order of the components in the pair is the wrong way round - suggest asking authors to clarify<br><br>PD-DG: We thank Reviewer #2 for the kind comments, we corrected the typos mentioned above.
**Additional Information:**	
**Question**	**Response**
Are you submitting this manuscript to a special series or article collection?	No
**Experimental design and statistics**<br><br>Full details of the experimental design and statistical methods used should be given in the Methods section, as detailed in our Minimum Standards Reporting Checklist. Information essential to interpreting the data presented should be made available in the figure legends.	Yes

Powered by Editorial Manager® and ProduXion Manager® from Aries Systems Corporation

Have you included all the information requested in your manuscript?	
**Resources**<br><br>A description of all resources used, including antibodies, cell lines, animals and software tools, with enough information to allow them to be uniquely identified, should be included in the Methods section. Authors are strongly encouraged to cite Research Resource Identifiers (RRIDs) for antibodies, model organisms and tools, where possible.<br><br>Have you included the information requested as detailed in our Minimum Standards Reporting Checklist?	Yes
**Availability of data and materials**<br><br>All datasets and code on which the conclusions of the paper rely must be either included in your submission or deposited in publicly available repositories (where available and ethically appropriate), referencing such data using a unique identifier in the references and in the “Availability of Data and Materials” section of your manuscript.<br><br>Have you have met the above requirement as detailed in our Minimum Standards Reporting Checklist?	Yes

Powered by Editorial Manager® and ProduXion Manager® from Aries Systems Corporation

Revised manuscript

Click here to access/download;Manuscript;ECP_GigaScience_REVISED.pd

GigaScience, 2023, 1–15

doi: xx.xxxx/xxxx
Manuscript in Preparation
Paper

PAPER

Euler Characteristic Curves and Profiles: a stable shape invariant for big data problems

Paweł Dłotko[1] and Davide Gurnari[1,†]

[1]Dioscuri Centre in Topological Data Analysis, Mathematical Institute, Polish Academy of Sciences, Warsaw, Poland

[†]dgurnari@impan.pl

Abstract

Tools of Topological Data Analysis provide stable summaries encapsulating the shape of the considered data. Persistent homology, the most standard and well studied data summary, suffers a number of limitations; its computations are hard to distribute, it is hard to generalize to multifiltrations and is computationally prohibitive for big datasets. In this paper we study the concept of Euler Characteristics Curves, for one parameter filtrations and Euler Characteristic Profiles, for multi-parameter filtrations. While being a weaker invariant in one dimension, we show that Euler Characteristic based approaches do not possess some handicaps of persistent homology; we show efficient algorithms to compute them in a distributed way, their generalization to multifiltrations and practical applicability for big data problems. In addition we show that the Euler Curves and Profiles enjoys certain type of stability which makes them robust tools for data analysis. Lastly, to show their practical applicability, multiple use-cases are considered.

Key words: Topological Data Analysis, Persistence Homology, Euler Characteristic, Distributed Computations

Introduction

Topological Data Analysis since its beginning [1], [2] has brought attention in the data science community. Topological tools, like persistent homology [3] and mapper [2] were used in multiple tasks in material science [4], [5], [6], medicine [7] and many more. In time, persistent homology has been successfully integrated with machine learning pipelines, and mapper became an exploratory data analysis tool. In this work we will extend on the path of persistent homology. With its successes, attempts were made to apply it in task of big data analysis. However, the progress is minimal. While there exist a single distributed implementation [8], it does not scale up and was not extensively used in big data analysis. In practice, mostly various sequential implementations are used [9]. To bypass the problem of too large input, a number of sparsification techniques [10], [11] as well as bootstrap [12] and zig-zag [13] approaches were proposed. While they scale up to problems of a certain size, they tend to bypass the big data challenge rather than proposing a solution for it.

In this paper we extend the tool of classical Euler characteristic and Euler characteristic curves. The new contributions include:

- A proof of stability of the Euler Characteristic Curve (ECC) with respect to the 1-Wasserstein distance between persistence diagrams;
- A generalization of the Euler Characteristic Curve to the multiparamenter filtration case, with arbitrary number of parameters, that we denote as Euler Characteristic Profile (ECP);
- An analysis of the stability of such ECPs;
- Distributed algorithms to compute the exact ECC for Vietoris-Rips and cubical complexes that can be naturally extended to the multiparameter case. An Python implementation of such algorithms is provided as scikit-learn [14] compatible package.
- Discussion of methods to compare and vectorize ECCs and ECPs;
- Examples of applications of the ECC/ECP to real world data.

Our proposed algorithms are perfectly parallelizable and our software implementation can take advantage of multi-core CPU that are nowadays the standard even in consumer-grade hardware. Moreover, they can also be easily executed on a computer cluster to tackle scenarios when the size of the input data is too large to fit into the system's memory. While we are not aware of any distributed algorithm to compute Euler Characteristic Curves of a Vietoris-Rips complex, Heiss and Wagner [15] describe a streaming algorithm

Compiled on: September 25, 2023.
Draft manuscript prepared by the author.

to compute the ECC from cubical complexes which has also been adapted for GPU computations [16]. While their implementation is very fast we see no straightforward way to generalize it to the multiparameter filtration case. To the best of our knowledge the concept of Euler Characteristic Profiles of arbitrary dimension is novel in the literature. There are however some works that focus on the bifiltration case, known as Euler Characteristic Surfaces. It was used in an applied setting by Roy et al. [17] to analyze drying droplets but no topological background is provided. Beltramo et al. [18] gave a description of Euler Characteristic Surfaces in the persistence homology framework and apply it to obtain a descriptor of both pointcloud and image based data. Moreover, they provide a Python implementation of their algorithms which however requires the input bifiltration to be binned. Chen et al. [19] introduced a time-aware multipersistence Euler-Poincaré surface to describe dynamical networks and proved its weak L_1 stability. A recent preprint by Perez [20] analyzes the stability of Euler and Betti curves of stochastic processes on compact Riemannian manifolds.

Euler characteristic curves (and profiles)

In this section we introduce the essential mathematical concepts needed to define Euler Characteristic Curves and Profiles. For an exhaustive presentation we refer to classic textbooks like [21] and [3].

Definition 1. A *CW* or *cell complex* X is a topological space that can be built up starting from a discrete set X^0 of 0-dimensional cells and then inductively creating the n-skeleton X^n by attaching n-cells to X^{n-1} along their boundary. The process can be stopped at some finite dimension or can continue indefinitely. A subset $A \subseteq X$ is a *subcomplex* of X if with each cell of A, all its lower dimensional cells enters A.

Remark 1. Since we are interested in applying this machinery to analyze real word data we will always assume that our complexes are finite.

While the theory can be built in the general CW complex setting, the algorithms we present in Section Algorithms are specific to two different specializations that are used to represent different types of data: simplicial and cubical complexes.

Definition 2. An *abstract simplicial complex* is a finite collection of sets K such that $\sigma \in K$ and $\tau \subseteq \sigma$ implies $\tau \in K$. The sets in K are called *simplicies* and the *dimension* of a simplex is $dim(\sigma) = card(\sigma) - 1$. We will often refer to 0-simplices as *vertices*, and to 1-simplices as *edges*. Given a simplex $s = \{v_0, \dots, v_k\}$, its *boundary* is $\partial s = \sum_{i=0}^{k} (-1)^i \{v_0, \dots, \hat{v_i}, \dots, v_k\}$, where $\hat{v_i}$ denotes that the vertex v_i is removed from the simplex. Simplices $\{v_0, \dots, \hat{v_i}, \dots, v_k\}_{i=0}^{k}$ are in the boundary of s.

There are different ways of obtaining an abstract simplicial complex from point cloud data such as the Čech, the Vietoris-Rips and the Alpha constructions [3], in Section Vietoris-Rips complexes we describe the Vietoris-Rips construction.

Definition 3. An *elementary interval* is a subset of $\mathbb{R}$ of the type $I = [l, l+1]$ or $I = [l, l]$, for some integer l. The first type is called non-degenerate interval while the second is a degenerate interval. An *elementary cube* C is a product of elementary intervals $C = I_1 \times \dots \times I_n$ and its dimension is the number of non-degenerate intervals in the product. The *boundary* of an elementary interval is $\partial[l, l+1] = [l+1, l+1] + [l, l]$ and $\partial[l, l] = 0$. The boundary of an elementary cube is then defined as $\partial C = \partial(I_1 \times \dots \times I_n) = \sum_{i=1}^{n} I_1 \times \dots \times \partial I_i \times \dots \times I_n$. Similarly to the simplicial complex case, a *cubical complex* K is a collection of elementary cubes closed under operation of taking boundary

One of most common use case of cubical complexes is image data. In Section 16 we describe how to build a filtered cubical complex from an n-dimensional image, by identifying the image's pixels with top dimensional cells.

In what follows we will refer to simplices and cubes, as elements of a simplicial or a cubical complex jointly as *cells* in a *cell complex*. A cell τ is said to be a *face* of σ if τ is in the boundary of σ.

Definition 4. Let K be a cell complex and d a dimension. A *d-chain* is a formal sum of d-cells in K, namely $c = \sum a_i \sigma_i$ where the σ_i are the d-cells and the a_i are the coefficients.

There are many possible choices for the group of coefficients. A standard approach in computational topology is to use *modulo 2 coefficients*, i.e. the a_i can be either 0 or 1 and satisfy $1 + 1 = 0$ [1]. Other options include integer, rational or real coefficients.

Two d-chains can be added component-wise. Namely, given $c = \sum a_i \sigma_i$ and $c' = \sum b_i \sigma_i$, $c + c' = \sum (a_i + b_i)\sigma_i$. Therefore, we can define the *group of d-chains* $\mathbf{C}_d = \mathbf{C}_d(K)$. The boundary of a d-chain is the sum of the boundaries of its cells $\partial c = \sum a_i \partial \sigma_i$, which is a $(d-1)$-chain. Since the boundary commutes with the addition operation, we can define -for each dimension d- the *boundary homomorphism* $\partial_d : \mathbf{C}_d \to \mathbf{C}_{d-1}$.

A *d-cycle* is a d-chain with empty boundary $\partial c = 0$. A *d-boundary* is a d-chain which is the boundary of a $(d+1)$-chain. Since ∂ commutes with addition, we have the *group of d-cycles* $\mathbf{Z}_d = \mathbf{Z}_d(K)$, and the *group of d-boundaries* $\mathbf{B}_d = \mathbf{B}_d(K)$. It is a fundamental result that $\partial_d \partial_{d+1} c = 0$ for every dimension d and every $(d+1)$-chain c. This means that the boundary of a boundary is always zero, in other words $\mathbf{B}_d$ is a subgroup of $\mathbf{Z}_d$. This leads to the following definition.

Definition 5. The *d-th homolgy group* is the d-th cycle group modulo the d-th boundary group, $\mathbf{H}_d = \mathbf{Z}_d / \mathbf{B}_d$. The *d-th Betti number* is the rank of this group, $\beta_d = \text{rank}(\mathbf{H}_d)$.

Definition 6. Let K be a cell complex. A *filtration* of K is a sequence of nested subcomplexes $\emptyset = K_0 \subseteq K_1 \subseteq \dots \subseteq K_n = K$. Such sequence is finite for finite complexes. It can be obtained by means of a *filtration function* over K, a monotonic non-decreasing function $f : K \to \mathbb{R}$ such that $f(\tau) \leq f(\sigma)$ if τ is a face of σ. Note that every *sublevel set* $K_t = f^{-1}(-\infty, t]$ is a subcomplex of K for every $t \in \mathbb{R}$.

For each dimension d, such a filtration corresponds to a sequence of homology groups $0 = \mathbf{H}_d(K_0) \to \mathbf{H}_d(K_1) \to \dots \to \mathbf{H}_d(K_n) = \mathbf{H}_d(K)$. For every $i < j$, the homomorphism $f_d^{i,j} : \mathbf{H}_d(K_i) \to \mathbf{H}_d(K_j)$ is induced from the inclusion map of K_i into K_j.

Definition 7. The *d-th persistent homology groups* are the images of the homomorphisms $\mathbf{H}_d^{i,j} = \text{im} f_d^{i,j}$. The ranks of these groups are the *d-th persistent Betti numbers* $\beta_d^{i,j} = \text{rank}(\mathbf{H}_d^{i,j})$.

Intuitively, the d-th persistent Betti number $\beta_d^{i,j}$ counts how may homology classes of K_i are still present in K_j. There are two scenarios in which a homology class from K_i may not be present in K_j - it may either became trivial, or it may became identical (homologous) to a class that was created earlier.

Definition 8. The k-th dimensional *persistence diagram* of a filtered complex K, $Dgm_k(K)$ is a multiset of points in the extended real plane $(\mathbb{R} \cup \{\infty\}) \times (\mathbb{R} \cup \{\infty\})$. The multiplicity of each point (b, d) indicates the number of independent k-dimensional classes that are born at filtration value b and die at filtration value d.

All the points on the diagonal are always included, with countable multiplicity, in a persistence diagram, in order to make sense of the following.

1 Using modulo 2 coefficients allows us to get rid of the $(-1)^i$ in the definition of the boundary in 2

Definition 9. A *matching* of two persistence diagrams C and D is a bijection $\eta : C \to D$ possibly to or from points on the diagonal.

Definition 10. The 1-Wasserstein distance between two $k-$dimensional persistence diagrams C, D is

$$W_1(C, D) = \inf_{\eta : C \to D} \sum_{(b,d) \in C} \|(b, d) - \eta(b, d)\|_\infty^1$$

where η is a matching of C and D.

Definition 11. The *Euler Characteristic* of a cell complex K is the alternating sum of the number of its cells in each dimension

$$\chi(K) = \sum_d (-1)^d |K^d| \quad .$$

Where K^d denotes the d dimensional cells in K. Thanks to the Euler-Poincaré formula, the Euler characteristic can also be expressed as the alternating sum of the Betti numbers, the ranks of the cell complex's homology groups: $\chi(K) = \sum_d (-1)^d \beta_d(K)$ [3].

Definition 12. Let us consider a filtered complex K with filtration function $f : K \to \mathbb{R}$. We can define its *Euler Characteristic Curve* as a function that assign an Euler number χ for each filtration level $t \in \mathbb{R}$

$$ECC(K, t) = \chi(K_t) \quad .$$

Recall that $K_t = f^{-1}(-\infty, t]$ is a subcomplex of K for every $t \in \mathbb{R}$.

We are now interested in extending the concept of Euler Characteristic Curve to the more general Multidimensional Persistence setting [22]. In order to do so, we need to generalize Definition 6 to families of nested complexes indexed by posets. While Multidimensional Persistence is a vibrant and active research topic, in this paper we will only make use of the basic concepts. We refer the interested reader to [23] for a modern introduction to the topic.

Definition 13. Let K be a cell complex and P a poset. A *P-indexed filtration* on K is a family of nested complexes such that K_x is a subcomplex of K for each $x \in P$, and $K_x \subseteq K_y$ whenever $x \leq y$. If $P = T_1 \times \cdots \times T_n$ where each T_i is a totally ordered set, we call a *multiparameter* or *n-parameter filtration*.

It is a natural question to ask whether the idea of sublevel sets of a filtration function could be extended too. In general, this is not the case. It can be achieved only when each cell of K first appears in the filtration at some unique minimal index in P.

Definition 14. Let K be a cell complex, P a poset and f a function $f : K \to P$. The *sublevelfiltration* of f is a family of complexes of the type

$$K_p = \{\sigma \in K \mid f(\sigma) \leq p\} .$$

A filtration isomorphic to a sublevel filtration is said to be *1-critical*. A filtration that is not 1-critical is said to be *multicritical*.

Definition 15. The *Euler Characteristic Profile* (ECP) of a P-filtered complex K is a function that assign to any value $p \in P$ the Euler characteristic of the corresponding subcomplex K_p.

$$ECP(K, p) = \chi(K_p) \quad .$$

For the rest of the paper we will focus on the case $P = \mathbb{R}^n$.

Remark 2. The two dimensional ECP already appeared in the literature and it is known as Euler Characteristic Surface [17], [18], [19]. It was however defined only for the Cartesian product of two one-parameter filtrations and it is treated as matrix in the following way. Given a bi-filtering function $F : K \to \mathbb{R}^2$ over K and a set of threshold values $I = \{(a_i, b_j) \mid 1 \leq i \leq m, 1 \leq j \leq n\}$, the Euler Characteristic Surface is the $m \times n$ integer valued matrix S whose entries are $S_{ij} = \chi(K_{ij}) = \chi(F^{-1}\left((-\infty, a_i] \times (-\infty, b_j]\right)$. This matrix representation corresponds to sampling the two dimensional profile on the grid given by I. In general the choice of such grid is not unique, and the spacing of such grid may not be constant. This makes it difficult to define a general notion of distance between Euler Characteristic Surfaces matrices. For this reason we think it is more natural to define the Euler Characteristic Profile as a function like in 15 and look for stability results in this setting.

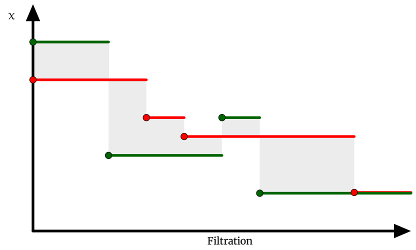

Figure 1. Two Euler Characteristic Curves in red and green. The absolute value of their difference is highlighted in shaded gray.

Stability of Euler Characteristic Curves and Profiles

The goal of this section is to find a bound for the distance between Euler Characteristic Curves by some known topological quantity of the point cloud that is robust with respect to small perturbations of the point cloud. This way, the stability of Euler Characteristic Curves is obtained.

Euler Characteristic Curves

Since ECCs are are piecewise constant functions, we consider the L_1 distances between them.

Definition 16. Let K_1 and K_2 be two filtered cell complexes. The L_1 distance between their Euler Characteristic Curves is

$$||ECC(K_1, t) - ECC(K_2, t)||_1 = \int_{\mathbb{R}} |ECC(K_1, t) - ECC(K_2, t)| dt \quad .$$

The proof presented in this section is inspired by the stability result for persistence functions by Chung and Lawson [24]. They analyze stability of a wide class of persistence curves and obtain a general bound (see Theorem 1 in [24]). However, trying to specialize this result to the simple Betti curve case leads a term that depends on the number of points in the Persistence Diagram. Hence the authors claim that Betti curves are unstable.

We will instead carry out the proof focusing exclusively on Betti curves, by doing so a stability result can be obtained.

Definition 17. Let K be a cell complex with filtration function f. Its $k-$th Betti curve is a function that assigns to each filtration level the $k-$th Betti number of the corresponding subcomplex.

$$\beta_k(K, t) = \beta_k(K_t) \quad .$$

Let now D be the $k-$dimensional persistence diagram obtained from a filtered complex K. The Fundamental Lemma of Persistent Homology [3] states that the $k-$th Betti number of the subcomplex K_t can be obtained by counting the points in the diagram that lie in the box $(x, y) \mid x \leq t < y$,

$$\beta_k(K_t) = \#[(b, d) \in D \mid b \leq t < d].$$

We can reformulate this statement by assigning to each point (b, d) in the diagram its indicator function in the interval $[b, d)$, $I_{[b,d)}(t) = 1$ if $t \in [b, d)$ and 0 otherwise. This indicator functions are exactly the bars in the barcode representation. By doing so we can define the k-dimensional Betti curve as the step function obtained by summing up all these indicator functions.

Definition 18. The $k-$th Betti curve for a persistence diagram D with finitely many off diagonal point is

$$\beta_k(D, t) = \sum_{(b,d)\in D} I_{[b,d)}(t).$$

Proposition 1. *Let C and D be two k-dimensional persistence diagrams. Their Betti curves are stable with respect to the 1-Wasserstein distance,*

$$\|\beta_k(C, t) - \beta_k(D, t)\|_1 \leq 2W_1(C, D). \tag{1}$$

Proof. Let us consider two $k-$dimensional persistence diagrams C, D and assume the optimal matching under the 1-Wasserstein distance is known. Moreover let us index the points in each diagram as (b_i^C, d_i^C) and (b_i^D, d_i^D) so that points with matching indices are paired under the optimal matching. The case when points from one diagram are matched to diagonal is described in the case 2 below. We can then write the difference between the two Betti curves as following

$$\begin{aligned}\|\beta_k(C, t) - \beta_k(D, t)\|_1 &= \|\sum_i I_{[b_i^C,d_i^C)}(t) - I_{[b_i^D,d_i^D)}(t)\|_1 \\ &\leq \sum_i \|I_{[b_i^C,d_i^C)}(t) - I_{[b_i^D,d_i^D)}(t)\|_1.\end{aligned}$$

Let us focus on a single term of the sum, $\|h_i(t)\| = \|I_{[b_i^C,d_i^C)}(t) - I_{[b_i^D,d_i^D)}(t)\|_1$. Then, one of the following cases have to hold:

Case 1: $b_i^C \leq b_i^D \leq d_i^C \leq d_i^D$.

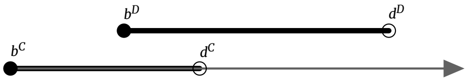

Figure 2. Case 1

$$\begin{aligned}\|h_i(t)\| &= \int_{b_i^C}^{b_i^D} |I_{[b_i^C,d_i^C)}(t)|dt + \int_{b_i^D}^{d_i^C} |I_{[b_i^C,d_i^C)}(t) - I_{[b_i^D,d_i^D)}(t)|dt \\ &\quad + \int_{d_i^C}^{d_i^D} |I_{[b_i^D,d_i^D)}(t)|dt \\ &= \int_{b_i^C}^{b_i^D} |1|dt + \int_{b_i^D}^{d_i^C} |1 - 1|dt + \int_{d_i^C}^{d_i^D} |1|dt \\ &= |b_i^D - b_i^C| + |d_i^D - d_i^C| \\ &\leq 2\max(|b_i^D - b_i^C|, |d_i^D - d_i^C|).\end{aligned}$$

Case 2: $b_i^C \leq b_i^D \leq d_i^D \leq d_i^C$.

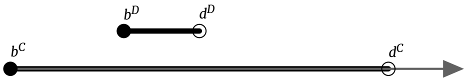

Figure 3. Case 2

$$\begin{aligned}\|h_i(t)\| &= \int_{b_i^C}^{b_i^D} |1|dt + \int_{b_i^D}^{d_i^D} |1 - 1|dt + \int_{d_i^D}^{d_i^C} |1|dt \\ &= |b_i^D - b_i^C| + |d_i^C - d_i^D| \\ &\leq 2\max(|b_i^D - b_i^C|, |d_i^D - d_i^C|).\end{aligned}$$

The matching of one point $(b_i^C, d_i^C) \in C$ with a point in the diagonal of D is a degenerate Case 2 with $b_i^C \leq b_i^D = d_i^D \leq d_i^C$. Note that, because of this, C and D are not required to have the same number of off-diagonal points.

Case 3: $b_i^C \leq d_i^C \leq b_i^D \leq d_i^D$

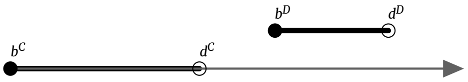

Figure 4. Case 3

This case will never happen as a better matching can always be obtained by matching both points to the diagonal, which is a degenerate Case 2.

We have that $\|h_i(t)\| \leq 2\max(|b_i^D - b_i^C|, |d_i^D - d_i^C|)$ holds for every i. We can then write the difference between two Betti curves as

$$\begin{aligned}\|\beta_k(C, t) - \beta_k(D, t)\|_1 &\leq \sum_i \|I_{[b_i^C,d_i^C)}(t) - I_{[b_i^D,d_i^D)}(t)\|_1 \\ &\leq \sum_i 2\max(|b_i^D - b_i^C|, |d_i^D - d_i^C|) \\ &= 2W_1(C, D).\end{aligned}$$

□

Thanks to the Euler-Poincaré formula, the Euler Characteristic Curve of a filtered complex K can be obtained as the alternating sum of its Betti curves.

$$ECC(K, t) = \sum_k (-1)^k \beta_k(K, t).$$

A stability result for the ECCs can be immediately derived from 1 assuming that the complex K has nonzero persistence diagrams in a finite number of dimensions, each of them containing a finite amount of off-diagonal points.

Proposition 2. *Let X and Y be two filtered cell complexes. The L_1 difference between the Euler Characteristic Curves of X and Y is bounded by the sum of the 1-Wasserstein distances between the corresponding k-dimensional persistence diagrams $Dgm_k(X)$, $Dgm_k(Y)$.*

$$\|ECC(X, t) - ECC(Y, t)\|_1 \leq \sum_k 2W_1(Dgm_k(X), Dgm_k(Y)). \tag{2}$$

Where the sum is over all dimensions in which the persistence diagrams are non-empty.

Proof. It is an immediate consequence of 1 and the triangular inequality.

$$\begin{aligned}\|ECC(X,t) - ECC(Y,t)\|_1 &= \|\sum_{k=0}^{n}(-1)^k(\beta_k(Dgm_k(X),t) - \beta_k(Dgm_k(Y),t)\|_1 \\ &\leq \sum_{k=0}^{n}\|\beta_k(Dgm_k(X),t) - \beta_k(Dgm_k(Y),t)\|_1 \\ &\leq \sum_{k=0}^{n} 2W_1(Dgm_k(X), Dgm_k(Y)).\end{aligned}$$

□

The above Proposition 2 is in explicit contrast with the claim that the Euler Characteristic Curve is unstable. In addition to the already mentioned work by Chung and Lawson [25], a similar statement can be found in [18] and [25].

Remark 3. With reference to Figure 1, the left-hand side in 2 is finite when the two ECCs agree from some filtration value onward. This is exactly what happens, for example, when considering curves obtained from full complexes, i.e. filtered complexes having a single simplex as a last element of a filtration: at some value all possible faces will have entered the filtration and so the Euler characteristic will stabilize at 1. If this does not happen the difference between the two ECCs will be unbounded. At the same time, it is straightforward to show that if two filtered complexes have different Euler characteristic at $+\infty$ their homologies will have a different number of essential classes. This translates to a different number of points at infinity in the persistence diagrams, whose Wasserstein distance would then be unbounded. In this case, the above result will trivially be $+\infty \leq +\infty$.

Euler Characteristic Profiles

We can immediately extend the notion of L_1 distances between ECCs to work in the general case of n−dimensional ECPs.

Definition 19. Let K_1, K_2 be two multifiltered cell complexes. The L_1 distance between the corresponding n−dimensional Euler Characteristic Profiles is

$$||ECP(K_1, v) - ECP(K_2, v)||_1 = \int_{\mathbb{R}^n} |ECP(K_1, v) - ECP(K_2, v)| dv \quad .$$

It is natural to ask whether the stability result in 2 can be naturally extended to the multi-parameter case. In the existing literature, Chen et al. proposed the following weak L_1-metric in the case of bifiltered complexes (see Definition 3.2 in [19]). Let us remind the proposed construction; consider two cell complexes K_1 and K_2 with a bifiltration function $F : K_{1,2} \to \mathbb{R}^2$. Let us denote with f and g the two real valued functions in the bifiltrations such that $F(\sigma) = ((f(\sigma), g(\sigma)))$ for every cell σ. Moreover, let us index the threshold values of F as $I = \{(a_i, b_j) \mid 1 \leq i \leq m, 1 \leq j \leq n\}$. The idea behind Chen et al. construction is to fix one of of the two filtrations at a specific value and consider the distances between the single parameter persistence diagrams induced by the other filtration function. By considering the set of threshold values I as a matrix with i rows and j columns , they define the ith *column distance* for the k−dimensional PDs as $D_k^{i*}(K_1, K_2) = W_1(D_k^g(K_1^{i*}), D_k^g(K_2^{i*}))$. Similarly, the jth *row distance* is $D_k^{*j}(K_1, K_2) = W_1(D_k^f(K_1^{*j}), D_k^f(K_2^{*j}))$.

Definition 20 (Definition 3.2 in [19]). The weak L_1 metric between K_1 and K_2 is

$$D(K_1, K_2) = \max\{\sum_{k=0}^{M}\sum_{i=1}^{m} D_k^{i*}(K_1, K_2), \sum_{k=0}^{M}\sum_{j=1}^{n} D_k^{*j}(K_1, K_2)\}.$$

Being able to recover the single parameter case, they prove the following stability result.

Proposition 3 (Theorem 3.1 in [19]). *Let K_1, K_2 be two bifiltered cell complexes. The distance between the corresponding Euler Characteristic Surfaces is bounded by the weak L_1 metric metric between K_1 and K_2,*

$$||ECP(K_1, v) - ECP(K_2, v)||_1 \leq c \cdot D(K_1, K_2) \,,$$

for some $c > 0$.

This constructions appears to be the natural generalization to multifiltration case of the stability result in 2. However, there are some fundamental problems that undermine the usefulness of such weak L_1 metric.

Remark 4. In our opinion the sums over rows or columns in 20 should be replaced with integrals over the filtration ranges. As already discussed in Remark 2, this would allow for more flexibility when dealing with filtration thresholds whose spacing is not constant.

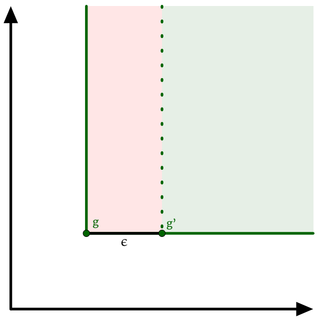

Figure 5. Minimal counterexample for the instability of ECP. Consider a cell complex made by only one vertex whose +1 contribution appears at some point $g = (g_1, g_2) \in \mathbb{R}^2$ and move it to $g' = (g_1 + \epsilon, g_2)$. Their difference, the region shaded in red, is unbounded

Remark 5. Proposition 3 will evaluate to a trivial $\infty \leq \infty$ in most cases, even the simplest one. Consider for example the situation depicted in Figure 5 of the ECP of a bifiltered complex K_1 made by just one 0-dimensional cell that appears at filtration value (g_1, g_2). The ECP will then be 1 in the cone $\{(x, y) \in \mathbb{R}^2 : x \geq g_1, y \geq g_2\}$ and 0 otherwise. We can obtain a different complex K_2 by perturbing the first filtration value by an ϵ amount $(g_1 + \epsilon, g_2)$. The difference between the two ECPs will then be unbounded. At the same time, also the weak L_1 distance between K_1 and K_2 will be unbounded because in the interval $[g_1, g_1 + \epsilon) \times [g_2, +\infty]$ the two complexes have a different number of essential classes and so the W_1 distance between the corresponding PDs will be infinite.

Because of the discussed issues, the stability result in [19], while being formally correct, does not cover a lot of practically relevant cases.

However, in most applications we can *truncate* the ECP, by limiting its filtration domain to i.e. the interval $[0, f_\infty]$ in every filtration dimension, where f_∞ is a finite value. Note that this value at infinity should not be the same as the maximum filtration value of the complex's cells, but it should be strictly larger than the maximum filtration value. For example, in the case of images whose pixels have integer filtration values in the $[0, 255]$ range (see Section 14) we could choose $f_\infty = 256$ as truncation value. By doing so, the distance between every pair of ECP will be finite but it will of course depend on the truncation value. Using truncation, we can state the following result.

Proposition 4. *Let K be a finite cell complex with a n-dimensional multifiltration $F : K \to \mathbb{R}^n$. We define K^ϵ as the complex obtained by perturbing the filtration values of each cell in K by at most ϵ in in l^∞ norm. Let us assume, for simplicity, that we truncate the domain of every filtration function to the same interval $[0, f_\infty]$. We then have the following bound*

$$||ECP(K, v) - ECP(K^\epsilon, v)||_1 \leq |K| \cdot d \cdot \epsilon^{n-1} \cdot f_\infty \quad ,$$

where $|K|$ is the number of cells in the complex and n is the number of filtration parameters.

Proof. Let us consider a single cell $\sigma \in K$ with filtration value $g = (g_1, \cdots, g_d)$. Its contribution to the ECP will be $(-1)^{dim(\sigma)}$ in the cone above g (i.e. for all points $x \in \mathbb{R}^d$ such that $g \leq x$ coordinate-wise). Let σ' be the corresponding cell in K^ϵ whose filtration values have been maximally perturbed to $g' = (g_1 + \epsilon, \cdots, g_d + \epsilon)$. The volume of the region which is in the cone of g but not on the cone of g' can be bounded by a sum of n n-dimensional cuboids of base ϵ^{n-1} and height f_∞, each of them corresponding to a shift of ϵ in the direction of one of the axis, $V_\sigma \leq d \cdot \epsilon^{n-1} \cdot f_\infty$, where the inequality is due to the fact that cuboids can have non-empty intersection. One of such cuboids is shaded in red in Figure 5. Multiplying by the total number of cells give us the bound. □

Algorithms

Recall that the Euler characteristic of a cell complex is the alternating sum of the number of its cells in each dimension. The contribution of each cell will thus be plus or minus one depending on the dimension of the cell. Moreover, this contribution will appear at the cell's filtration level. Therefore, if we are able to obtain a list of all cells with their filtration values we can compute the Euler characteristic at each filtration level. This is the main idea behind the following algorithms, which will always return what we will denote as LIST_OF_CONTRIBUTIONS, a list of pairs $(f(\sigma), (-1)^{dim(\sigma)})$ that stores each cell's contribution to the EC at the cell filtration level. Once this pairs have been sorted in ascending order with respect to the filtration, the Euler Characteristic Curve can be reconstructed by progressively summing up the contributions of following elements in the list.

Remark 6. Roune and Sáenz de Cabezón [26] proved that computing the Euler characteristic of a simplicial complex given by its vertices and facets is is #-P-complete. Even if their result does not mention filtered complexes, it follows from it that the problem of computing the ECC is at least P-complete. Otherwise, by contradiction, we could construct an arbitrary filtration of the considered complex, and look at the end value of the curve to obtain the Euler characteristic of the complex in polynomial time.

Vietoris-Rips complexes

In this section we will present a distributed algorithm to compute the Euler Characteristic Curve of a Vietoris-Rips simplicial complex obtained from a collection of points in $\mathbb{R}^n$.

Definition 21. Let X be a finite collections of points in $\mathbb{R}^n$, also denoted as a point cloud. Given a parameter $\epsilon \leq 0$, the *Vietoris-Rips complex* constructed from X is the collection of all subset of diameter at most 2ϵ, where the diameter is the greatest distance between any pair of vertices

$$\text{V-R}(X, \epsilon) = \{\sigma \subseteq X \mid diam(\sigma) \leq 2\epsilon\} \quad .$$

The filtration of each simplex is given by its diameter.

The Vietoris-Rips complex is a *flag complex*, this means that a subset S of vertices is in the complex if every pair of vertices in S is in the complex. This is analogous to saying that the Vietoris-Rips complex is completely determined by its 1-skeleton graph as there is a 1 to 1 correspondence between simplices in the complex and cliques in its 1-skeleton graph

Therefore, it is straightforward to see that listing all the simplices in a Vietoris-Rips complex is equivalent to perform a cliques count of its 1-skeleton graph [27]. In order to compute the contributions to the ECC we need to find an efficient and distributed way to list all cells in the simplex (i.e. all cliques in the 1-skeleton graph), and their filtration values (i.e. the length of the longest edge in each clique), this can be achieved in the following way. Given an ordered list of points $X = \{x_i : i \in [1, n]\}$[2] and a maximum distance ϵ, for each point x_i we build its local graph G_i of *subsequent neighbours*, namely all points $x_j \in B(x_i, \epsilon) \cap X$ with $j > i$. For each G_i we list all of its cliques that contain x_i. They will correspond to simplices with x_i being the smallest vertex in the chosen ordering of points. This way each simplex σ in the V-R complex will be generated exactly once, when considering the local graph of its lowest vertex in the considered ordering.

Algorithm 1, that uses this idea, describes a way to list all the simplices of increasing dimension. At each iteration we obtain a list of d-dimensional simplices (given as collections of vertices) and, for each of them, a list of common subsequent neighbours of its vertices. We can then extend each simplex to a $(d+1)$-dimensional one by adding one common neighbour to the collections of vertices. When doing so we need to update the simplex's filtration value if one of the newly added edges is longer than the current filtration. Moreover, we need to update the list of common subsequent neighbours by intersecting it with the subsequent neighbours of the newly added vertex. Once we have obtained all possible $(d+1)$-simplices, we carry out this extension procedure one dimension higher. All of these operations are performed at the local graph of each vertex. The procedure ends when no simplex can be extended, i.e. when all maximal simplices have been listed. This construction might be understood as a breadth-first traversal of the simplex tree [27].

The main advantages of the proposed algorithm are two: it does not require to construct the whole complex, leading to a significant decrease in memory utilization; it considers each point separately, allowing the computations to be carried out independently.

The inputs of our algorithm are X, a ordered list of points in $\mathbb{R}^n$ and a maximum filtration value ϵ. The output is LIST_OF_CONTRIBUTIONS, an ordered list of pairs. For each simplex σ we store its contribution as a tuple $(f(\sigma), (-1)^{dim(\sigma)})$. The output list will sorted according to the filtration values.

Note that the Algorithm 1 is correct. Firstly, every simplex in the Vietoris-Rips complex will be generated. It will happen when its smallest vertex in the considered order will be considered in the *for* loop. Secondly, each simplex will be generated only once in the *INCREASE_DIMENSION* procedure. A simplex $\sigma = [v_0, \ldots, n_{n-1}, v_n]$, where $v_0 < \ldots < n_{n-1} < v_n$ will be generated from a simplex $[v_0, \ldots, n_{n-1}]$ by adding v_n as a common neighbour of its vertices.

Time performance

The worst case scenario occurs when the the 1-skeleton graph is fully connected. Assuming the point cloud consist of n points the resulting V-R complex will contain $2^n - 1$ simplices. In this case the time complexity of Algorithm 1 is $\mathcal{O}(2^{n-1}n)$. More details are provided in Appendix 14.

2 The points can be ordered in an arbitrary way.

Algorithm 1: COMPUTE LOCAL CONTRIBUTIONS V-R

Input: Ordered point cloud X, $\epsilon > 0$
Output: A ordered list of pairs (filtration, ± 1)

```
Create an empty vector C
for every point x_i in X do
    create the local graph G_i of subsequent neighbours of x_i;
    simplices = [x_i] ;
    filtrations = [0] ;
    common_subseq_neighs = [[subseq_neigh(G_i, x_i)]] ;
    while simplices NOT empty do
        for every simplex σ ∈ simplices do
            add to C the tuple (filtration(σ), (−1)^dim(σ));
        end
        INCREASE_DIMENSION(G_i, simplices,
          common_subseq_neighs) ;
    end
end
sort C according to the filtration value ;
return C
```

Algorithm 2: INCREASE_DIMENSION

Input: local graph G_i, simplices, common_subseq_neighs

```
new_simplices = [] ;
new_filtrations = [] ;
new_common_subseq_neighs = [] ;
for every simplex σ ∈ simplices do
    for every n ∈ common_subseq_neighs[σ] do
        new_simplices.append(σ+[n]);
        consider all the edges from vertices of σ to n and take
          the longest one ;
        new_f = MAX( filtration(σ) , length longest edge ) ;
        new_filtrations.append( new_f ) ;
        compute the intersection between the current
          common subsequent neighbours of σ and the
          subsequent neighbours of n in G_i ;
        new_common_subseq_neighs.append( intersection )
          ;
    end
end
simplices = new_simplices ;
filtrations = new_filtrations ;
common_subseq_neighs = new_common_subseq_neighs ;
```

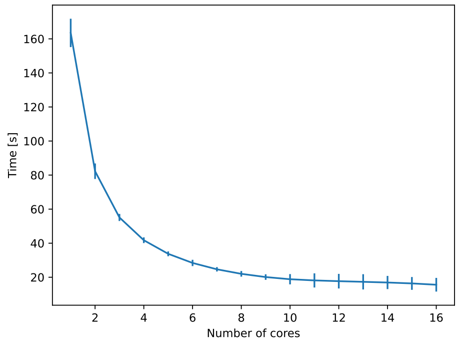

Figure 6. Average runtime over 10 runs of Algorithm 1 as a function of the number of cores used. Contributions computed for the V-R complex obtained from 10000 points sampled from the unit 4-sphere up to a maximum radius of 0.4. Experiment run on a AMD Ryzen Threadripper PRO 5955WX cpu. Error bars are scaled up by a factor of 20 for visibility.

Memory performance

Assuming the worst case scenario, the size of the output list of contributions is $O(n^2)$ while the maximal memory required at one intermediate step is $O(2^n/\sqrt{n})$. More details are provided in Appendix 14.

Choice of the vertex ordering

Note that the total running time of the fully parallelized Algorithm 1 can be dominated by few vertices whose simplex tree is considerably larger than the others. This explains the plateau in Figure 6. This effect can be mitigated by choosing a different ordering of the vertices. One efficient choice is to order the vertices by increasing number of ϵ-neighbours. Since the local graph for each vertex is constructed by considering only its subsequent neighbours, this ordering will produce more evenly-sized simplex trees. A simple example is showed in Figure 7 while the effect of this reshuffling on a larger dataset is shown in Figure 8.

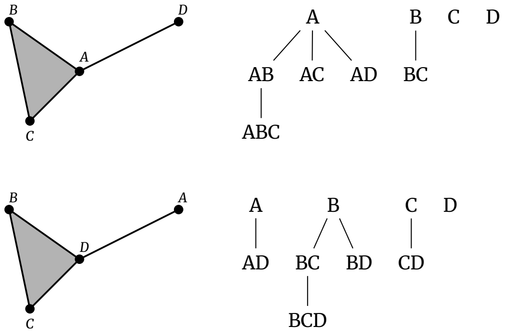

Figure 7. Different ordering of the vertices can produce different simplex trees. In the first row vertices are ordered by decreasing number of neighbours, in the second row by increasing number. The second choice produces more evenly-sized trees.

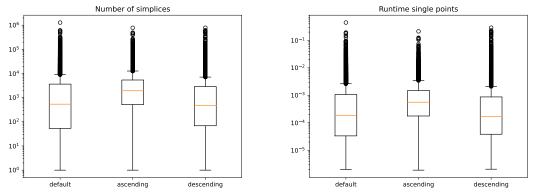

(a) **(b)**

Figure 8. Effect of different orderings of vertices for the example in Figure 6. Selecting the ascending order allows to achieve a more even distribution in the number of simplices in each tree (panel A) thus reducing the number of very large trees which dominate the running time (panel B).

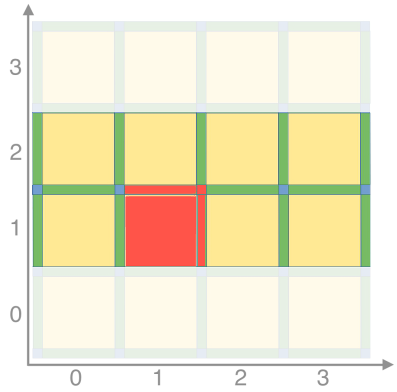

Figure 9. A slice of a cubical complex obtained from a 2 dimensional image. The image's pixels are associated to the top dimensional cells, depicted in yellow. Algorithm 3 takes as input a two voxel tick slice of the image and iterates through the voxels in the bottom row. At each iteration a voxels is selected and the contributions of the cells in its upper closure are computed. In this example, the voxel at coordinates (1, 1) is selected and the considered contributions are depicted in red: the one coming from the corresponding 2-cell, the two from the 1-cells shared with (2,1) and (1,2) and the contribution from the 0-cell shared with (2,1) , (1,2) and (2,2).

Cubical complexes

Cubical complexes are the most used combinatorial structure to represent digital grayscale images and extract topological information from them. There are two ways to construct a cubical complex from an image, the V-construction and the T-construction. The former identifies pixels - also know as voxels, in case of images of arbitrary dimension - with the vertices (the 0-dimensional cells) of the cubical complex. Voxels's values are used to define the filtration on the vertices and the filtration of each other elementary cube is the maximal value of its vertices. The T-construction can be seen as the dual procedure, voxels's values are assigned to the top dimensional cubes and the filtration values are propagated to lower dimensional cells by taking the minimum over the cofaces. The relation between these two constructions is explored in a recent work by Bleile et al. [28]. In this paper we choose the T-construction, although the presented techniques translate easily to the V-construction.

Similar to the V-R case, we are interested, given a grayscale n-dimensional image, in obtaining a list of contributions to the Euler characteristic of its corresponding cubical complex. As before, we then need to iterate over all cells σ in the complex and store each contribution as a tuple $(f(\sigma), (-1)^{dim(\sigma)})$. This can be achieved in a streaming fashion by loading into memory a two voxel high slice of the image, iterating through the cells in the bottom row computing their contributions, and then moving the sliding window up by one voxel. To make sure we consider each cells contribution exactly once, at each iteration we consider one voxel and compute the contributions to the Euler characteristic of the cells in its *upper closure*. Assuming that we can identify each top dimensional cell c_i with the indices $(x_1, \cdots, x_n)$ of the corresponding voxel in the input n-dimensional image, we define the upper closure of c_i as the set containing c_i and all its faces that are shared with other top dimensional cells c_j whose indices are $y_i = x_i$ or $y_i = x_i + 1$ for all i. An example of this procedure can be found in Figure 9.

Algorithm 3: COMPUTE LOCAL CONTRIBUTIONS CUBICAL

Input: A two voxels tick slice of an image, padded with $+\infty$
Output: A ordered list of pairs (filtration, ± 1)

```
Create an empty vector C
for every voxel c_i in the bottom row do
    for every cell σ in the upper closure of c_i do
        add to C the tuple (filtration(σ), (−1)^dim(σ))
    end
end
sort C according to the filtration value
return C
```

As already mentioned, a similar streaming algorithm to compute the ECC of grayscale images has been presented by Heiss and Wagner [15]. They also provide a fast open-source C++ implementation at https://bitbucket.org/hubwag/chunkyeuler. Recently Wang, Wagner and Chen [16] provided a GPU implementation of the same algorithm available at https://github.com/TopoXLab/GPU_ECC_SoCG2022. However, there is a significant difference between their approach and the one we describe in Algorithm 3: they keep track of the faces introduced by each voxel by looking at the gray values of the voxel's $3^d - 1$ neighbor and store the *cumulative* change in the EC at the voxel's filtration value. This requires sorting the voxel's value from the lowest to highest. While such a sorting can be done for one-value filtrations, it cannot be performed for multifiltrations, as no good ordering exists in the general case. There are some small differences in the implementation too: CHUNKYEuler only works with integer filtration values and only accepts 'raw' binary files as input. Our implementation, while being not as fast as CHUNKYEuler, offers the user more flexibility in the input and choice of filtration (or multifiltration) values.

Time and memory complexity

Considering a d-dimensional image with n voxels as input, the resulting cubical complex will have $3^d n$ cells. The running time of Algorithm 3 is then linear in the number of cells in the complex with a multiplicative constant which is exponential in the dimension. This is not a problem in practice as images with dimension larger than 3 are not common in applications. The memory requirement is just the space needed to store a two rows slice of the input image, the memory overhead for computing the local contributions for each voxel is negligible.

From Euler Characteristic Curves to Profiles

Both Algorithm 1 and Algorithm 3 can be immediately extended to compute the Euler Characteristic Profile of multifiltered Vietoris-Rips or cubical complexes. In the Vietoris-Rips case we require that all filtration functions should be defined on the vertices or the edges and then be extended to higher dimensional simplices by some user defined rule. This is to assure that the resulting multifiltered V-R complex is still a flag complex. In the case of cubical complexes we assume that the input images contains a $n-$tuple of numbers in each voxel - RGB images are a typical $n = 3$ example - and values are propagated to lower dimensional cells by some user defined rules. In both cases the output of both algorithms will be a list of $(n+1)-$tuples $(f_1(\sigma), \cdots, f_n(\sigma), (-1)^{dim(\sigma)})$ that stores the list of

contributions to the ECP at different points $f(\sigma) \in \mathbb{R}^n$.

Remark 7. In above, the simplest case of so called 1-critical multi-filtration is discussed. In this case, each cell σ appear in a unique value of the multifiltration. In a general case, a cell σ may appear in multiple non-comparable values $p_1, \dots, p_k$ of multifiltration. A simple generalization described below allows to adopt this presented algorithm to the general case; Let us assume that each p_i is n dimensional tuple, $p_i = (p_i^0, p_i^1, \dots, p_i^n)$. We assume that p_i and p_j are not comparable provided $i \neq j$. It means that there exist a pair of coordinates $l \neq m$ so that $p_i^l < p_j^l$ and $p_i^m > p_j^m$. Then, the cell σ contributes the value $(-1)^{\dim(\sigma)}$ for all the points $x \in \mathbb{R}^n$ for which there exist i such that $x > p_i$. Note that the regions consisting of points greater that p_i overlap for different $i \in \{1, \dots, k\}$, hence we need to avoid double and multiple counting of the contributions. Below we describe a procedure to achieve it and enforce the contribution of exactly $(-1)^{\dim(\sigma)}$ for all $x > p_i$ for arbitrary $i \in \{1, \dots, k\}$. For that purpose, given $i \neq j$, we define $p_i \vee p_j = (\max(p_i^1, p_j^1), \max(p_i^2, p_j^2), \dots, \max(p_i^n, p_j^n))$. Algorithm 4 define a set of points with appropriate contributions to enforce the required condition for all $x \geq p_i$ for all $i \in \{1, \dots, k\}$.

Algorithm 4: CONTIBUTION OF σ TO ECP

Input: d - dimension of s, $p_1, \dots, p_k \in \mathbb{R}^n$ - incompatible times of appearance of σ in the multifiltration
Output: A collection of contributions of σ to ECP

1 List *Contribution* $\leftarrow (p_i, (-1)^d)$, for $i \in \{1, \dots, k\}$
2 $P = p_i \vee p_j$ for every $i, j \in \{1, \dots, k\}$
3 Queue $L \leftarrow p_i \vee p_j$ for $i \neq j \in \{1, \dots, k\}$
4 **while** $L \neq \emptyset$ **do**
5   p = dequeue(L)
6   P' = all elements $p' \in P$ such that $p' < p$
7   **if** *all elements P' are already in Contribution* **then**
8   c = sum of values of elements in P' in *Contribution*
9   *Contribution* $\leftarrow (p, (-1)^d - c)$
10   **else**
11   L = enqueue(p)
12   **end**
13 **end**
14 **return** *Contribution*

It is straightforward to see that for any given cell σ, its contributions to the ECP will change at at most in $p_i \vee p_j$ for $i, j \in \{1, \dots, k\}$, where $\{p_1, \dots, p_k\}$ are incompatible points in which σ appears in the multifiltration. Algorithm 4 scans all those points, and assigns the appropriate value (see line 1 and 9) to contributions to the ECP. Note that all points $p_1, \dots, p_k$ have their contributions initially set in the line 1. Consequently, the presented algorithm will terminate, as in each iteration at least one p will be added to the *Contribution* list. In addition, it explicitly enforces the correct contribution of the cell σ to all points $x \geq p_i$ for any $i \in \{1, \dots, k\}$.

Data Structures for ECPs

All the algorithms we described in the previous section output a list of contributions to the Euler Characteristic Profile. For a n-dimensional profile, each contribution in the list is a pair where the first entry is a n-tuple storing the coordinates in $\mathbb{R}^n$ at which the Euler characteristic varies by the integer values stored in the second item. When dealing with one dimensional ECCs it makes sense to sort the contributions according to their filtration value, in order to perform faster operations on them.

Retrieving the EC at some filtration values

Given a ECP as a list of contributions, the first basic operation is to retrieve the value of the Euler characteristic at an arbitrary filtration value f_*. It can be obtained by summing up all the contributions in the ECP that appear at filtration values less or equal f_*. For a d-dimensional ECP this can be achieved in linear time with respect to the size of the contribution list. In the one dimensional case, we can take advantage of the total ordering on the list of contributions, since the filtration values $f_i \in \mathbb{R}$. By doing so we can build an auxiliary data structure storing the value of the Euler characteristic at each f_i, the points in which the ECC is changing value. This can be done in $O(n)$ time and space, where n is the length of the list of contributions. Given such a structure, computing the value of the ECC at a given filtration f_* boils down to the the search for the largest jump point $f_i < f_*$ and retrieving the value of the ECC therein. This can be achieved by interpolation search in $O(log(log(n)))$ time.

Computing distances

Distances between Euler Characteristic Curves

In Section Euler Characteristic Curves we introduced the notion of difference between two ECCs, expressed in terms of the L_1 norm of the difference between the two curves. One should note that, in the case of finite Vietoris-Rips or cubical complexes, such a difference is always finite (but not bounded) as all ECCs will eventually stabilize to 1 for a sufficiently large filtration value. In case when the construction of a Vietoris-Rips complexes is stopped at a certain diameter 2ϵ, and the final complexes have more than one infinite homology, it make sense to restrict the integral used in distance computations to an interval $[0, 2\epsilon]$ in order to make the distances between the ECCs finite.

Both Algorithm 1 and 3 return the computed ECC as list of pairs (f_i, c_i) where c_i is an integer representing the change in the Euler characteristic at filtration f_i. Such list is sorted in increasing order with respect to the filtration values. Using such data structure the difference between two ECCs can be computed in linear time with the size of the lists. Given two list of contributions ECC_1 and ECC_2 we can merge them in linear time, preserving the order. While merging we flip the sign of all the contributions coming from ECC_2. Let us denote the obtained list with ECC_{1-2}. Now the difference can be computed by iterating over the full list

$$||ECC_1 - ECC_2||_1 = \sum_i (f_{i+1} - f_i) EC(f_i) \quad ,$$

where $EC(f_i) = \sum_{j=0}^{i} c_j$ with respect to the ordering of ECC_{1-2}.

Distances between Euler Characteristic Profiles

Unfortunately the strategy proposed in the previous section is difficult to generalize in the multifiltration setting as there is no natural way to sort the list of contributions. We present here a basic algorithm to compute the distances between two ECPs and leave the search for potentially faster algorithm to future work.

Let ECP_1 and ECP_2 be two list of contributions representing two n-dimensional profiles. We can merge them in linear time, as in the one dimensional case, flipping the sign of the contributions in the second list. Let N be the total number of contributions. With reference to Figure 10, the coordinates of such contributions will create a n-dimensional irregular grid of size $(N+1)$. The value of the EC inside each cuboid will be equal to the EC at the cuboid's bottom left corner and can be computed in $O(N)$. The L_1 distance between the two ECPs can then be obtained by summing up the values of the EC in each cuboid weighted by the cuboid's volume. Given that the number of cuboids is $(N+1)^d$, this operation can be computed in $O(N^{d+1})$. Note that the ECPs need to be truncated in order to avoid cuboids with infinite volume.

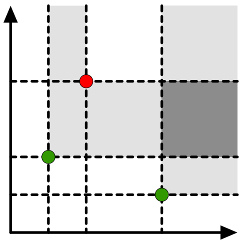

Figure 10. Example of a two dimensional ECP with three contributions. The green points indicate a +1 while the red point is a −1. The plane can then be subdivided in a 4×4 irregular grid. The coloring of each block indicates the value of the EC in that that region, white is 0, light gray is 1 and dark gray is 2.

Vectorization

Vectorizing the ECC / ECP is a critical step if we are interested in using these invariants in a Machine Learning framework.

Curves

Assume we are given an ECC whose filtration values ranges from 0 to f_{max}. We can convert it to a vector by evenly sampling it N times between 0 and f_{max}. If we chose to include the endpoints the resulting vector will be $vec(ECC, N) = [EC(0), EC(\Delta), EC(2\Delta), \cdots, EC((N-2)\Delta), EC(f_{max})]$, where Δ is the vectorization's resolution which is defined as $\Delta = f_{max}/(N-1)$.

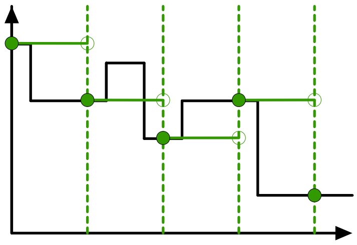

Figure 11. An Euler Characteristic Curve (black) and its vectorized version (green) with resolution Δ. In this case, the vectorized version is stored as a vector of length 5 (the green filled-in points), but can be reconverted to a stepsize function.

The vectorized ECC can be obtained by such vector as the union of $N-1$ left-closed, right-open intervals of length Δ that correspond to to sampling the value of the EC at filtration value f_i and extending it till f_{i+1}. It makes sense then to ask whether it is possible to bound the difference between an ECC and its vectorized representation. Figure 11 is an example of such difference when a curve is sampled in 5 points.

Proposition 5. *Let K be a filtered cell complex whose filtration values ranges from 0 to f_{max}. The L_1 norm between the Euler Characteristic Curve of K and its vectorized version at resolution Δ is bounded by*

$$||ECC(K) - vec(ECC(K), N)||_1 \leq \Delta(|K|/2 + F) \qquad (3)$$

where $|K|$ is the number of simplices in the complex and $F = \sum_{i=0}^{n-2} |EC(i\Delta) - EC((i+1)\Delta)|$ is the sum of the absolute value of the differences between consecutive values in the vectorized Euler Characteristic $vec(ECC(K), N)$.

Proof. We will prove the two terms in the bound separately as they come from two different types of errors.

Type I errors occur when the EC at two consecutive sampling points f_i and f_{i+1} is different. The simplest case is depicted in Figure 12A, the EC changes values in between the sampling interval. We can upper bound this error with the area of the rectangle having as base the vectorization's resolution $\Delta = f_{i+1} - f_i$, and as height the difference between the EC at the two sampling points $|EC(f_i) - EC(f_{i+1})$. Note that this bound also holds in the more general case where the EC varies monotonically at multiple values inside the sampling interval. By summing up all the contributions we obtain the value $\Delta \cdot F = \Delta \cdot \sum_{i=0}^{n-2} |EC(i\Delta) - EC((i+1)\Delta)|$.

Type II errors, see Figure 12B, occur when the EC has the same value at consecutive filtration steps but varies in between. The maximum possible variation can be upper bounded by the area of the rectangle with Δ as base and the half the number of cells in the complex as height. Each cell contributes to the EC by ± 1, the factor one half is due to the constrain that the EC has the same value in f_i and f_{i+1}. This amounts to the values $\Delta \cdot |K|/2$.

By summing up the two contributions we obtain the bound in 3. Note that a generic situation can always be described as a combination of type I and type II errors.

□

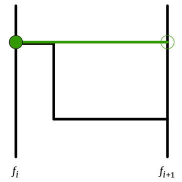

(a) Type I

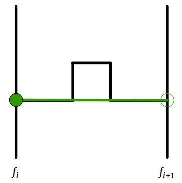

(b) Type II

Figure 12. The two possible source of errors during vectorization of an ECC

We have shown a way to bound the distance between an ECC and its vectorized version. Another possible stability question is whether this vectorization preserves distances between ECCs. In other words, we are interest in knowing whether something can be said for $||vec(ECC_1, N) - vec(ECC_2, N)||$ given $||ECC_1 - ECC_2||$ Unfortunately it is possible to construct examples in which two curves can be made arbitrary far apart but they have the same vectorization or two curves can be made arbitrary close but they have drastically different vectorizations. Figure 13 shows two of such examples. Moreover, in the existing literature Johnson and Jung [29] prove that the distance between two vectorized Betti curves can not be bounded by the Wasserstein distance between the respective persistence diagrams. They propose a stable vectorization inspired by Gaussian smoothing techniques.

Profiles

An n-dimensional Euler Characteristic Profile whose filtration values ranges from 0 to f^i_{max} for $i \in 1 \cdots n$ can be vectorized in a similar fashion by sampling it on a grid of size $N_1 \times N_2 \times \cdots \times N_n$. In general the N_i can be different and thus leading to different resolutions Δ_i on the various filtration parameters. The output of this sampling procedure is a n-dimensional tensor $vec(ECP, N_i)$ that can be eventually flattened to a 1-dimensional vector. Although this is an intuitive generalization of the 1-dimensional ECC case, the procedure has an increased computational cost due to the difficulties in sampling EC values from a profile, as already discussed in Section 14. Moreover, the stability result in 5 can not be generalized to the multiparameter setting. As depicted in Figure 14, the grid

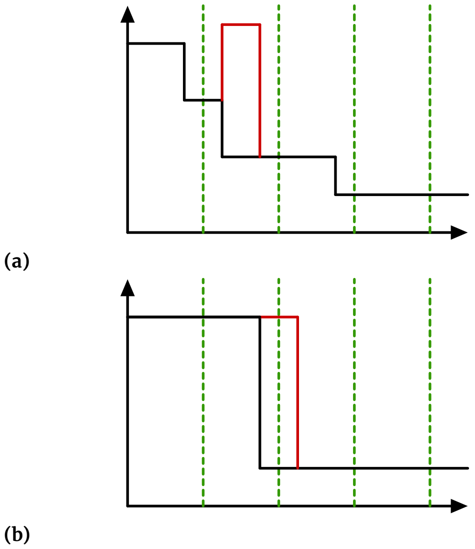

Figure 13. Two ECC superimposed in the same plot. In panel (A) the two curves can be made arbitrary far apart in L_1 but they have the same vectorization. In panel (B) the two curves can be made arbitrary close but they have drastically different vectorizations.

vectorization could be not able to detect the contributions coming from pairs of cells. In the multiparameter case however, it is not possible to bound this contributions using only the vectorization resolutions Δ as such contribution can persist on subsequent grid elements up to infinity.

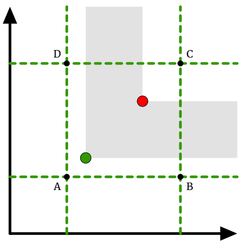

Figure 14. A 2-dimensional analog of a type II error of Figure 12. The ECP is vectorized by sampling the EC values on the green grid. We can add pair of cells with contributions ± 1 inside the rectangle ABCD in such a way that the value of the EC on the vertices does not change. However, such contributions have a non-zero sum on an area that can be made arbitrary large.

Examples and Experiments

RGB images

A toy experiment using 3 dimensional Euler Characteristic Profiles can be constructed using RGB images. In a RGB image each pixel contains a tuple of 3 integers, each ranging from 0 to 255. They stand for the Red, Green and Blue color channel and all colors in the visible spectrum can be represented by a 3 tuple. In particular black is coded by (0,0,0) and white is (255, 255, 255).

In this example we consider two different textures, stripes and checks, each of them can be red, green or blue. We generate 10 samples of each combination of style and color by adding random Gaussian noise to each pixel. We then compute the 3 dimensional Euler Characteristic Profile of the cubical complex obtained from each image and computed the matrix of pairwise L_1 distances between them. Such matrix is show in Figure 15. It confirms that distance between Euler Characteristic Profiles of different images increase following the intuitive sequence 'same style, same color' < 'same style, different color' < 'different style, same color' < different style, different color'.

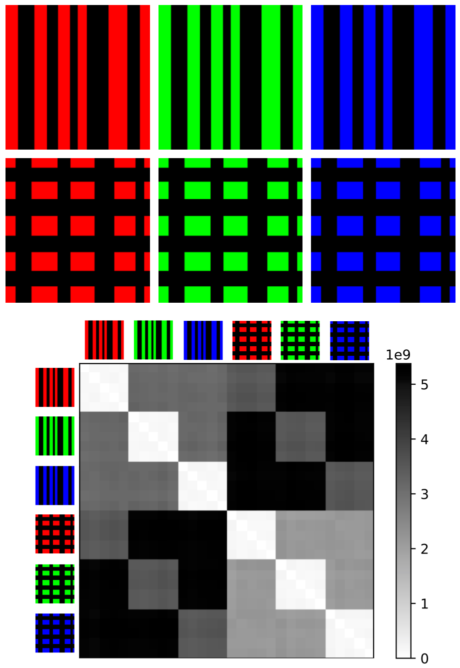

Figure 15. 60 $\times$ 60 distance matrix between Euler Characteristic Profiles of different RGB images.

Immune cell spatial patterns in tumors

Vipond et al. [30] applied multiparameter persistent homology (MPH) landscapes to study immune cell location in digital histology images from head and neck cancer. They extracted the locations of three immune cell types from histology slides thus obtaining a list of pointclouds labelled CD8+, FoxP3+, or CD68. The goal is to correctly classify a pointcloud. All pointcloud data are available at `github.com/MultiparameterTDAHistology/SpatialPatterningOfImmuneCells`.
The Authors created a bifiltered Vietoris-Rips complex from each pointcloud, using radius and a codensity function defined over each vertex p as $\rho_{10}(p) = \frac{1}{10}\sum_{i=1}^{10} ||p - p_i||_2$ where p_i is the i-th nearest neighbor of p. They then computed MPH-landscapes and used them as input for one of three classifiers: Linear Discriminant Analysis (LDA), Regularised Linear Discriminant Analysis (rLDA), and regularised Quadratic Discriminant Analysis (rQDA) [31]. They made a randomized 80/20 training/test split, and evaluate the classification accuracy of 3 classifiers on the test data for each pair of cell types and for the three-class problem. The classification results are reported in the supplementary material of [30].

We used the Authors' code to re-generate the same standard Vietoris-Rips and bifiltered Vietoris-Rips complexes from the provided pointclouds. We then computed ECC (radius only) and ECP (radius and codensity) for each complex and used them as input for the same LDA, rLDA and rQDA classifiers using the same train-test split procedure. The average accuracy for the various classification task are reported in Tables 1, 2 and 3. Both ECC and ECP significatively outperforms MPH-landscapes while there is apparently no gain in moving from ECC to ECP. This can be an indication that the second dimension in the filtration (the codensity parameter) does

	CD68+ vs FoxP3+	CD8+ vs FoxP3+	CD8+ vs CD68+	CD8+ vs CD68+ vs FoxP3+
	MPL - ECC - ECP	MPL - ECC - ECP	MPL - ECC - ECP	MPL - ECC - ECP
T_A	0.584 - 0.938 - **0.941**	0.672 - **0.994** - 0.988	0.669 - **0.894** - 0.856	0.486 - **0.896** - 0.886
T_B	0.794 - 0.917 - **0.922**	0.88 - **0.992** - **0.992**	0.54 - 0.943 - **0.962**	0.568 - 0.921 - **0.940**
T_C	0.723 - **0.947** - 0.904	0.7 - **0.884** - 0.859	0.605 - **0.811** - 0.699	0.505 - **0.842** - 0.755
T_D	0.811 - **0.960** - 0.933	0.899 - **0.986** - 0.985	0.644 - 0.802 - **0.807**	0.613 - 0.862 - **0.874**
T_E	0.732 - **0.941** - 0.940	0.644 - 0.867 - **0.869**	0.593 - **0.806** - 0.688	0.511 - **0.842** - 0.719
T_F	0.738 - 0.655 - **0.933**	0.644 - 0.619 - **0.830**	0.73 - 0.709 - **0.850**	0.511 - 0.578 - **0.824**
T_G	0.771 - 0.788 - **0.858**	0.782 - 0.791 - **0.904**	**0.675** - 0.614 - 0.609	0.599 - **0.673** - 0.659
T_H	0.710 - 0.651 - **0.885**	0.682 - 0.747 - **0.955**	0.628 - 0.695 - **0.891**	0.555 - 0.659 - **0.845**
T_I	0.733 - **0.788** - 0.737	**0.758** - 0.716 - 0.679	0.540 - 0.693 - **0.713**	0.548 - **0.716** - 0.493
T_J	0.727 - 0.642 - **0.767**	0.535 - 0.678 - **0.857**	0.602 - 0.808 - **0.868**	0.449 - 0.507 - **0.699**
T_K	0.510 - **0.872** - 0.770	0.570 - 0.784 - **0.816**	0.502 - 0.823 - **0.877**	0.404 - 0.594 - **0.635**
T_N	0.493 - 0.457 - **0.570**	0.512 - **0.658** - 0.632	0.577 - 0.507 - **0.760**	0.342 - **0.462** - 0.370
T_O	**0.948** - 0.830 - 0.840	**0.788** - 0.602 - 0.754	0.532 - 0.484 - **0.598**	0.550 - 0.431 - **0.615**

Table 1. Average classification accuracy for the LDA classifier using as input MLP, ECC or ECP. Data for each tumor are split into 80/20 train-test splits and classification accuracy is reported as the mean over 100 repetitions of splitting, training and testing.

	CD68+ vs FoxP3+	CD8+ vs FoxP3+	CD8+ vs CD68+	CD8+ vs CD68+ vs FoxP3+
	MPL - ECC - ECP	MPL - ECC - ECP	MPL - ECC - ECP	MPL - ECC - ECP
T_A	0.491 - **0.967** - 0.964	0.642 - **0.973** - 0.967	0.630 - **0.840** - 0.830	0.427 - 0.858 - **0.859**
T_B	0.760 - **0.892** - 0.869	0.787 - **0.986** - 0.985	0.671 - 0.942 - **0.945**	0.604 - **0.868** - 0.865
T_C	0.863 - **0.906** - 0.896	0.747 - **0.847** - 0.842	**0.653** - 0.584 - 0.614	**0.640** - 0.628 - 0.627
T_D	0.683 - **0.926** - 0.918	0.829 - **0.990** - 0.988	0.476 - **0.779** - **0.779**	0.492 - **0.779** - 0.775
T_E	0.820 - **0.886** - 0.883	0.736 - **0.929** - 0.920	0.534 - 0.735 - **0.743**	0.502 - **0.702** - 0.683
T_F	0.623 - 0.899 - **0.925**	0.476 - 0.842 - **0.847**	0.765 - 0.909 - **0.921**	0.408 - 0.845 - **0.847**
T_G	0.886 - **0.932** - 0.927	0.897 - 0.970 - **0.975**	0.446 - **0.696** - 0.692	0.581 - 0.738 - **0.746**
T_H	0.524 - 0.890 - **0.898**	0.735 - **0.930** - 0.929	0.714 - **0.882** - 0.877	0.502 - 0.844 - **0.859**
T_I	0.859 - 0.912 - **0.931**	0.883 - 0.908 - **0.909**	0.484 - 0.470 - **0.474**	0.597 - **0.619** - 0.614
T_J	0.608 - **0.763** - 0.750	0.750 - 0.835 - **0.872**	0.850 - 0.882 - **0.892**	0.536 - 0.653 - **0.670**
T_K	0.376 - **0.868** - 0.804	0.523 - **0.918** - 0.914	0.455 - **0.857** - 0.845	0.261 - **0.718** - 0.679
T_N	0.410 - 0.527 - **0.563**	0.432 - 0.662 - **0.745**	0.643 - 0.690 - **0.713**	0.294 - 0.388 - **0.460**
T_O	0.702 - **0.954** - 0.952	0.644 - **0.806** - 0.772	0.546 - 0.672 - **0.684**	0.429 - **0.639** - 0.632

Table 2. Average classification accuracy for the rLDA classifier using as input MLP, ECC or ECP. Data for each tumor are split into 80/20 train-test splits and classification accuracy is reported as the mean over 100 repetitions of splitting, training and testing.

not contain significant information.

Prostate cancer histology slides

Lawson et al. [32] demonstrated that Persistent Homology can successfully be used to evaluate features in prostate cancer hematoxylin and eosin (H&E) stained slides. Their dataset, available in the Open Science Framework `https://osf.io/k96qw/` contains 5182 RGB images of a resolution 512 $\times$ 512 corresponding to different regions of interest (ROIs) in prostate cancer H&E slices obtained from 39 patients. Each image is labelled with a Gleason score of 3, 4 or 5 indicating the architectural patterns of the cancer. An higher Gleason score indicates an increasing level of cancer aggressiveness. The datasets contains 2567 grade 3 ROIs, 2351 grade 4 ROIs but only 264 grade 5 ROIs. Given the unbalance in the data we decided to consider a classification problem between grade 3 and 4.

Following the procedure described by the Authors we normalized and extracted the hematoxylin and eosin color channel from each ROI. By doing so we converted each RGB image into a bidimensional (H, E) one. We first computed the ECC for each of the grayscale images corresponding to the hematoxylin channel as it is the color that highlights cell nuclei. We then also used the eosin color channel to obtain a 2-dimensional ECP. We input either the ECCs or the ECPs into an Support Vector Machine (SVM) [33] classifier and computed the mean test accuracy over 100 rounds with a 80/20 training split. The results are displayed in Table 4. The classifier using as input the 2-dimensional ECPs is consistently performing better than the one using the 1-dimensional ECCs.

Conclusions

Euler Characteristic Curves and Profiles provide a stable summary of the shape of data. Unlike other summaries used in Topological Data Analysis this one can be computed in a distributed fashion, hence is applicable to deal with big data problems. In addition we show, contrary to a common misconception, that the Euler Characteristic Curves and Profiles enjoys certain type of stability. We confirm it when using them to discriminate various toy datasets with varying level of noise. We also show how to compare and vectorize the Euler Characteristic Curves and Profiles and apply them to a number of real data analysis problems. The presented results are accompanied with efficient Python implementation. For example, on modern commodity hardware, our implementation for V-R complexes can handle a number of simplices on the order of 10^{10}. This is two order of magnitude more that what can be achieved using available software like GUDHI [9]. With this work we hope that the machinery of Euler Characteristic Curves and Profiles will be useful for practitioners in Topological Data Analysis.

Code availability

Python implementations of Algorithms 1 and 3 are available at `https://github.com/dioscuri-tda/pyEulerCurves` and can be identified by biotools:pyeulercurves or RRID:SCR_024472. Jupyter notebooks to reproduce all the experiments described in this article are available at `https://github.com/dioscuri-tda/ecp_experiments` with DOI 10.48546/workflowhub.workflow.576.1.

	CD68+ vs FoxP3+	CD8+ vs FoxP3+	CD8+ vs CD68+	CD8+ vs CD68+ vs FoxP3+
	MPL - ECC - ECP	MPL - ECC - ECP	MPL - ECC - ECP	MPL - ECC - ECP
T_A	0.503 - **0.945** - 0.931	0.598 - **0.840** - 0.838	0.598 - **0.840** - 0.838	0.380 - **0.865** - 0.861
T_B	0.738 - **0.896** - 0.867	0.588 - **0.913** - 0.911	0.588 - **0.913** - 0.911	0.531 - **0.871** - 0.869
T_C	0.855 - **0.915** - 0.906	0.673 - 0.552 - **0.568**	0.673 - 0.552 - **0.568**	0.614 - 0.627 - **0.640**
T_D	0.554 - **0.934** - 0.929	0.494 - **0.767** - 0.755	0.494 - **0.767** - 0.755	0.482 - 0.786 - **0.787**
T_E	0.826 - **0.876** - 0.871	0.548 - 0.751 - **0.754**	0.548 - 0.751 - **0.754**	0.499 - **0.754** - 0.724
T_F	0.646 - **0.964** - 0.963	0.666 - 0.853 - **0.855**	0.666 - 0.853 - **0.855**	0.412 - **0.881** - 0.878
T_G	0.882 - **0.937** - 0.928	0.485 - **0.723** - 0.699	0.485 - **0.723** - 0.699	0.583 - **0.771** - 0.767
T_H	0.621 - 0.968 - **0.967**	0.699 - 0.886 - **0.898**	0.699 - 0.886 - **0.898**	0.550 - 0.889 - **0.901**
T_I	0.919 - 0.928 - **0.940**	0.493 - **0.531** - 0.527	0.493 - **0.531** - 0.527	**0.626** - 0.621 - 0.624
T_J	0.588 - **0.908** - 0.903	0.860 - 0.898 - **0.902**	0.860 - 0.898 - **0.902**	0.541 - **0.720** - 0.719
T_K	0.468 - **0.874** - 0.838	0.567 - **0.923** - 0.903	0.567 - **0.923** - 0.903	0.352 - **0.751** - 0.736
T_N	0.353 - 0.453 - **0.477**	0.510 - **0.617** - 0.600	0.510 - **0.617** - 0.600	0.334 - **0.392** - 0.384
T_O	0.724 - 0.972 - **0.984**	0.524 - **0.668** - 0.662	0.524 - **0.668** - 0.662	0.440 - 0.730 - **0.738**

Table 3. Average classification accuracy for the rQDA classifier using as input MLP, ECC or ECP. Data for each tumor are split into 80/20 train-test splits and classification accuracy is reported as the mean over 100 repetitions of splitting, training and testing.

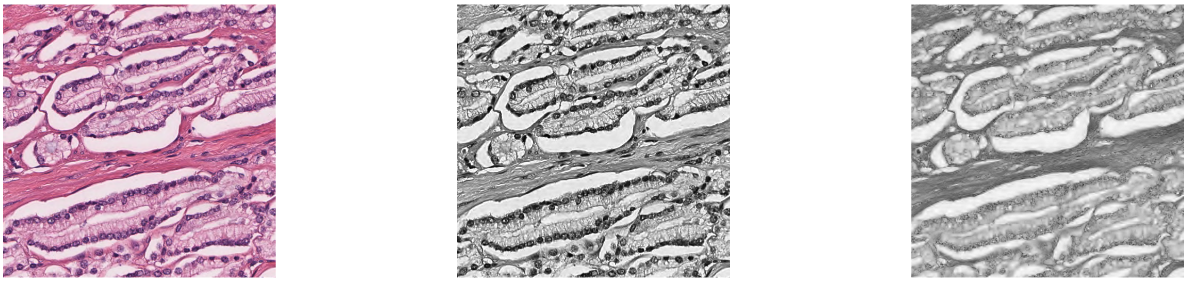

(a) (b) (c)

Figure 16. Panel (A) depicts a raw RGB ROI. Panel (B) contains the hematoxylin channel while panel (C) contains the eosin one.

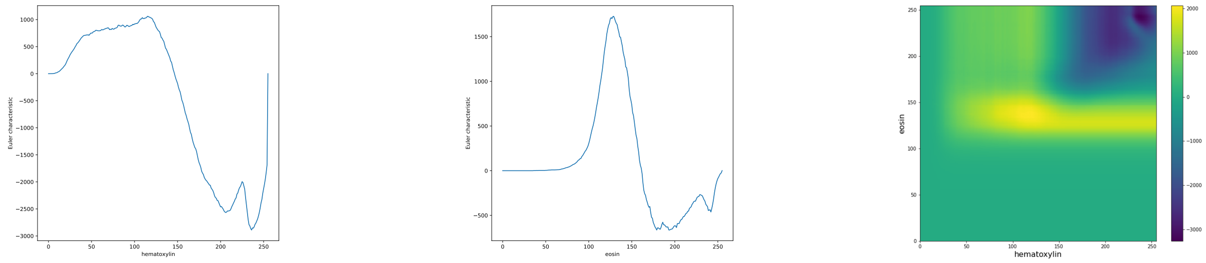

(a) (b) (c)

Figure 17. Panel (A) depicts the hematoxylin ECC for the ROI in Figure 16 while panel (B) depicts the eosin ECC. The combined ECP is showed in panel (C).

hematoxylin ECC	hematoxylin & eosin ECP
0.765 ± 0.001	0.826 ± 0.001

Table 4. Mean test accuracy for the Gleason 3 vs Gleason 4 classification using ECCs or ECPs as input to an SVM classifier.

Declarations and Acknowledgments

PD and DG acknowledge support by Dioscuri program initiated by the Max Planck Society, jointly managed with the National Science Centre (Poland), and mutually funded by the Polish Ministry of Science and Higher Education and the German Federal Ministry of Education and Research. DG is also with the University of Warsaw within the Doctoral School of Exact and Natural Sciences. The authors declare that they have no competing interests. DG thanks Niklas Hellmer for the multiple, valuable discussions and insights.

References

1. Edelsbrunner, Letscher, Zomorodian. Topological Persistence and Simplification. Discrete & Computational Geometry 2002 Nov;28(4):511–533. https://doi.org/10.1007/s00454-002-2885-2.
2. Singh G, Memoli F, Carlsson G. Topological Methods for the Analysis of High Dimensional Data Sets and 3D Object Recognition. The Eurographics Association; 2007. https://diglib.eg.org:443/xmlui/handle/10.2312/SPBG.SPBG07.091-100, accepted: 2014-01-29T16:52:11Z ISSN: 1811-7813.
3. Edelsbrunner H, Harer JL. Computational topology: an introduction. American Mathematical Society; 2022.
4. Lee Y, Barthel SD, Dłotko P, Moosavi SM, Hess K, Smit B. Quantifying similarity of pore-geometry in nanoporous materials. Nature Communications 2017 May;8(1):15396. https://www.nature.com/articles/ncomms15396, number: 1 Publisher: Nature Publishing Group.
5. Dłotko P, Wanner T. Topological microstructure analysis using persistence landscapes. Physica D: Nonlinear Phenom-

ena 2016 Nov;334:60–81. https://www.sciencedirect.com/science/article/pii/S0167278916301750.
6. Hiraoka Y, Nakamura T, Hirata A, Escolar EG, Matsue K, Nishiura Y. Hierarchical structures of amorphous solids characterized by persistent homology. Proceedings of the National Academy of Sciences 2016 Jun;113(26):7035–7040. https://www.pnas.org/doi/full/10.1073/pnas.1520877113, publisher: Proceedings of the National Academy of Sciences.
7. Nicolau M, Levine AJ, Carlsson G. Topology based data analysis identifies a subgroup of breast cancers with a unique mutational profile and excellent survival. Proceedings of the National Academy of Sciences 2011 Apr;108(17):7265–7270. https://www.pnas.org/doi/10.1073/pnas.1102826108, publisher: Proceedings of the National Academy of Sciences.
8. Bauer U, Kerber M, Reininghaus J. Distributed Computation of Persistent Homology. In: 2014 Proceedings of the Meeting on Algorithm Engineering and Experiments (ALENEX) Proceedings, Society for Industrial and Applied Mathematics; 2013.p. 31–38. https://epubs.siam.org/doi/abs/10.1137/1.9781611973198.4.
9. The GUDHI Project. GUDHI User and Reference Manual. 3.6.0 ed. GUDHI Editorial Board; 2022. https://gudhi.inria.fr/doc/3.6.0/.
10. Silva Vd, Carlsson G. Topological estimation using witness complexes. In: Gross M, Pfister H, Alexa M, Rusinkiewicz S, editors. SPBG'04 Symposium on Point - Based Graphics 2004 The Eurographics Association; 2004. .
11. Sheehy DR. Linear-Size Approximations to the Vietoris–Rips Filtration. Discrete & Computational Geometry 2013 Jun;49(4):778–796. https://doi.org/10.1007/s00454-013-9513-1.
12. Chazal F, Fasy BT, Lecci F, Rinaldo A, Singh A, Wasserman L. On the Bootstrap for Persistence Diagrams and Landscapes. Modeling and Analysis of Information Systems 2013 Dec;20(6):111–120. https://www.mais-journal.ru/jour/article/view/162, number: 6.
13. Carlsson G, de Silva V. Zigzag Persistence. Foundations of Computational Mathematics 2010 Aug;10(4):367–405. https://doi.org/10.1007/s10208-010-9066-0.
14. Pedregosa F, Varoquaux G, Gramfort A, Michel V, Thirion B, Grisel O, et al. Scikit-learn: Machine Learning in Python. Journal of Machine Learning Research 2011;12:2825–2830.
15. Heiss T, Wagner H. Streaming Algorithm for Euler Characteristic Curves of Multidimensional Images. In: Felsberg M, Heyden A, Krüger N, editors. Computer Analysis of Images and Patterns Lecture Notes in Computer Science, Cham: Springer International Publishing; 2017. p. 397–409.
16. Wang F, Wagner H, Chen C, GPU Computation of the Euler Characteristic Curve for Imaging Data. arXiv; 2022. http://arxiv.org/abs/2203.09087, arXiv:2203.09087 [cs].
17. Roy A, Haque RaI, Mitra AJ, Choudhury MD, Tarafdar S, Dutta T. Understanding flow features in drying droplets via Euler characteristic surfaces—A topological tool. Physics of Fluids 2020 Dec;32(12):123310. https://aip.scitation.org/doi/10.1063/5.0026807, publisher: American Institute of Physics.
18. Beltramo G, Skraba P, Andreeva R, Sarkar R, Giarratano Y, Bernabeu MO. Euler characteristic surfaces. Foundations of Data Science 2021;https://www.aimsciences.org/article/doi/10.3934/fods.2021027, company: Foundations of Data Science Distributor: Foundations of Data Science Institution: Foundations of Data Science Label: Foundations of Data Science Publisher: American Institute of Mathematical Sciences.
19. Chen Y, Segovia-Dominguez I, Coskunuzer B, Gel Y. TAMP-S2GCNets: Coupling Time-Aware Multipersistence Knowledge Representation with Spatio-Supra Graph Convolutional Networks for Time-Series Forecasting. In: International Conference on Learning Representations; 2022. https://openreview.net/forum?id=wv6g8fWLX2q.
20. Perez D, Euler and Betti curves are stable under Wasserstein deformations of distributions of stochastic processes. arXiv; 2022. http://arxiv.org/abs/2211.12384, arXiv:2211.12384 [math].
21. Hatcher A. Algebraic topology. Cambridge: Cambridge University Press; 2002.
22. Carlsson G, Zomorodian A. The Theory of Multidimensional Persistence. SCG '07 2007;.
23. Botnan MB, Lesnick M, An Introduction to Multiparameter Persistence. arXiv; 2022. http://arxiv.org/abs/2203.14289, arXiv:2203.14289 [cs, math].
24. Chung YM, Lawson A. Persistence Curves: A canonical framework for summarizing persistence diagrams. Advances in Computational Mathematics 2022 Jan;48(1):6. https://doi.org/10.1007/s10444-021-09893-4.
25. Chevyrev I, Nanda V, Oberhauser H. Persistence Paths and Signature Features in Topological Data Analysis. IEEE Transactions on Pattern Analysis and Machine Intelligence 2020 jan;42(1):192–202. https://doi.org/10.1109%2Ftpami.2018.2885516.
26. Roune BH, de Cabezón ES, Complexity and Algorithms for Euler Characteristic of Simplicial Complexes. arXiv; 2011. http://arxiv.org/abs/1112.4523, arXiv:1112.4523 [cs, math].
27. Boissonnat JD, Maria C. The Simplex Tree: An Efficient Data Structure for General Simplicial Complexes. Algorithmica 2014 Nov;70(3):406–427. https://doi.org/10.1007/s00453-014-9887-3.
28. Bleile B, Garin A, Heiss T, Maggs K, Robins V. The Persistent Homology of Dual Digital Image Constructions. In: Gasparovic E, Robins V, Turner K, editors. Research in Computational Topology 2 Association for Women in Mathematics Series, Cham: Springer International Publishing; 2022.p. 1–26. https://doi.org/10.1007/978-3-030-95519-9_1.
29. Johnson M, Jung JH, Instability of the Betti Sequence for Persistent Homology and a Stabilized Version of the Betti Sequence. arXiv; 2021. http://arxiv.org/abs/2109.09218, arXiv:2109.09218 [cs, math].
30. Vipond O, Bull JA, Macklin PS, Tillmann U, Pugh CW, Byrne HM, et al. Multiparameter persistent homology landscapes identify immune cell spatial patterns in tumors. Proceedings of the National Academy of Sciences 2021 Oct;118(41):e2102166118. https://pnas.org/doi/full/10.1073/pnas.2102166118.
31. Hastie T, Tibshirani R, Friedman J. The Elements of Statistical Learning. Springer Series in Statistics, New York, NY: Springer; 2009. http://link.springer.com/10.1007/978-0-387-84858-7.
32. Lawson P, Sholl AB, Brown JQ, Fasy BT, Wenk C. Persistent Homology for the Quantitative Evaluation of Architectural Features in Prostate Cancer Histology. Scientific Reports 2019 Feb;9(1):1139. https://www.nature.com/articles/s41598-018-36798-y, number: 1 Publisher: Nature Publishing Group.
33. Bishop CM, Nasrabadi NM, Pattern recognition and machine learning. Springer; 2006.

Appendix

Time performance analysis

We asses the time performance of Algorithm 1 by analyzing the worst-case scenario, a complete graph built from a pointcloud $\{x_i\}$ $i \in [1, n]$. This is the worst-case scenario as it contains the maximal number of cliques (hence simplices) for a given number of vertices, namely $2^n - 1$. As discussed in the previous section, the running time will be dominated by the first vertex x_1 as it has the highest number of successive neighbours. The most time consuming operations are the ones that happen inside Algorithm 2, namely the UPDATE fiLTRATION and UPDATE COMMON NEIGHBOURS subroutines.

Update filtration

The extension of a d-clique requires checking whether one or more of the new introduced edges have a filtration value higher than the current d-clique. Comparison between floats can be done in constant time and has to be repeated d times. With reference to figure 18, we can assign to each edge in the simplex tree a cost that depends only on the edge depth. The total sum of such cost is

$$\sum_{i=1}^{n} (i-1)\binom{n}{i} = 2^{n-1}n - 2^n + 1 \quad .$$

In case of perfect parallelization, the cost for the first vertex only is

$$\sum_{i=1}^{n-1} i\binom{n-1}{i} = 2^{n-2}(n-1) \quad .$$

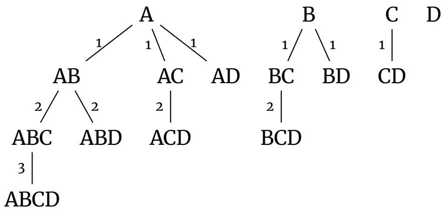

Figure 18. Simplex tree for a 4-clique with the update filtration cost.

Update common neighbours

Updating the list of common neighbours after a clique extension requires computing the intersection between the current list of common neighbours (with length m_1) and the list of neighbours of the newly added vertex (with length m_2). Given that such lists are ordered their intersection can be computed in $\mathcal{O}(m_1 + m_2)$. The total cost for this operation can be obtain recursively by observing that the number of neighbours in a clique is uniquely determined - in this particular case - only by the last element of the clique. For example, in Figure 19 the subtree spanning from AB is the same as the one spanning from B, and the one from AC is equivalent to C. The total cost for a clique of size n can be then expressed as twice the cost for the $(n-1)$−clique plus the cost of the depth 1 edges spanning from the first vertex:

$$c(n) = \frac{(n-1)(n-2)}{2} + 2c(n-1) + (n-1)^2 \quad ;$$
$$c(2) = 1 \quad .$$

Such recurrence has solution

$$c(n) = \frac{1}{2}(-8 + 2^{n+3} - 5n - 3n^2) \quad .$$

In case of perfect parallelization, the cost for the first vertex only is

$$c(n) - c(n-1) = 2^{n+1} - 3n - 1 \quad .$$

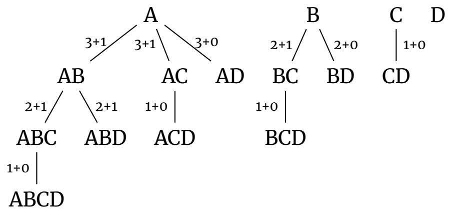

Figure 19. Simplex tree for a 4-clique with the update common neighbours cost.

Memory performance analysis

At each step, the algorithm needs to store in memory the local graph G with each edge's filtration value. The local graph can be stored as an adjacency matrix whose entries represent the filtration values. Moreover, we need to store the current list of simplices, the list of their filtration values and the list of common neighbour for each simplex. Let us denote with V the bits needed to store an edge label (usually a UINT) and with F the bits needed to store a filtration value (usually a flOAT). Assuming the worst case scenario of a fully connected graph with n nodes, the maximum number of simplices will be generated at dimension $\frac{n}{2}$ and will be $\binom{n}{n/2}$. The memory cost at that step will then be $\mathcal{O}(n(n-1)F + \binom{n}{n/2}nV + \binom{n}{n/2}F) = O\left(\frac{2^n}{\sqrt{n}}(nV + F)\right)$, where the first term is the graph cost, the second one is the cost of the list of simplices and the list of common neighbours that we assume to have the same size due to the symmetry of the binomial coefficients, and the third one is the cost of the simplices filtration values.

Response to Reviewers Click here to access/download;Personal Cover;ECP_GigaScience_ reviews.pdf

Dear Editor,

We thank you and the Reviewers for evaluating our manuscript and we are very pleased with the outcome. We took the Reviewers commitment into high consideration and we revised the manuscript accordingly. Please find the revised version attached, the additions in page 1 and 8 are identified by the teal colour.

A point-by-point response to the Reviewers' comments can be found below.

Lastly, we registered our software implementation in bio.tools and our experiments' workflow in workflowhub.eu and added the relevant identifiers to the Code availability section.

Thank you for considering our work, we will be very happy to answer any further questions.

Kind regards,

Davide Gurnari
Paweł Dłotko

============

Reviewer #1: The authors present an insightful analysis of the stability of Euler characteristic curves with respect to the 1-Wasserstein distance. They further introduce a novel extension to a multi-filtration setting termed as the Euler characteristic profile. The authors then offer two distributed algorithms tailored for computing ECC for Vietoris-Rips and cubical complexes. The culmination of their research effort is the vectorization of the Euler characteristic curves and profiles to be utilized as inputs for a classifier. The efficacy of this approach is corroborated using a classification task on a prostate cancer histology slides dataset.

While the paper is cogent and well-organized, there are minor grammatical errors that slightly mar its overall clarity. The introduction of a multi-filtration setting offers fresh insights and can be particularly advantageous for certain tasks. The paper's technical foundation is robust, and the expounded concepts are self-explanatory. The main objective is to compute Euler Characteristic-based features in a distributed manner to cater to big data challenges. Yet, a possible point of contention is the lack of a thorough justification for the need for distributed algorithms. Given that a distributed system setting isn't ubiquitously available in a standard computer science laboratory, the rationale behind this choice could be elaborated upon.

We agree with Reviewer #1 that a distributed system is not available to every practitioner. However, the algorithms we propose are also perfectly parallelizable and our software implementation can take advantage of multi-core CPU that are nowadays the standard even in consumer-grade hardware. For example, the experiment shown in Figure 6 was conducted on an AMD Ryzen Threadripper CPU which can be found on mid-tier desktop workstations. Moreover, time performance benefits can be observed even when running our software on laptop CPUs with “only” 4 or 8 cores.
The need for truly distributed implementations arises when the size of the input data is too large to fit into the system’s memory. Our algorithms can be easily executed on a computer cluster.
We added a comment in the introduction (page 1) to better clarify this aspect.
To summarise, although we are not presenting examples where distributed implementation is required, we hope that the proposed mechanism of stable Euler Characteristics Curves and Profiles will be useful to solve multiple projects among which some large scale projects may arise.

Furthermore, the authors could benefit from explicitly elucidating why streaming algorithms, such as those mentioned in references [15] and [16], aren't directly adaptable to the multi-filtration context.

We thank Reviewer #1 for this comment. The algorithms in [15] and [16] store the cumulative change in the EC at the voxel’s (the top dimensional cells) filtration value.

This requires sorting the voxel’s value from the lowest to highest. While such a sorting can be done for one value filtration, it cannot be performed for a multifiltration, as no good ordering exists in the general case. An appropriate comment has been added to the text in page 8.

While there are certain drawbacks to consider, the TDA community might find value in this research, especially with its exploration of the multi-filtration aspect.

============

Reviewer #2: This paper proposes an Euler characteristic curves based approach as an alternative to persistent homology as a tool in topological data analysis. The approach utilised provides a stable summary of the shape of data which can be computed in a distributed fashion - a key requirement for 'big data' type problems. The approach is novel and builds upon earlier work on Euler characteristic surfaces. The algebraic contribution includes proofs of a number of results which focus exclusively on Betti curves thereby avoiding a complication arising from any attempt to apply the methods used by Chung and Lawson in [24].

Algorithm 1 returns an ordered list of pairs capturing the contribution of each cell at the cell filtration level as well as the sign of the contribution to the Euler characteristic computed as the alternating sum of the number its cells in each dimension.

The authors include an application to cubical complexes utilising the T-construction outlined in Algorithm 3 which offers an alternative to existing approaches and can also be generalized to the multi-parameter filtration case. Algorithms 1 and 3 are easily extended to compute the Euler Characteristic Profile of multi-filtered V-R and cubical complexes. A difficulty is highlighted in the calculation of distances between Euler Characteristic Profiles with a nod towards potential future work searching for more efficient algorithms.

The paper includes a toy example using 3 dimensional ECPs applied to RGB images as well as applications to real world examples involving immune cell spatial patterns in tumours and analysis of prostate cancer slides. They use classification techniques including variations of discriminant analysis as well as support vector machines to compare their algorithms to those utilised in previous studies. Results suggest that the approaches described can offer superior accuracy compared to the literature and this is clear evidence that the work has significant merit and has the potential to be utilised by practitioners of topological data analysis.

The paper is well written and well structured and also includes a useful time & memory performance analysis as an appendix. I recommend that the paper be

accepted and I compliment the authors on the novel application of concepts drawn from the heart of algebraic topology to problems in topological data analysis and image recognition.

In addition, Reviewer #2 highlighted the minor corrections as follows:

- Page 1 last bullet point 'real word data'
- Page 3 Section Stability of Euler Characteristic Curves and Pro-
files '....some know(n) topological quantity'
- Page 6 Section Algorithms '...depending (on) the dimension'
- Page 6 just above Algorithm 1 - I suspect there is a mathematical typo here (-1)^dim(\sigma), f(\sigma)) I think the order of the components in the pair is the wrong way round - suggest asking authors to clarify

We thank Reviewer #2 for the kind comments, we corrected the typos mentioned above.
