# Supplementary material for: Euler characteristic curves and profiles: a stable shape invariant for big data problems
Source: Gigascience. 2023 Nov 15;12:giad094. doi: 10.1093/gigascience/giad094 (PMC10646871; doi:10.1093/gigascience/giad094)
Supplement: giad094_Response_to_Reviewer_Comments_Original_Submission [file giad094_response_to_reviewer_comments_original_submission.pdf]

Author's Response To Reviewer Comments

Dear Editor,

We thank you and the Reviewers for evaluating our manuscript and we are very pleased with the outcome. We took the Reviewers commitment into high consideration and we revised the manuscript accordingly. Please find the revised version attached, the additions in page 1 and 8 are identified by the teal colour.

A point-by-point response to the Reviewers' comments can be found below.

Lastly, we registered our software implementation in bio.tools and our experiments' workflow in workflowhub.eu and added the relevant identifiers to the Code availability section.

Thank you for considering our work, we will be very happy to answer any further questions.

Kind regards,

Davide Gurnari
Paweł Dłotko

============

Reviewer #1: The authors present an insightful analysis of the stability of Euler characteristic curves with respect to the 1-Wasserstein distance. They further introduce a novel extension to a multi-filtration setting termed as the Euler characteristic profile. The authors then offer two distributed algorithms tailored for computing ECC for Vietoris-Rips and cubical complexes. The culmination of their research effort is the vectorization of the Euler characteristic curves and profiles to be utilized as inputs for a classifier. The efficacy of this approach is corroborated using a classification task on a prostate cancer histology slides dataset.

While the paper is cogent and well-organized, there are minor grammatical errors that slightly mar its overall clarity. The introduction of a multi-filtration setting offers fresh insights and can be particularly advantageous for certain tasks. The paper's technical foundation is robust, and the expounded concepts are self-explanatory. The main objective is to compute Euler Characteristic-based features in a distributed manner to cater to big data challenges. Yet, a possible point of contention is the lack of a thorough justification for the need for distributed algorithms. Given that a distributed system setting isn't ubiquitously available in a standard computer science laboratory, the rationale behind this choice could be elaborated upon.

PD-DG: We agree with Reviewer #1 that a distributed system is not available to every practitioner. However, the algorithms we propose are also perfectly parallelizable and our software implementation can take advantage of multi-core CPU that are nowadays the standard even in consumer-grade hardware. For example, the experiment shown in Figure 6 was conducted on an AMD Ryzen Threadripper CPU which can be found on mid-tier desktop workstations. Moreover, time performance benefits can be observed even when running our software on laptop CPUs with "only" 4 or 8 cores. The need for truly distributed implementations arises when the size of the input data is too large to fit into the system's memory. Our algorithms can be easily executed on a computer cluster. We added a comment in the introduction (page 1) to better clarify this aspect.
To summarize, although we are not presenting examples where distributed implementation is required, we hope that the proposed mechanism of stable Euler Characteristics Curves and Profiles will be useful to solve multiple projects among which some large scale projects may arise.

Reviewer #1: Furthermore, the authors could benefit from explicitly elucidating why streaming algorithms,

such as those mentioned in references [15] and [16], aren't directly adaptable to the multi-filtration context.

PD-DG: We thank Reviewer #1 for this comment. The algorithms in [15] and [16] store the cumulative change in the EC at the voxel's (the top dimensional cells) filtration value. This requires sorting the voxel's value from the lowest to highest. While such a sorting can be done for one value filtration, it cannot be performed for a multifiltration, as no good ordering exists in the general case. An appropriate comment has been added to the text in page 8.

Reviewer #1: While there are certain drawbacks to consider, the TDA community might find value in this research, especially with its exploration of the multi-filtration aspect.

============

Reviewer #2: This paper proposes an Euler characteristic curves based approach as an alternative to persistent homology as a tool in topological data analysis. The approach utilised provides a stable summary of the shape of data which can be computed in a distributed fashion - a key requirement for 'big data' type problems. The approach is novel and builds upon earlier work on Euler characteristic surfaces. The algebraic contribution includes proofs of a number of results which focus exclusively on Betti curves thereby avoiding a complication arising from any attempt to apply the methods used by Chung and Lawson in [24].

Algorithm 1 returns an ordered list of pairs capturing the contribution of each cell at the cell filtration level as well as the sign of the contribution to the Euler characteristic computed as the alternating sum of the number its cells in each dimension.

The authors include an application to cubical complexes utilising the T-construction outlined in Algorithm 3 which offers an alternative to existing approaches and can also be generalized to the multi-parameter filtration case. Algorithms 1 and 3 are easily extended to compute the Euler Characteristic Profile of multi-filtered V-R and cubical complexes. A difficulty is highlighted in the calculation of distances between Euler Characteristic Profiles with a nod towards potential future work searching for more efficient algorithms.

The paper includes a toy example using 3 dimensional ECPs applied to RGB images as well as applications to real world examples involving immune cell spatial patterns in tumours and analysis of prostate cancer slides. They use classification techniques including variations of discriminant analysis as well as support vector machines to compare their algorithms to those utilised in previous studies. Results suggest that the approaches described can offer superior accuracy compared to the literature and this is clear evidence that the work has significant merit and has the potential to be utilised by practitioners of topological data analysis.

The paper is well written and well structured and also includes a useful time & memory performance analysis as an appendix. I recommend that the paper be accepted and I compliment the authors on the novel application of concepts drawn from the heart of algebraic topology to problems in topological data analysis and image recognition.

In addition, Reviewer #2 highlighted the minor corrections as follows:

- Page 1 last bullet point 'real word data'
- Page 3 Section Stability of Euler Characteristic Curves and Pro-
files '....some know(n) topological quantity'
- Page 6 Section Algorithms '...depending (on) the dimension'
- Page 6 just above Algorithm 1 - I suspect there is a mathematical typo here (-1)^dim(\sigma), f(\sigma)
) I think the order of the components in the pair is the wrong way round - suggest asking authors to clarify

PD-DG: We thank Reviewer #2 for the kind comments, we corrected the typos mentioned above.
