# Supplementary material for: Euler characteristic curves and profiles: a stable shape invariant for big data problems
Source: Gigascience. 2023 Nov 15;12:giad094. doi: 10.1093/gigascience/giad094 (PMC10646871; doi:10.1093/gigascience/giad094)
Supplement: giad094_Response_to_Reviewer_Comments_Revision_1 [file giad094_response_to_reviewer_comments_revision_1.pdf]

Author's Response To Reviewer Comments

Dear Dr Nogoy,

we carried out all of the required formatting changes. Please find the revised manuscript attached.

Kind regards,
Davide Gurnari
