# Supplementary material for: Euler characteristic curves and profiles: a stable shape invariant for big data problems
Source: Gigascience. 2023 Nov 15;12:giad094. doi: 10.1093/gigascience/giad094 (PMC10646871; doi:10.1093/gigascience/giad094)
Supplement: giad094_Reviewer_1_Report_Original_Submission — Fan Wang, Ph.D -- 9/9/2023 [file giad094_reviewer_1_report_original_submission.pdf]

Reviewer Report

Title: Euler Characteristic Curves and Profiles: a stable shape invariant for big data problems

Version: Original Submission **Date:** 9/9/2023

Reviewer name: Fan Wang, Ph.D.

Reviewer Comments to Author:

The authors present an insightful analysis of the stability of Euler characteristic curves with respect to the 1-Wasserstein distance. They further introduce a novel extension to a multi-filtration setting termed as the Euler characteristic profile. The authors then offer two distributed algorithms tailored for computing ECC for Vietoris-Rips and cubical complexes. The culmination of their research effort is the vectorization of the Euler characteristic curves and profiles to be utilized as inputs for a classifier. The efficacy of this approach is corroborated using a classification task on a prostate cancer histology slides dataset.

While the paper is cogent and well-organized, there are minor grammatical errors that slightly mar its overall clarity. The introduction of a multi-filtration setting offers fresh insights and can be particularly advantageous for certain tasks. The paper's technical foundation is robust, and the expounded concepts are self-explanatory. The main objective is to compute Euler Characteristic-based features in a distributed manner to cater to big data challenges. Yet, a possible point of contention is the lack of a thorough justification for the need for distributed algorithms. Given that a distributed system setting isn't ubiquitously available in a standard computer science laboratory, the rationale behind this choice could be elaborated upon. Furthermore, the authors could benefit from explicitly elucidating why streaming algorithms, such as those mentioned in references [15] and [16], aren't directly adaptable to the multi-filtration context. While there are certain drawbacks to consider, the TDA community might find value in this research, especially with its exploration of the multi-filtration aspect.

Methods

Are the methods appropriate to the aims of the study, are they well described, and are necessary controls included? Choose an item.

Conclusions

Are the conclusions adequately supported by the data shown? Choose an item.

Reporting Standards

Does the manuscript adhere to the journal’s guidelines on minimum standards of reporting? Choose an item.

Choose an item.

Statistics

Are you able to assess all statistics in the manuscript, including the appropriateness of statistical tests used? Choose an item.

Quality of Written English

Please indicate the quality of language in the manuscript: Choose an item.

Declaration of Competing Interests

Please complete a declaration of competing interests, considering the following questions:

- Have you in the past five years received reimbursements, fees, funding, or salary from an organisation that may in any way gain or lose financially from the publication of this manuscript, either now or in the future?
- Do you hold any stocks or shares in an organisation that may in any way gain or lose financially from the publication of this manuscript, either now or in the future?
- Do you hold or are you currently applying for any patents relating to the content of the manuscript?
- Have you received reimbursements, fees, funding, or salary from an organization that holds or has applied for patents relating to the content of the manuscript?
- Do you have any other financial competing interests?
- Do you have any non-financial competing interests in relation to this paper?

If you can answer no to all of the above, write 'I declare that I have no competing interests' below. If your reply is yes to any, please give details below.

I declare that I have no competing interests.

I agree to the open peer review policy of the journal. I understand that my name will be included on my report to the authors and, if the manuscript is accepted for publication, my named report including any attachments I upload will be posted on the website along with the authors' responses. I agree for my report to be made available under an Open Access Creative Commons CC-BY license (http://creativecommons.org/licenses/by/4.0/). I understand that any comments which I do not wish to be included in my named report can be included as confidential comments to the editors, which will not be published.

Choose an item.

To further support our reviewers, we have joined with Publons, where you can gain additional credit to further highlight your hard work (see: https://publons.com/journal/530/gigascience). On publication of this paper, your review will be automatically added to Publons, you can then choose whether or not to claim your Publons credit. I understand this statement.

Yes Choose an item.
