# Supplementary material for: Euler characteristic curves and profiles: a stable shape invariant for big data problems
Source: Gigascience. 2023 Nov 15;12:giad094. doi: 10.1093/gigascience/giad094 (PMC10646871; doi:10.1093/gigascience/giad094)
Supplement: giad094_Reviewer_2_Report_Original_Submission — Liam Naughton -- 9/12/2023 [file giad094_reviewer_2_report_original_submission.pdf]

Reviewer Report

Title: Euler Characteristic Curves and Profiles: a stable shape invariant for big data problems

Version: Original Submission **Date:** 9/12/2023

Reviewer name: Liam Naughton

Reviewer Comments to Author:

This paper proposes an Euler characteristic curves based approach as an alternative to persistent homology as a tool in topological data analysis. The approach utilised provides a stable summary of the shape of data which can be computed in a distributed fashion - a key requirement for 'big data' type problems. The approach is novel and builds upon earlier work on Euler characteristic surfaces. The algebraic contribution includes proofs of a number of results which focus exclusively on Betti curves thereby avoiding a complication arising from any attempt to apply the methods used by Chung and Lawson in [24].
Algorithm 1 returns an ordered list of pairs capturing the contribution of each cell at the cell filtration level as well as the sign of the contribution to the Euler characteristic computed as the alternating sum of the number its cells in each dimension.
The authors include an application to cubical complexes utilising the T-construction outlined in Algorithm 3 which offers an alternative to existing approaches and can also be generalized to the multi-parameter filtration case. Algorithms 1 and 3 are easily extended to compute the Euler Characteristic Profile of multi-filtered V-R and cubical complexes. A difficulty is highlighted in the calculation of distances between Euler Characteristic Profiles with a nod towards potential future work searching for more efficient algorithms.
The paper includes a toy example using 3 dimensional ECPs applied to RGB images as well as applications to real world examples involving immune cell spatial patterns in tumours and analysis of prostate cancer slides. They use classification techniques including variations of discriminant analysis as well as support vector machines to compare their algorithms to those utilised in previous studies. Results suggest that the approaches described can offer superior accuracy compared to the literature and this is clear evidence that the work has significant merit and has the potential to be utilised by practitioners of topological data analysis.
The paper is well written and well structured and also includes a useful time & memory performance analysis as an appendix. I recommend that the paper be accepted and I compliment the authors on the novel application of concepts drawn from the heart of algebraic topology to problems in topological data analysis and image recognition.

Methods

Are the methods appropriate to the aims of the study, are they well described, and are necessary controls included? Choose an item.

Conclusions

Are the conclusions adequately supported by the data shown? Choose an item.

Reporting Standards

Does the manuscript adhere to the journal's guidelines on minimum standards of reporting? Choose an item.

Choose an item.

Statistics

Are you able to assess all statistics in the manuscript, including the appropriateness of statistical tests used? Choose an item.

Quality of Written English

Please indicate the quality of language in the manuscript: Choose an item.

Declaration of Competing Interests

Please complete a declaration of competing interests, considering the following questions:

- Have you in the past five years received reimbursements, fees, funding, or salary from an organisation that may in any way gain or lose financially from the publication of this manuscript, either now or in the future?
- Do you hold any stocks or shares in an organisation that may in any way gain or lose financially from the publication of this manuscript, either now or in the future?
- Do you hold or are you currently applying for any patents relating to the content of the manuscript?
- Have you received reimbursements, fees, funding, or salary from an organization that holds or has applied for patents relating to the content of the manuscript?
- Do you have any other financial competing interests?
- Do you have any non-financial competing interests in relation to this paper?

If you can answer no to all of the above, write 'I declare that I have no competing interests' below. If your reply is yes to any, please give details below.

'I declare that I have no competing interests

I agree to the open peer review policy of the journal. I understand that my name will be included on my report to the authors and, if the manuscript is accepted for publication, my named report including any attachments I upload will be posted on the website along with the authors' responses. I agree for my report to be made available under an Open Access Creative Commons CC-BY license (http://creativecommons.org/licenses/by/4.0/). I understand that any comments which I do not wish to

be included in my named report can be included as confidential comments to the editors, which will not be published.

Choose an item.

To further support our reviewers, we have joined with Publons, where you can gain additional credit to further highlight your hard work (see: https://publons.com/journal/530/gigascience). On publication of this paper, your review will be automatically added to Publons, you can then choose whether or not to claim your Publons credit. I understand this statement.

Yes Choose an item.
